# Recommendations for interdisciplinary research collaboration for early career dissemination and implementation researchers: A multi-phase study

**DOI:** 10.1017/cts.2024.684

**Published:** 2025-01-17

**Authors:** Hannah G. Lane, Sallie D. Allgood, Julie Schexnayder, Hayden B. Bosworth, Ana A. Baumann, Allison A. Lewinski

**Affiliations:** 1 Department of Population Health Sciences, Duke University School of Medicine, Durham, NC, USA; 2 Duke University School of Nursing, Durham, NC, USA; 3 University of Alabama at Birmingham School of Nursing, Birmingham, AL, USA; 4 Division of General Internal Medicine, Department of Medicine, Duke University School of Medicine, Durham, NC, USA; 5 Department of Psychiatry and Behavioral Sciences, Duke University School of Medicine, Durham, NC, USA; 6 Durham Center of Innovation to Accelerate Discovery and Practice Transformation, Durham Veterans Affairs Health Care System, Durham, NC; 7 Division of Public Health Sciences, Department of Surgery, Washington University in St. Louis, St Louis, MO, USA

**Keywords:** Implementation science, health services research, intersectoral collaboration, interdisciplinary research, program development, information dissemination

## Abstract

**Introduction:**

Dissemination and implementation (D&I) scientists are key members of collaborative, interdisciplinary clinical and translational research teams. Yet, early career D&I researchers (ECRs) have few guidelines for cultivating productive research collaborations. We developed recommendations for ECRs in D&I when serving as collaborators or co-investigators.

**Methods:**

We employed a consensus-building approach: (1) group discussions to identify 3 areas of interest: “Marketing yourself” (describing your value to non-D&I collaborators), “Collaboration considerations*”* (contributions during proposal development), and “Responsibilities following project initiation” (defining your role throughout projects); (2) first survey and focus groups to iteratively rank/refine sub-domains within each area; (3) second survey and expert input on clarity/content of sub-domains; and (4) iterative development of key recommendations.

**Results:**

Forty-four D&I researchers completed the first survey, 12 of whom attended one of three focus groups. Twenty-nine D&I researchers completed the second survey (*n* = 29) and 10 experts provided input. We identified 25 recommendations. Findings suggest unique collaboration strengths (e.g, partnership-building) and challenges (e.g., unclear link to career milestones) for ECR D&I researchers, and underscore the value of ongoing training and mentorship for ECRs and the need to intersect collaborative D&I efforts with health equity principles.

**Conclusions:**

Research collaborations are essential in clinical and translational research. We identified recommendations for D&I ECRs to be productive research collaborators, including training and support needs for the field. Findings suggest an opportunity to examine research collaboration needs among early career D&I scientists, and provide guidance on how to successfully provide mentorship and integrate health equity principles into collaborative research.

## Introduction

Timely implementation of evidence-based practices is critical to improve health care delivery and health outcomes across many disciplines and specialties, yet the time lag between research inception and translation of useful findings to common practice can be lengthy, and many worthwhile innovations are never fully implemented. Additionally, research intended to generate evidence may remain unpublished, rely upon outdated policies and practices, or not align with health system policies and priorities [1,2]. Dissemination and implementation (D&I) science provides a collection of research designs and methodologies to help clinicians and researchers translate evidence-based practices, interventions, and policies into real-world, sustained practice [1]. D&I science holds promise for reducing research waste by accelerating the uptake of new, evidence-based solutions to complex health and healthcare delivery problems [3,4,5]. As a result, federal and nonfederal funders across many disciplines increasingly require the use of D&I science in studies and projects, thus increasing the demand for D&I experts who can support these undertakings through collaboration or consultation [6].

The skills and responsibilities of a research collaborator or project/study consultant differ from those of a principal investigator. The current demand for seasoned D&I collaborators exceeds supply, despite a growing number of training programs to support D&I science competency development [7,8,9,10,11,12]. These programs are primarily focused on training individuals as independent investigators capable of applying for investigator-initiated D&I funding. Given the current demand, early career researchers (ECRs; i.e., individuals within 10 years of terminal degree or postdoctoral fellowship completion) are often approached to serve as D&I consultants and collaborators, despite potentially lacking skills or training specific to research collaborations, particularly with principal investigators in other fields or disciplines.

The paucity of guidance on building D&I-focused research collaborations is surprising given the breadth of challenges to interdisciplinary research [13,14] that have been characterized in the D&I literature. Recognizing the unmet need for collaborative research support, Tabak et al [6]. outlined hypothetical scenarios that D&I researchers are likely to encounter when working with scientists unfamiliar with D&I concepts and methods, and offered practical suggestions on the appropriateness of study team roles (e.g., when the expected scope of D&I contributions indicates the need for a consultant versus a co-investigator role).

Our project was prompted by a guided discussion of the Tabak et al article during an interdisciplinary D&I journal club supported by a training grant **(**K12HL138030). This journal club is led by a senior D&I scientist and largely attended by ECRs, with representation of multiple academic institutions, healthcare systems, and academic disciplines. Prompted by the following four questions, attendees discussed how the collaboration scenarios and recommendations outlined by Tabak et al., might apply to ECRs:

1. What are the marketable skills of an early career D&I researcher?
2. What questions should an early career D&I researcher ask when approached about becoming a research collaborator?
3. What are the responsibilities of a D&I collaborator during the grant-writing process?
4. What range of responsibilities should a D&I collaborator be willing to accept if the grant obtains funding?

Journal club attendees responded to these prompts in real time using Google’s virtual Jamboard platform. ECRs reported that they desired guidance on how to better communicate their D&I skillset to scientists in other disciplines, how to advocate for themselves to serve as funded co-investigators on grants, and how to negotiate for the resources needed for successful execution of D&I research activities as part of larger grants. This discussion confirmed a gap in the D&I literature pertaining to guidance for cultivating and sustaining effective interdisciplinary research collaborations and affirmed that ECRs viewed engagement in collaborative research as essential for developing their broader D&I expertise.

The purpose of this study, therefore, was to identify a consensus-derived set of collaboration recommendations for early career D&I researchers and to develop guidance that can help ECRs more effectively collaborate with scientists across a range of research disciplines.

## Materials and methods

**Figure 1.**
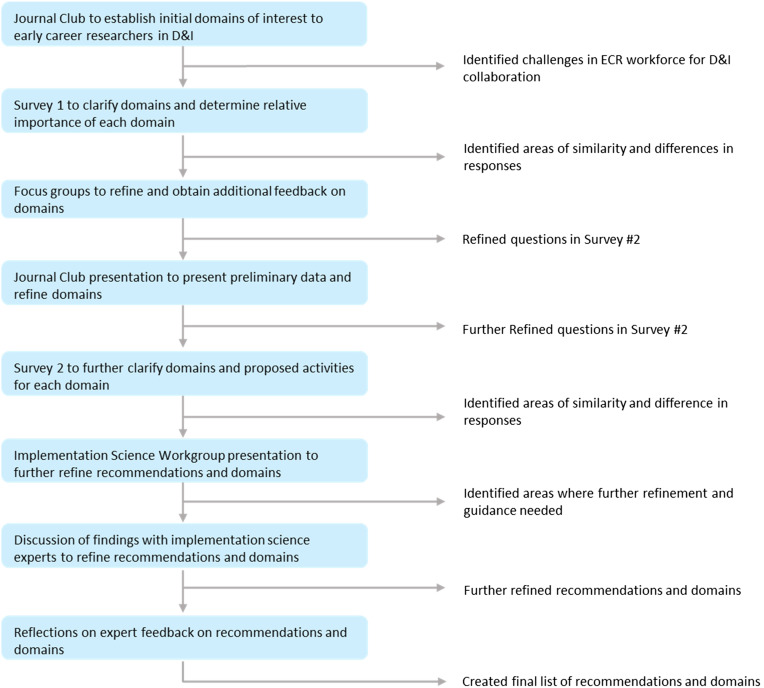
Study flow diagram. Legend: ECR = early career researcher; D&I = dissemination and implementation.

This iterative, systematic, multi-phased study (Figure 1) used a consensus-building approach previously applied in the D&I literature [15,16,17] to identify recommendations for research collaborations specifically for ECRs specializing in D&I. All study activities were reviewed and determined exempt by the Duke University Health System Institutional Review Board (Pro00108634). Supplementary File 1 details our decision process across phases. Supplementary File 2 includes the survey instruments and Supplementary File 3 includes guiding slides and script for our focus groups.

### Phase 1: Development of D&I collaboration domains

Four authors of this manuscript (HL, SDA, JS, AAL – all ECRs) reviewed and summarized the general comments raised during the journal club, identifying three distinct areas of attention for ECRs as they establish their D&I career [1]: “Marketing yourself” (i.e., describing your value to potential non-D&I research collaborators in order to locate and maintain engagement with collaborators) [2], “Collaboration considerations” (i.e., clarifying expected contributions of the D&I collaborator during proposal development), and [3] “Responsibilities once project is initiated” (i.e., defining your role during project execution). With guidance from a D&I expert (HBB), the four authors thematically grouped journal club responses into 12 domains across these three areas.

### Phase 2: Refinement of D&I collaboration domains via consensus-building

Phase 2 consisted of building consensus among other D&I scientists on the 12 initial domains and constructing preliminary recommendations through a survey and series of focus groups.

### Survey 1

#### Overview

We designed a descriptive survey (Survey 1; Supplementary File 2) based on prior studies [18,19] to determine the clarity of the initial domains and their relative importance to interdisciplinary research collaborations. The authors distributed this survey to several D&I colleagues to test its wording and clarity. We then pilot-tested the survey with input from several D&I experts before deploying it via Qualtrics in early summer 2021.

#### Recruitment

We sought to recruit a mix of ECRs and senior researchers with varied experiences in D&I (i.e., principal investigators, collaborators, and mentors). We used convenience and snowball sampling to recruit participants via listservs and emails to colleagues. All survey responses were anonymous. On completion of the survey, participants could provide their contact information to be entered to win a $50 gift card.

#### Survey items

The survey consisted of 24 items that solicited demographic information and participants’ reactions to the domains identified in Phase 1. Participants were asked to (a) rate the importance of each associated domain (*n* = 12) on a 3-point scale (1 = *not that important* to 3 = *very important*) to effective research collaborations with investigators from other disciplines, and (b) describe via open-ended text any needed clarifications of the existing domains. Participants could also propose new domains.

#### Analysis plan

We used descriptive statistics (e.g., frequencies, means) to describe ratings, and two team members independently reviewed, summarized, and analyzed the open-ended responses. The authors met as a group to review findings and achieve consensus on which domains required further development (Supplementary File 1).

### Focus groups

#### Overview

We conducted virtual focus groups to further explore Survey 1 findings and generate additional feedback on the initial domains. Three focus groups intending to last approximately 45 minutes occurred via Zoom sequentially, approximately one week apart, in late summer 2021.

#### Recruitment

Survey 1 participants were asked to indicate their interest in focus group participation. Those who expressed interest were contacted via email to confirm participation. Participants received $10 gift cards.

#### Structure

A moderator and two notetakers from the authorship team conducted each focus group. The moderator established ground rules for discussion and obtained verbal consent to record. Focus groups were audio recorded, but the recordings were not transcribed; notetakers summarized key discussion points in real-time. The focus group interview guide was developed based on the survey findings (Supplementary File 3). The focus groups were iterative, with each discussion informed by prior group findings.

#### Analysis Plan

We analyzed data using rapid qualitative analysis with purposeful data reduction techniques [20,21,22]. At the end of each focus group, the moderator and notetakers held a debriefing session to discuss findings and to identify topics for discussion in subsequent focus groups. Debriefing sessions were audio recorded but not transcribed. We created a summary of findings for each domain based on our detailed focus group notes and summaries of the debriefing discussions. Summaries were integrated with the survey results after completion of the three focus groups (Supplementary File 1).

We presented select findings to the D&I journal club attendees, who provided feedback for further refinement. By the end of Phase 2, the team compiled 29 activities within 9 refined domains (reduced from the 12 initial domains). Within each domain, we also began to identify specific ECR activities or behaviors that could promote more successful research collaborations (Supplementary File 1).

### Phase 3: Refinement of D&I collaboration domains and activities

#### Overview

Phase 3 consisted of a second survey (Survey 2; Supplementary File 2) that was designed to further clarify domains and transition proposed activities into specific recommendations. The survey was conducted via Qualtrics in early fall 2021.

#### Recruitment

As with Survey 1, we sought to achieve a mix of senior researchers and ECRs with varied experiences in D&I. We used convenience and snowball sampling to recruit participants via listservs, emails disseminated through colleagues, and social media. Participants who completed Survey 1 were eligible to complete Survey 2. At the end of the survey, participants could provide their contact information to win one of several $50 gift cards.

#### Survey items

The survey consisted of 39 items pertaining to (a) demographic information, and (b) reactions to the 9 refined domains and 29 proposed activities. We randomized the order in which the areas were presented in order to minimize participant burden and increase the likelihood of obtaining adequate responses for each area. For each domain, we asked respondents to “Indicate if you recommend changes and, if so, describe what is needed to provide additional clarity.” Respondents could then suggest additional domains or activities they considered essential to effective D&I research collaborations.

#### Analysis plan

We used the same processes described in Survey 1 to analyze the quantitative and qualitative data, including assessing recommendations for changes to content and clarity within each domain. As part of Phase 3 analysis, we presented select findings to an implementation science workgroup (consisting of D&I scientists, practitioners, and research staff) to ensure the findings’ sufficiency to guide additional changes to our domains and associated activities. Phase 3 analysis is described in Supplementary File 1.

### Phase 4: Development of final list of domains and recommendations

In Phase 4, we further refined each domain and drafted relevant recommendations related to each activity using Bloom’s taxonomy [23] to ensure that our recommendations could ultimately be translated to achievable learning outcomes. The list of recommendations underwent 3 iterative rounds of review by members of the authorship team (HL, SA, JS, AA) to eliminate duplicative language, refine wording, confirm fit within the underlying domain, and assess coverage of the 3 original areas of focus. Additionally, during this review, we developed data-driven guidance for actions or activities related to each recommendation. Finally, we engaged two D&I experts (AB, HBB) to provide feedback on the domains, recommendations, and guidance. In particular, AB provided perspective on intersections of D&I and health equity research that emerged from our recommendations, and HBB provided perspective on mentoring ECRs in health services research and implementation science.

## Results

**Figure 2.**
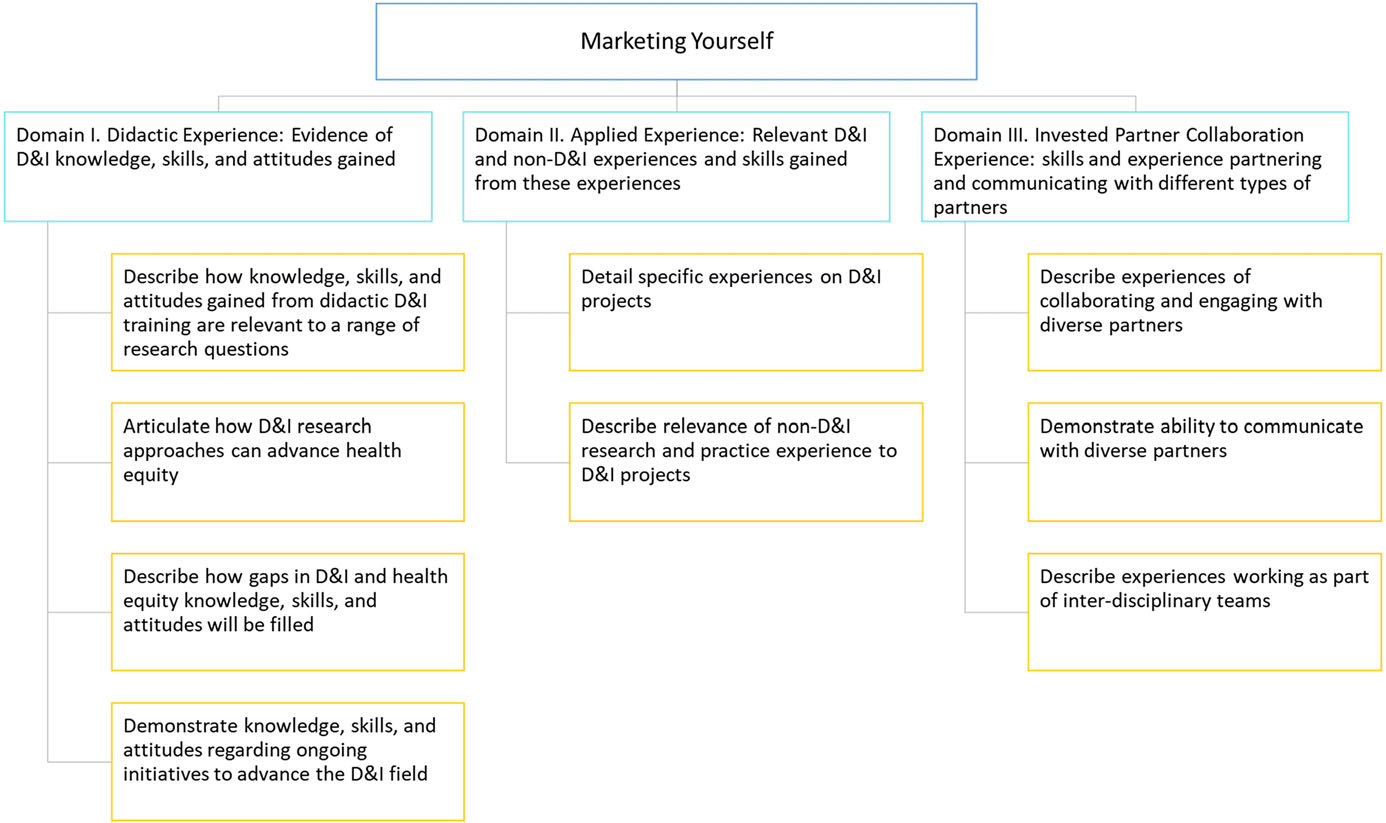
“Marketing Yourself” domains and recommendations. Legend: D&I = dissemination and implementation.

**Figure 3.**
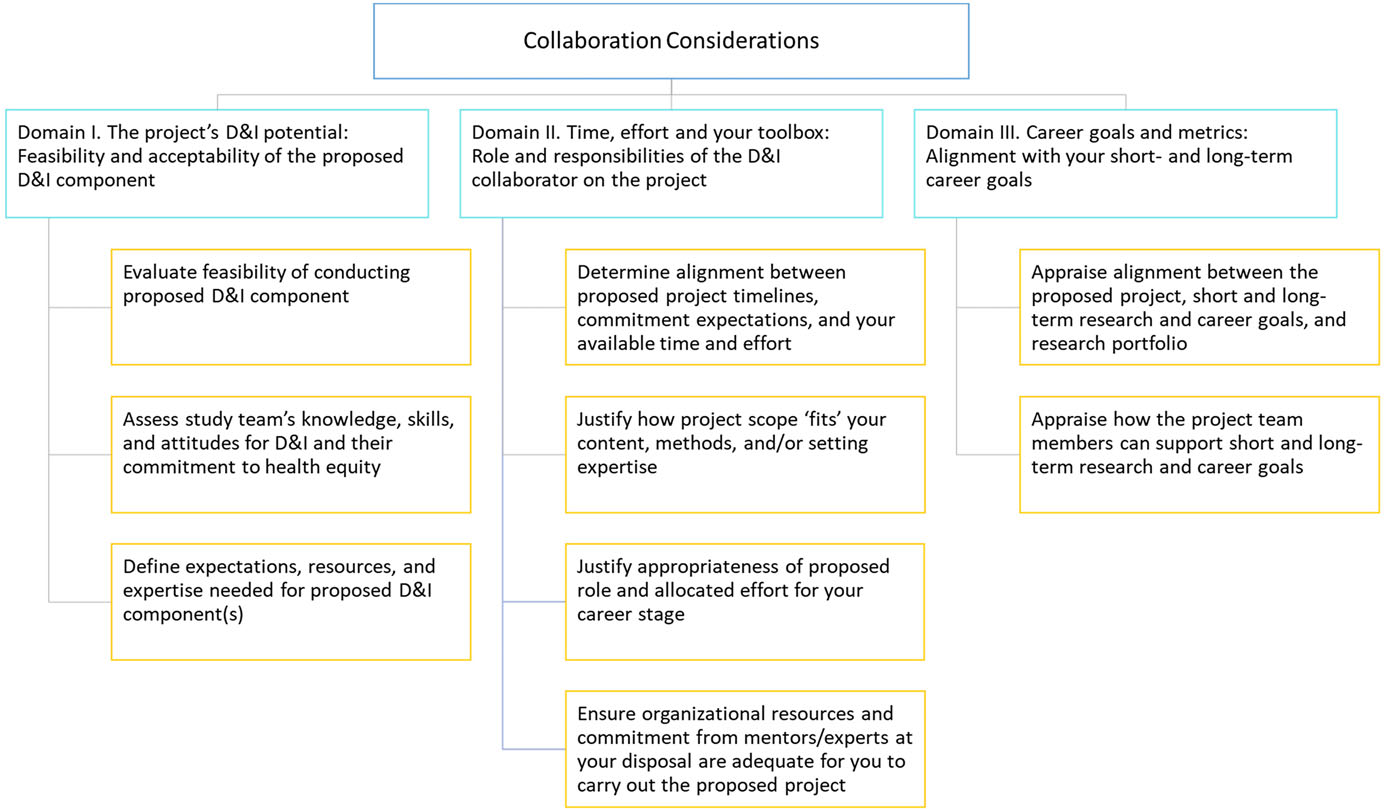
“Collaboration Considerations” domains and recommendations. Legend: D&I = dissemination and implementation.

**Figure 4.**
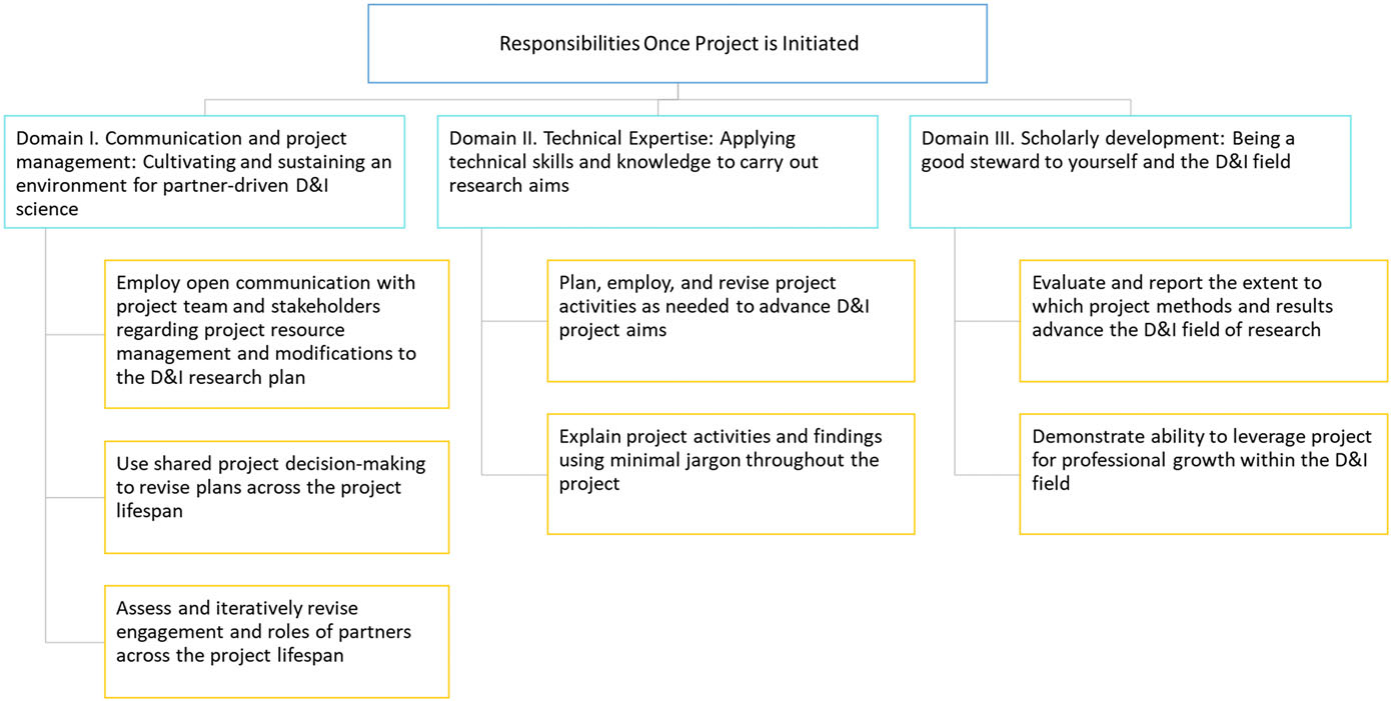
“Responsibilities Once Project is Initiated” domains and recommendations. Legend: D&I = dissemination and implementation.

Herein we describe findings from Phases 2–4 of the study. The final list of domains and recommendations are described in Figures 2–4 and Tables 1–3. A more detailed description of our results across phases is described in Supplementary File 1.

**Table 1.** “Marketing Yourself” guidance and examples by domain

Recommendations	Guidance to achieve recommendations
**Domain I. Didactic Experience: Evidence of D&I knowledge, skills, and experiences**	
Describe how knowledge, skills, and attitudes gained from didactic D&I training are relevant to a range of research questions	Demonstrate ability to apply knowledge, skills, and experiences obtained in courses and/or training to a range of D&I research questions. • Describe foundational D&I knowledge, skills, and experiences obtained through formal courses and/or training. Include specifics of training content (e.g., methods, theory and frameworks, implementation strategies, etc.) and the entity that provided, developed, and/or endorsed the training (e.g., VA, NIH).
Articulate how D&I research approaches can advance health equity	Demonstrate understanding of equity-informed research practices and describe how to integrate and apply equity-informed D&I research practices. Articulate importance of fidelity and adaptation of interventions to diverse populations.
Describe how gaps in D&I and health equity knowledge, skills, and attitudes will be filled	Describe how resources, mentorship, and networks available to you could be leveraged to fill gaps in knowledge or skills.
Demonstrate knowledge, skills, and attitudes regarding ongoing initiatives to advance the D&I field	Articulate how you keep up with advances in the D&I field and apply these advances in your work. • Describe involvement in field (e.g., memberships, workgroups, grants, publications, etc.).
**Domain II. Applied Experience: Relevant D&I and non-D&I experiences and skills gained from these experiences**	
Detail specific experiences on D&I projects	Describe details of your D&I research and/or practice experience. • Describe prior D&I role(s) (e.g., researcher, practitioner, consultant, advisor, educator, mentor, etc.). • Provide examples of specific research skills gained (e.g., proposal development, use of theory/framework, data preparation, collection, analysis, evaluation, technical assistance, disseminating findings, etc.). • Identify experiences integrating health equity research principles in these projects.
Describe relevance of non-D&I research and practice experience to D&I projects	Describe how clinical, professional, lived, and/or other expertise applies to D&I research projects: • Content expertise (e.g., community engagement strategies, clinical or lived experience, etc.) • Methodological expertise (e.g., qualitative interviewing and analysis, program evaluation, quality improvement, etc.) • Contextual understanding (e.g., community history, assets, needs) • Experiential expertise (e.g., clinical or lived experience in the setting)
**Domain III. Invested Partner Collaboration Experience: Skills and experience partnering and communicating with different types of partners**	
Describe experiences collaborating and engaging with diverse partners	Describe how prior interaction and experience with invested partners is relevant to the project. Include details of partners engagement, including the following: • Breadth (e.g., operations, community-based organizations, practitioners, clinicians, academics) • Depth (e.g., duration of partners’ engagement, roles/activities engaged in) • Experiences with equity and diversity (e.g., engaging diverse partners, using equity-driven approaches to relationship building, prioritizing diverse representation in partnerships, etc.)
Demonstrate ability to communicate with diverse partners	Articulate specific examples of communicating with diverse audiences throughout project phases, including examples involving the following: • Role (e.g., implementation researchers, implementation practitioners, implementers, etc.) • Identity (e.g., race, gender, age, sexual orientation, organization represented)
Describe experiences working as part of interdisciplinary teams	Articulate how team science principles were upheld to complete a project. This could include specification about prior leadership roles and activities, experiences using shared decision-making, program co-development, community-engaged research, etc.

Legend: ECR = Early Career Researcher; D&I: Dissemination and Implementation; VA: Veteran’s Administration’; NIH: National Institutes of Health.

**Table 2.** “Collaboration considerations” guidance and examples by domain

Recommendations	Guidance to achieve recommendations
**Domain I. The project’s D&I potential: Feasibility and acceptability of the proposed D&I component**	
Evaluate feasibility of conducting proposed D&I component	Critically evaluate the rationale for the D&I project and determine whether the scope of the proposed D&I project is feasible in the context of the project proposed. • Determine availability and validity of preliminary data, grant rules/guidelines, scope of proposed D&I activities compared to other project activities.
Assess study team’s knowledge, skills, and attitudes for D&I and their commitment to health equity	Assess team’s knowledge, skills, experience, and appetite for D&I research to understand their views on and commitments to D&I aim activities. Assess the team’s expertise and commitment to health equity and willingness to fill gaps in expertise. • Consider opportunities to engage end users (e.g., patients, family members, community members, etc.) in D&I outcomes (e.g., feasibility, acceptability) to provide D&I outcomes that are not limited to the provider-level.
Define expectations, resources, and expertise needed for proposed D&I component(s)	Determine alignment between D&I aims, data, and resources allocated in the project proposal and the project personnel. • Consider personnel effort and expertise, project timeline, resources needed to complete activities, access to project data, impact of D&I findings on study approach, authorship of products. • Consider agreements with and compensation for involvement of invested partners (particularly with under-represented collaborators)
**Domain II. Time, effort, and your toolbox: Role and responsibilities of the D&I collaborator on the project**	
Determine alignment between proposed project timelines, commitment expectations, and your available time and effort	Discuss project deadlines and expectations to ensure these are reasonable • Take into account that the amount of time needed to complete activities may be longer for early career researchers as you learn and refine your research skills. Clarify professional collaboration expectations as a member of the team (this is in addition to specific work on D&I components). • Meeting schedule, preparation, expectations for attendance • Collaboration record of project team and their interactions with community partners
Justify how project scope “fits” your content, methods, and/or setting expertise	Articulate how each project component leverages your expertise • How your content, context, and methodological skills can be applied across all project phases
Justify appropriateness of proposed role and allocated effort for your career stage	Review scope of work needed to conduct D&I activities and consider how this aligns with the proposed role and %FTE • Alignment between proposed role on and substantive contribution to the project. (e.g., qualitative interviewer = study staff, consultant; aims development, oversight, and management = Co-investigator, Principal Investigator, Multiple principal investigators) • Advocate for roles and project activities (including mentored activities) that will enable contributions beyond what might be expected for your career stage
Ensure the organizational resources and commitment from mentors/experts at your disposal are adequate for you to carry out the proposed project	Determine project- or non-project related resources available to you within your organization • Consider partnerships or resources that can be immediately/easily leveraged vs. those that need to be developed/built. • Consider how existing staff within could be re-allocated to meet proposed skills and % effort.
**Domain III. Career goals and metrics: Alignment with your short- and long-term career goals**	
Appraise alignment between the proposed project, short and long-term research and career goals, and research portfolio	Determine tangible return from proposed participation in the project activities, aims, and products (e.g., gain or refine skills, pilot data for grant, use dissemination methods to further own research, obtain continuous funding, gain progressive leadership experience). Determine how this project supports/helps to meet metrics for career advancement at your current institution: • Opportunities for lead and co-authorship throughout the grant, timing of project activities with promotion, mentorship of junior researchers
Appraise how the project team members can support short and long-term research and career goals	Determine how involvement with principal investigators and team supports career goals and aligns with institutional promotion and tenure requirements (e.g., mentorship in specific area, networking with new partners, continued collaboration for future projects)

Legend: D&I = Dissemination and Implementation; FTE = Full-Time Equivalent.

**Table 3.** “Responsibilities Once Project is Initiated” guidance and examples by domain

Recommendations	Guidance to achieve recommendations
**Domain I. Communication and project management: Cultivating and sustaining an environment for partner-driven D&I science**	
Employ open communication with project team and invested partners regarding project resource management and modifications to the D&I research plan	Reconfirm scope of D&I work and resources needed at project start up and throughout the duration of the project. • Discuss funding/effort modifications as needed, changes in scope of work, adaptations of D&I activities Communicate frequently with team members and partners across all levels to identify challenges. • Identify which meetings you should attend to provide the D&I perspective. • Identify which meetings partners should attend. • Provide updates on D&I activities and reference D&I framework/s (in as simple terms as possible) to guide the work to provide context and engage partners in D&I activities. Adapt D&I vocabulary to meet needs of project team and partners.
Use shared project decision-making to revise plans across the project lifespan	Adapt resource commitments to changing project demands. • Collaboratively modify project plans (e.g., resource allocation, effort) in response to identified challenges.
Assess and iteratively revise engagement and roles of partners across the project lifespan	Routinely clarify invested partners’ roles in project decision-making. • Ensure equitable funding provided for partners’ project activities; consider FTE allocation for key operation partners. • Minimize invested partners’ burden. • Include partners in presentation of findings and formal dissemination plans.
**Domain II. Technical Expertise: Applying technical skills and knowledge to carry out research aims**	
Plan, employ, and revise project activities as needed to advance D&I project aims	Apply D&I principles and methods to project activities and demands. • Ensure the D&I framework continues to guide project protocols and activities. Identify project milestones and timeline for D&I activities. Use resources at your disposal to complete project activities: • Data management support, statistical assistance, mentorship, workshops/seminars, other funding mechanisms; dissemination of findings Be prepared to adapt protocols to planned and unplanned project demands.
Explain project activities and findings using minimal jargon throughout the project	Provide clear explanations of activity and findings: • Explain D&I terminology and articulate concepts in simple terms. • Elicit feedback from partners and project team on clarity of communication Disseminate findings in real time, as appropriate, to the project team to support D&I project aims.
**Domain III. Scholarly development: Being a good steward to yourself and the D&I field**	
Evaluate and report the extent to which project methods and results advance the D&I field of research	Determine expectations of journals, conferences, and funders for publications and future research Identify and disseminate innovative methods, activities, and/or findings throughout the project • Publish peer-reviewed manuscript on methods, recruitment, and/or other activities. • Disseminate lessons learned from interdisciplinary D&I collaborations within sphere of influence.
Demonstrate ability to leverage project for professional growth within the D&I field	Take on progressive leadership roles to contribute to the growth of others. • Mentor other scientists and practitioners in D&I. Integrate skills and knowledge learned during the project into future D&I work. • Develop D&I trainings.

Legend: D&I = Dissemination and Implementation; FTE = Full-Time Equivalent.

### Sample

Survey 1 received 47 responses of which 44 (94%) were sufficiently complete to include in the analysis. Most respondents self-identified as cis-women (82%), white (84%), and non-Hispanic (95%); and half (50%) identified as ECRs. Most (87%) identified as a D&I researcher, and 74% reported having D&I training or experience. Forty-three percent reported having served as principal investigator on a D&I project, 69% as having collaborated on a D&I project, and 48% as having mentored D&I ECRs. Of the 44 respondents, 16 indicated interest in attending a focus group, and 12 attended one of the three focus groups (range of attendees *n* = 3–5; average length = 47 minutes). Focus group participants included a mix of ECRs and experienced researchers.

Survey 2 received 49 responses, 29 (59%) of which were sufficiently complete to include in this analysis. Of the 29 participants, 69% identified as cis-women, 90% as white, and 90% as non-Hispanic. All identified as D&I researchers, 62% as ECRs. Fifty-two percent had served as principal investigator on a D&I project, 52% had collaborated on a D&I project, and 34% had served as a consultant on a D&I project.

### Refining domains

Herein we briefly describe the refinement process across the three areas. Supplementary File 1 provides additional details.

### Marketing yourself

In Survey 1, most respondents rated domains in this area as “very important”; however, the domain *describing specific D&I training* was rated “very important” by only slightly over half of respondents. In open-ended text, respondents suggested adding the following domains [1]: access to D&I resources and mentors [2], experience working with operational/community partners, and [3] communication skills related to D&I terminology.

In focus groups, the relative importance of *describing specific D&I training* sparked considerable debate. Several participants noted that although training is important, it may be less accessible than applied experience, particularly for under-represented ECRs, and therefore should not be viewed as more valuable than experience. Focus groups also suggested the need for recommendations within an additional domain: describing one’s communication-related skills and experiences with both research teams and operational partners.

In Survey 2, participants provided suggestions for clarity and/or content modifications for all 10 activities within this domain. Suggested recommendations pertained to knowledge of health equity, knowledge of international D&I landscape, and experience as D&I consultants and/or trainers.

### Collaboration considerations

In Survey 1, nearly all respondents (98%) ranked *defining your role*, as “very important.” In open-ended text, respondents [1] indicated confusion and overlap regarding the *principal investigator’s commitment to the project* [2]; emphasized the need to assess how the D&I components of a project will be appropriately resourced (e.g., staff time and effort, support for implementation activities and partnership-building); and [3] identified that most domains in this area were important for collaboration in general but not specific to the D&I field.

In focus groups, the importance of clarifying roles and team dynamics dominated the discussion. Participants outlined many functions that a D&I collaborator could potentially serve (e.g., a “go between” person connecting study team with operational partners; delivery agent; instrument developer; data collector on a project, grant, or study). Participants emphasized the importance for ECRs of avoiding common collaborator “traps” (e.g., over-involvement in data collection, not requesting sufficient resources to complete the D&I work, being seen as a team member rather than team leader) that could impede career advancement. In addition, several participants noted the need for frequent communication and “expectation management” between ECR D&I collaborators and primary study principal investigators who do not have D&I expertise.

In Survey 2, clarity and content edits were suggested for eight of the nine activities. Suggestions included aligning one’s role/effort with your title, garnering peer support for D&I work, troubleshooting challenges, and communicating one’s contribution to non-D&I experts.

### Responsibilities once project is initiated

In Survey 1, most respondents scored *working well with others* as “very important” but suggested in open-ended text that this was not specific to D&I collaborations. Open-ended text also indicated that *setting expectations* was more appropriate for the Collaboration Considerations area and that having access to mentors and resources, strong partnerships, and continuous learning was important across all stages of collaboration.

Focus groups suggested revision of existing domains related to project and resource management, and incorporation of an explicit domain focused on mentoring, partnerships, and learning. Participants indicated the importance of *a priori* communication with the principal investigator about both the iterative nature of D&I research and the expectation that refinement of the scope of the D&I component may be inevitable. Although attendees agreed on the importance of mentorship and teaching for ECRs across the research continuum, they had mixed opinions regarding whether, when, and how ECRs should engage as mentors and teachers. It was noted that because few institutions have a “deep bench” of D&I scientists, such responsibilities may inappropriately fall to ECRs.

In Survey 2, recommendations for clarity and/or content edits were made for all nine activities. Suggested additions included time and resource management related to strategic partner involvement, networking and mentoring, and science communication.

### Findings across 3 areas

Several interdependent topics spanned the three areas, including building and sustaining partnerships, navigating team dynamics, and communicating clearly and effectively to multiple types of partners. The importance of being able to articulate the “value added” of a D&I skillset for a given project was also noted, as was the need to consider health equity concepts. A particularly salient cross-cutting topic was when and how to seek mentorship from more senior D&I experts during the collaboration process; participants viewed this understanding as critically important for ECRs attempting to implement our collaboration recommendations. These topics were incorporated into various recommendations to reflect their importance across research stages.

### Finalizing the set of recommendations

The collaborative consensus-building process elicited D&I collaboration behaviors that align with the 3 ECR-identified areas of interest from journal club comments. The 25 total recommendations, as well as the guidance to achieve them based on our data, are detailed in the accompanying figures and tables. The first area, *Marketing yourself,* contains 3 domains and 9 recommendations (Figure 2 **,** Table 1). The second area, *Collaboration considerations,* consists of 3 domains and 9 recommendations (Figure 3 **,** Table 2). The third area, *Responsibilities once project is initiated,* consists of 3 domains and 7 recommendations (Figure 4 **,** Table 3).

## Discussion

Translational research collaborations involving D&I scientists, both within and outside their disciplines, are becoming increasingly common and form a critical component of an ECR’s career development and advancement [6]. Through an iterative, multi-phase consensus-building process, we identified specific recommendations that can help ECRs build productive research collaborations to advance D&I science. Our data support that (a) interdisciplinary research collaborations are critical and likely to occur throughout a D&I scientist’s career, (b) such collaborations hold a unique set of challenges due to the interdisciplinary nature of D&I, and (c) mentorship is critical to obtaining the skills needed to collaborate effectively as a D&I scientist.

### Interdisciplinary research collaborations are critical and likely throughout a D&I scientist’s career

Invitations to participate in collaborative D&I research can greatly benefit ECRs professionally but may come at a time of transitioning from pre- or postdoctoral training to formalized research appointments [6]. Critically, there remains limited guidance for ECRs on cultivating productive research collaborations with scientists who are unfamiliar with D&I concepts or methods [6,7]. The lack of guidance may be due in part to the underrepresentation of recommendations for collaboration among researchers in existing D&I competency frameworks. To date, various competencies for D&I areas have been published: D&I science [9,24,25,26], implementation practice [8,27,28], knowledge translation [29,30], and learning health systems [18]. Knowledge and skills related to research collaborations have been previously identified as obligate competencies for D&I researchers [8]; however, in most D&I competency frameworks, the primary collaboration-related focus is on fostering research-practice partnerships and community partner engagement in D&I research [24,26,28,29]. Findings from this study suggest that the skills needed for ECRs to engage in and sustain research collaborations with non-D&I scientists may differ, at least in part, from the knowledge, skills, and other abilities needed to engage in and sustain relationships with practice partners. Such skills should be incorporated into D&I training and mentoring.

### Collaboration has benefits but also poses challenges for D&I ECRs

Our findings suggest that ECRs embarking on a research career in a team-based and interdisciplinary field such as D&I can expect challenges and benefits. Benefits of the highly interdisciplinary nature of D&I work include a breadth of literature to guide research and practice, senior mentors willing and able to usher in a new generation of D&I scientists, and funding to add D&I scientists to grants and projects. In both the *Collaboration considerations* and *Responsibilities* areas, study participants identified myriad roles and functions a D&I collaborator could potentially serve (and roles they should not serve) to benefit themselves and the project. Additionally, the nature of D&I research enables nontraditional products and dissemination channels that can expedite and expand the impact of research findings. We identified several cross-cutting recommendations across the three areas, including building and sustaining partnerships, navigating team dynamics, and communicating clearly and effectively to researchers with limited D&I expertise. Similar to Tabak et al., we recommend being upfront in defining and establishing role expectations and ensuring alignment with the ECR’s skillset, available resources, and career trajectory [6].

Challenges exist that should be acknowledged. For example, ECRs should ensure that the scope of the D&I work is realistic given the proposed D&I aims and allocated resources and effort. D&I research is complex; thus our recommendations provide guidance on how to approach the principal investigator in the context of the project and the ECR’s professional advancement, craft reasonable research questions, and advocate for adequate resources during a project. Early in the research collaboration, ECRs should emphasize the potential for, and importance of, nontraditional career metrics that may differ from the home institution’s promotion metrics but are normalized in the D&I field (e.g., publishing methodological approaches early in studies, developing dissemination products to operational partners) and could expand the reach of the study’s findings [12]. An additional challenge is the time, effort, and cost involved in collaborative D&I research approaches, particularly in instances in which essential tasks (e.g., relationship building, hiring staff with unique expertise, ensuring fair and just compensation for partners, promoting the willingness of the team to engage in collaboration throughout the project) may have implications for researchers’ promotion and tenure. As a field, more work is needed to incentivize implementation manuscripts – while findings from successful clinical trials may be published in high-impact journals, findings that inform implementation, sustainability or scalability of an efficacious program or intervention may have a broader societal impact. However, individual ECRs may need to weigh their institution’s internal promotion metrics against the value of nontraditional publications and transdisciplinary research as they contemplate collaborations within and outside of their institution [31]. Our recommendations prompt the ECR to be explicit about the challenges and potential benefits of implementation-focused projects and products with principal investigators, research teams, and mentors.

### Training and mentorship are critical throughout D&I collaborations

We identified several considerations for the field regarding the preparation of ECRs as D&I collaborators. First, there was some debate within our study sample about the importance of formal D&I training given the nascency of the field; however, as the field expands, literature proliferates, and demand for expertise increases, it is likely that credentialed learning opportunities (e.g., certificate programs) will become more essential to collaboration success [10]. Formal training opportunities that span disciplines are becoming more widespread – a 2023 review identified 165 D&I capacity-building programs, with activities ranging from providing resources/tools and webinars to coursework and formal mentorship [10]. From a health equity perspective, our study participants noted that D&I training may be less accessible to under-represented ECRs and that senior mentors may be hesitant to collaborate with ECRs from different backgrounds. It is important that the field (a) more explicitly acknowledge the complexities of equity in D&I training [31], (b) ensure that equity is incorporated into training expectations for ECRs, and (c) focus on expanding access to D&I mentoring for under-represented ECRs, including offering more training in cultural competence for senior mentors as well as investing in efforts to recruit under-represented D&I experts to faculty positions.

An ECR’s career development and future success can be enhanced by interactions with traditional mentors as well as sponsors and people connected to various relevant networks. A particularly salient finding in our study that aligns with prior work [12,32] was the importance of access to mentors with established D&I and non-D&I networks who could provide opportunities, offer methodological insight, act as a champion, and serve as resources should the ECR have collaboration or scientific questions. Study participants viewed such mentors as crucial to ECRs’ success in meeting key career benchmarks. As Tabak et al. note, having a network can lend credibility to an ECR who has less experience [6]; however, given the nascency of the field, there are increasingly few “experienced” D&I researchers to serve as a mentor for ECRs. Peer mentoring units, such as the one from which this paper was developed, can help fill training and knowledge gaps for ECRs if more senior mentorship is unavailable, as long as roles and responsibilities are shared across the group [33]. Peer mentoring units can also offer an avenue for establishing new interdisciplinary collaborations. As noted by Luke et al, peers participating in a structured D&I training program reported new projects, grants, and writing collaborations [11]. Our work can serve to guide discussions among ECRs and both their D&I and non-D&I mentors because it incorporates recommendations regarding points in one’s career at which additional mentorship may be needed, how to access mentors (e.g., through training programs), types of mentorships which may be helpful, and areas in which a mentor can provide specific guidance.

### ECRs should consider how health equity and D&I science intersect in collaborations

Throughout our study, participants described the importance of incorporating health equity into our recommendations. Participants suggested that a health equity-informed D&I ECR should be able to articulate ways in which D&I research approaches can advance health equity; demonstrate familiarity with applying health equity frameworks to D&I; and describe their experiences of having collaborated and/or engaged with diverse team members and collaborators. Although progress at the intersection of health equity research and D&I science is continuously evolving, we offer suggestions to support ECR in this regard: First, ECRs with only a fundamental knowledge of health equity science or scholarship should seek opportunities to partner with and co-learn from individuals who have acquired health equity expertise and/or individuals with lived experience of health inequities and/or systematic discrimination, rather than participating in “health equity tourism” [34]. ECRs should seek to learn from the vast historical literature in health equity, and stay abreast of more current literature from experts who intersect health equity and D&I science in order to establish guiding principles for their collaborative research (e.g., framework selection and instrument development and adaptation). Second, ECRs should practice reflexivity – acknowledging and articulating their individual and professional equity-related strengths and limitations, and how those strengths and limitations may influence their role in a given study or within a given discipline. For example, an ECR who is very familiar with organizational metrics developed for D&I may still be unequipped to systematically adapt those metrics for use within historically minoritized populations. Third, in considering whether to collaborate on a given study, D&I ECRs should consider the study principal investigator and research team’s stated commitment to addressing inequities (e.g., budget for equity consultant, time built in for relationships-building), familiarize themselves with the role and reputation of the organization(s) involved in the project within the community, and encourage community engagement and co-learning from the outset of the study [35,36].

### Limitations and strengths

Our study has several limitations and strengths which should be acknowledged. First, our study sample is small and homogenous (sample primarily self-identified as cis-women and/or white). Future efforts are needed to refine recommendations and establish competencies through a broader set of perspectives, including both researchers and practitioners belonging to marginalized groups, in various other settings in which D&I work is conducted (e.g., community locations, nonacademic health care systems), and in other geographical locations (USA, Europe, Australia, Latin America, Africa). Second, we recruited through convenience and snowball sampling in our networks, thus some individuals may have participated in both surveys, which may have limited the number of participants or the reach of this study. Third, although we made efforts to decrease the amount of time it took to complete the survey, about 40% of individuals who started the second survey did not complete it, which may have been due to the number of text-heavy and open-ended questions.

Despite these limitations, our study has several strengths: First, during our study conceptualization, data collection, analysis, and interpretation, we leveraged the expertise of many D&I researchers and practitioners of different career lengths who represented various practice locations and backgrounds and diverse areas of content and expertise. Second, the domains and recommendations were iteratively developed across several phases, which enabled us to discuss and refine the domains and recommendations. Third, we sought and obtained feedback from a diverse group of individuals who engage in D&I projects and studies, including ECR mentees and senior D&I research mentors, who provided valuable insights into career levels for D&I work.

## Conclusion

Productive, interdisciplinary research collaborations are essential to advance clinical and translational research. ECRs trained in D&I may require additional support to enhance their collaborative research practice, but the need for D&I-specific collaboration competencies remains open for consideration. Overall, our list of domains and recommendations can assist ECRs in promoting and leveraging their diverse skillset within D&I and non-D&I-focused projects. Future work is needed to integrate these recommendations as key competencies in fundamental D&I trainings as well as for the D&I mentoring workforce. Findings lay the groundwork for future studies to expand recommendations (e.g., by eliciting practitioners’ perspectives) and empirically assess the extent to which the recommendations impact both the quality and quantity of D&I ECRs’ scientific collaborations.

## Supporting information

Lane et al. supplementary material 1 Lane et al. supplementary material

Lane et al. supplementary material 2 Lane et al. supplementary material

Lane et al. supplementary material 3 Lane et al. supplementary material

## Supplementary material

The supplementary material for this article can be found at https://doi.org/10.1017/cts.2024.684.

## References

[1] “Dissemination & Implementation (D&I) Research.” Office of Disease Prevention. (https://prevention.nih.gov/research-priorities/dissemination-implementation) Accessed 16 Aug. 2023.

[2] What Is D&I? | Dissemination & Implementation Research | Washington University in St. Louis. (https://implementationresearch.wustl.edu/what-is-d-and-i/) Accessed 16 Aug. 2023.

[3] Wuchty S , Jones BF , Uzzi B. The increasing dominance of teams in production of knowledge. Science. 2007;316(5827):1036–1039. 17431139 10.1126/science.1136099

[4] Aarons GA , Reeder K , Miller CJ , Stadnick NA. Identifying strategies to promote team science in dissemination and implementation research. J Clin Transl Sci. 2020;4(3):180–187. 10.1017/cts.2019.413 PMC7348006 32695486

[5] Hall KL , Olster DH , Stipelman BA , Vogel AL. News from NIH: resources for team-based research to more effectively address complex public health problems. Transl Behav Med. 2012;2(4):373–375. 24073137 10.1007/s13142-012-0172-1 PMC3717922

[6] Tabak RG , Bauman AA , Holtrop JS. Roles dissemination and implementation scientists can play in supporting research teams. Implement Sci Commun. 2021;2(1):9. 33451364 10.1186/s43058-020-00107-4 PMC7811259

[7] Chambers DA , Proctor EK , Brownson RC , Straus SE. Mapping training needs for dissemination and implementation research: lessons from a synthesis of existing D&I research training programs. Transl Behav Med. 2017;7(3):593–601. 27030472 10.1007/s13142-016-0399-3 PMC5645270

[8] Schultes MT , Aijaz M , Klug J , Fixsen DL. Competences for implementation science: what trainees need to learn and where they learn it. Adv Health Sci Educ Theory Pract. 2021;26(1):19–35. 32372393 10.1007/s10459-020-09969-8 PMC7900055

[9] Brownson RC , Colditz GA , Dobbins M , Emmons KM , Kerner JF , Padek M , et al. Concocting that Magic Elixir: Successful Grant Application Writing in Dissemination and Implementation Research. Clin Transl Sci. 2015;8(6):710–716. 26577630 10.1111/cts.12356 PMC4739635

[10] Viglione C , Stadnick NA , Birenbaum B , et al. A systematic review of dissemination and implementation science capacity building programs around the globe. Implement Sci Commun. 2023;4(1):34. 36973832 10.1186/s43058-023-00405-7 PMC10041476

[11] Luke DA , Baumann AA , Carothers BJ , Landsverk J , Proctor EK. Forging a link between mentoring and collaboration: a new training model for implementation science. Implement Sci. 2016;11(1):137. 27737693 10.1186/s13012-016-0499-y PMC5062835

[12] Stamatakis KA , Norton WE , Stirman SW , Melvin C , Brownson RC. Developing the next generation of dissemination and implementation researchers: insights from initial trainees. Implement Sci. 2013;8:29. 23497462 10.1186/1748-5908-8-29 PMC3626831

[13] Koorts H , Naylor PJ , Laws R , Love P , Maple JL , van Nassau F. What hinders and helps academics to conduct dissemination and implementation (D&I) research in the field of nutrition and physical activity? An international perspective. Int J Behav Nutr Phys Act. 2020;17(1):7. 31948456 10.1186/s12966-020-0909-z PMC6966833

[14] Guerrero EG , Hahn EE , Khachikian T , Chuang E , Brown AF. Interdisciplinary dissemination and implementation research to advance translational science: challenges and opportunities. J Clin Transl Sci. 2017;1(1):67–72. 28480057 10.1017/cts.2016.4 PMC5408838

[15] Lewinski AA , Sullivan C , Allen KD , Crowley MJ , Gierisch JM , Goldstein KM , et al. Accelerating implementation of virtual care in an integrated health care system: Future research and operations priorities. J Gen Intern Med. 2021;36(8):2434–2442. 33496928 10.1007/s11606-020-06517-3 PMC8342733

[16] Powell BJ , Waltz TJ , Chinman MJ , et al. A refined compilation of implementation strategies: results from the expert recommendations for implementing change (ERIC) project. Implement Sci. 2015;10(1):21. 25889199 10.1186/s13012-015-0209-1 PMC4328074

[17] Hull L , Goulding L , Khadjesari Z , et al. Designing high-quality implementation research: development, application, feasibility and preliminary evaluation of the implementation science research development (ImpRes) tool and guide. Implement Sci. 2019;14(1):80. 31412887 10.1186/s13012-019-0897-z PMC6693182

[18] Forrest CB , Chesley FD Jr. , Tregear ML , Mistry KB. Development of the learning health system researcher core competencies. Health Serv Res. 2018;53(4):2615–2632. 28777456 10.1111/1475-6773.12751 PMC6051975

[19] Waltz TJ , Powell BJ , Fernández ME , Abadie B , Damschroder LJ. Choosing implementation strategies to address contextual barriers: diversity in recommendations and future directions. Implement Sci. 2019;14(1):42. 31036028 10.1186/s13012-019-0892-4 PMC6489173

[20] Lewinski AA , Crowley MJ , Miller C , et al. Applied rapid qualitative analysis to develop a contextually appropriate intervention and increase the likelihood of uptake. Med Care. 2021;59:S242–S251. 33976073 10.1097/MLR.0000000000001553 PMC8132894

[21] Lewinski AA-O , Shapiro A , Bosworth HB , Crowley MJ , McCant F , Howard T , et al. Veterans’ Interpretation of Diabetes Distress in Diabetes Self-Management: Findings From Cognitive Interviews. Sci Diabetes Self Manage Care. 2021;47(5):391–403. 10.1177/26350106211043487 PMC9494356 34559032

[22] Gonzales SA , Okusaga O , Reuteman-Fowler J , Oakes M , Brown J , Moore S , et al. Digital medicine system in veterans with severe mental illness: Feasibility and acceptability study. JMIR Form Res. 2022;6(12):e34893. 36548028 10.2196/34893 PMC9816955

[23] Blooms Taxonomy: Resource for Educators. (https://bloomstaxonomy.net/) Accessed 7 July 2023.

[24] Gonzales R , Handley MA , Ackerman S , OʼSullivan PS . A framework for training health professionals in implementation and dissemination science. Acad Med. 2012;87(3):271–278. 22373617 10.1097/ACM.0b013e3182449d33 PMC3307591

[25] Padek M , Mir N , Jacob RR , et al. Training scholars in dissemination and implementation research for cancer prevention and control: a mentored approach. Implement Sci. 2018;13(1):18. 29357876 10.1186/s13012-018-0711-3 PMC5778694

[26] Shea CM , Young TL , Powell BJ , Rohweder C , Enga ZK , Scott JE , et al. Researcher readiness for participating in community-engaged dissemination and implementation research: A conceptual framework of core competencies. Transl Behav Med. 2017;7(3):393–404. 28341897 10.1007/s13142-017-0486-0 PMC5645278

[27] Metz A , Albers B , Burke K , et al. Implementation practice in human service systems: understanding the principles and competencies of professionals who support implementation. Hum Serv Organ Manag Leadersh Gov. 2021;45(3):238–259.

[28] Tabak RG , Padek MM , Kerner JF , et al. Dissemination and implementation science training needs: insights from practitioners and researchers. Am J Prev Med. 2017;52(3 Suppl 3):S322–S329. 28215389 10.1016/j.amepre.2016.10.005 PMC5321656

[29] Mallidou AA , Atherton P , Chan L , Frisch N , Glegg S , Scarrow G. Core knowledge translation competencies: a scoping review. Bmc Health Serv Res. 2018;18(1):502. 29945609 10.1186/s12913-018-3314-4 PMC6020388

[30] Straus SE , Brouwers M , Johnson D , et al. Core competencies in the science and practice of knowledge translation: description of a Canadian strategic training initiative. Implement Sci. 2011;6:127. 22152223 10.1186/1748-5908-6-127 PMC3292943

[31] Maddox BB , Phan ML , Byeon YV , et al. Metrics to evaluate implementation scientists in the USA: what matters most? Implement Sci Commun. 2022;3(1):75. 35842690 10.1186/s43058-022-00323-0 PMC9287698

[32] Jacob RR , Gacad A , Pfund C , et al. The, secret sauce, for a mentored training program: qualitative perspectives of trainees in implementation research for cancer control. Bmc Med Educ. 2020;20(1):237. 32723326 10.1186/s12909-020-02153-x PMC7385963

[33] Dickson KS , Glass JE , Barnett ML , Graham AK , Powell BJ , Stadnick NA. Value of peer mentoring for early career professional, research, and personal development: a case study of implementation scientists. J Clin Transl Sci. 2021;5(1):e112. 34221454 10.1017/cts.2021.776 PMC8223172

[34] Lett E , Adekunle D , McMurray P , et al. Health equity tourism: ravaging the justice landscape. J Med Syst. 2022;46(3):17. 35150324 10.1007/s10916-022-01803-5 PMC8853313

[35] Baumann AA , Long PD. Equity in implementation science is long overdue (SSIR). (https://ssir.org/articles/entry/equity_in_implementation_science_is_long_overdue) Accessed 17 July 2023.

[36] Kerkhoff AD , Farrand E , Marquez C , Cattamanchi A , Handley MA. Addressing health disparities through implementation science—a need to integrate an equity lens from the outset. Implement Sci. 2022;17(1):13. 35101088 10.1186/s13012-022-01189-5 PMC8802460

