# Supplementary material for: Recommendations for interdisciplinary research collaboration for early career dissemination and implementation researchers: A multi-phase study
Source: J Clin Transl Sci. 2025 Jan 17;9(1):e39. doi: 10.1017/cts.2024.684 (PMC11883575; doi:10.1017/cts.2024.684)
Supplement: Lane et al. supplementary material 1 — Lane et al. supplementary material [file S2059866124006848sup001.docx]

**I. Domains Presented in Phase 2**

*Marketing Oneself As a D&I Researcher:*

- **Describing specific D&I training**  (e.g., listing formal didactic and experiential training in D&I methods; listing organizational memberships; describing content of D&I courses and workshops)
- **Describing specific D&I knowledge**  (e.g., indicating knowledge of determinant and outcome frameworks, study designs, analytic techniques, implementation processes and strategies, fidelity vs adaptation, spread, scale, sustainability, etc; demonstrating knowledge by listing relevant publications and/or studies)
- **Articulating your D&I research experience.**  (e.g., highlighting experience as a methodologist and/or within a content area; demonstrating familiarity with relevant journals and seminal works; describing experience collecting and managing D&I data, applying D&I knowledge to areas beyond content expertise.
- **Describing transferable skills** (e.g., articulating how one's skills and experiences apply to D&I such as: evaluation, qualitative methods, mixed methods studies, community-engaged research, health economics, non-randomized trials)

*Considerations when approached about a new D&I collaboration*

- **Getting to know the team dynamic**  (e.g., ask about collaborator expectations, leadership style, team track record; meet with other Co-Is; determine alignment of the team's working style with your preferences)
- **Determining the PI's commitment to D&I**  (e.g., review preliminary data and the rationale for D&I; know what stage the project is in and how much D&I is valued; assess baseline D&I expertise of PI and your role in educating them)
- **Defining your role**  (e.g., negotiate appropriate effort for your career stage and amount of work required; discuss line items needed for D&I beyond your effort are included in the budget; make sure you are comfortable with deadlines and expectations)
- **Ensuring the value to your career**  (e.g., discuss and document the opportunities for lead and co-authorship; consider how project supports your future independent research/promotion; ask how PI and team will support your growth, provide mentorship, continue collaboration)

*Responsibilities of a D&I collaborator once the project is initiated*

- **Setting expectations about D&I research**  (e.g., discuss time and resources required, as well as value-added; be realistic about scope within timeline and budgets; determine consistency of existing data/measures with D&I aims; align scope of D&I with funding mechanism)
- **Providing technical expertise**  (e.g., provide appropriate references; employ planning tools such as PRECIS or behavior change wheel to revise aims; when possible, integrate D&I content and methods across all grant sections and study phases)
- **Managing the budget**  (e.g., cost out line items required for D&I portion of the study; overlap line items with other aims where possible; stick to your budget
- **Working well with others**  (e.g., oversee collection and management of implementation data; attend group meetings to offer a D&I perspective; stick to your timeline and budget; share resources such as staff

II**. Domains Presented in Phase 3**

*Marketing oneself as a D&I researcher*

- **D&I training and knowledge: documented evidence of formal or informal training/knowledge related to D&I**
- Describe formal didactic and experiential training in D&I methods, including content of courses and workshops
- Demonstrate familiarity with the resources of the field (e.g., organizational memberships, relevant journals and seminal works)
- Demonstrate access to mentorship from D&I experts
- **Applied experience: relevant experiences from both within and outside of the D&I**
- Distinguish D&I skills from other research skills gained while working on research projects
- Highlight application of discrete D&I concepts through relevant research dissemination products
- Describe specialization in a specific content, method, context, or setting in D&I
- Describe relevance of non-D&I research and practice experience to D&I projects
- **Stakeholder collaboration: experience ability partnering and communicating with different types of partners**
- Describe experiences with non-academic stakeholders
- Demonstrate ability to communicate with both academic and non-academic stakeholders
- Describe experiences with inter-disciplinary research teams

*Key considerations when approached about D&I collaboration*

- **The project’s D&I potential: feasibility and acceptability of the proposed D&I component**
  - Evaluate feasibility of conducting D&I component using planned or existing methods, resources, and partnerships
  - Determine acceptability and understanding of D&I among PI and other research team members
  - Negotiate expectations and resources needed for the on D&I component
- **Time, effort and your toolbox: role and responsibilities of the D&I collaborator on the project**
  - Ensure alignment between your working style and collaboration expectations
  - Assess whether the resources and mentorship/expertise at your disposal are adequate to carry out project
  - Ensure alignment between your content, methods and/or setting/context expertise and what is needed for the project
  - Negotiate team leadership roles appropriate for career stage and planned effort
- **Career goals and metrics: alignment with your short and long term career goals**
  - Evaluate how the project aligns with the early career researcher’s goals
  - Evaluate how the PI and team support the early career researcher’s goals
  - Document alignment of the project plan with career advancement/promotion and tenure metrics

*Responsibilities of a D&I collaborator once project is initiated*

- **Resource stewardship: Managing project resources wisely**
  - Recognize key points for project decision-making related to D&I goals
  - Adapt resource commitments to changing project demands
  - Maintain open communication with PI in regards to resource and project management
- **Providing technical expertise**
  - Strategically engage in project activities to advocate for D&I goals
  - Apply D&I principles and methods to project activities and demands
  - Adapt D&I vocabulary to meet needs of project stakeholders
- **Being a good steward to the D&I field**
  - Continually advance your own D&I learning
  - Identify and engage in opportunities for professional growth

III**. Domains Presented During Phase 4**

*Marketing/Self-Promotion as a D&I Researcher*

- **Evidence of D&I knowledge, skills, and attitudes**
  - Identify key skills and attributes gained from formal didactic training in D&I and relevance to a range of research questions
  - Articulate the ways in which D&I research approaches can advance health equity
  - Describe how you will leverage your networks and resources to fill gaps in your D&I knowledge, skills, and attitudes
  - Demonstrate awareness of, and engagement with, ongoing initiatives to advance the D&I field
- **Applied experience: relevant experiences from both within and outside of the D&I**
  - Detail specific experiences on D&I projects
  - Describe relevance of non-D&I research and practice experience to D&I projects
- **Stakeholder collaboration: skills and experience partnering and communicating with different types of partners**
  - Describe experiences collaborating and engaging with diverse stakeholders
  - Demonstrate ability to communicate with diverse stakeholders
  - Describe experiences working as part of inter-disciplinary teams

*Key considerations when approached about D&I collaboration (Project specific)*

- **The project’s D&I potential: feasibility and acceptability of the proposed D&I component**
  - Evaluate feasibility of conducting D&I component using validated methods within proposed budget, resources, and partnerships
  - Assess D&I knowledge, skills, and attitude among PI and other research team members
  - Define expectations and resources and expertise needed for the D&I component
- **Time, effort and your toolbox: role and responsibilities of the D&I collaborator on the project**
  - Determine alignment between project team timelines and commitment expectations with your available time and effort
  - Justify how project scope ‘fits’ your content, methods and/or setting expertise
  - Justify appropriateness of proposed role for career stage, and allocated effort
  - Assess if organizational resources and mentorship/expertise at your disposal are adequate to carry out project
- **Career goals and metrics: alignment with your short- and long-term career goals**
  - Appraise alignment between the proposed project and early career researcher (ECR) short and long-term research and career goals and portfolio
  - Appraise how the project team members can support short and long-term research and career goals

*Responsibilities of a D&I collaborator once project is initiated*

- **Communication and project management: Cultivating and sustaining an environment for stakeholder-driven D&I science**
- Employ open communication with project team and stakeholders regarding project resource management and modifications to the D&I research plan
- Use shared project decision-making to revise plans across the project lifespan
- Assess and iteratively revise engagement and roles of stakeholders across the project lifespan
- **Technical Expertise: Applying technical skills and knowledge to carry out research aims**
  - Plan, employ, and revise project activities as needed to advance D&I project aims
  - Explain project activities and findings using minimal jargon throughout the project
- **Scholarly development: being a good steward to yourself and the D&I field**
  - Evaluate and report the extent to which project methods and results advance the D&I field of research
  - Demonstrate ability to leverage project for professional growth within the D&I field

**IV. Detailed Results and Decision Process Across Phases 2-3**

**Area 1: Marketing yourself**

| **Phase 2** | | | | | | | |
| --- | --- | --- | --- | --- | --- | --- | --- |
| Initial Domains | Survey Results^a^ (n=41)  N(%) | | | Open-Ended Response Key Findings^b^ | Actions Taken | Focus Group Findings (n=12) | Actions Taken |
|  | NI | MI | VI |  |  |  |  |
| *Describing specific D&I training* | 2 (5%) | 17 (42%) | 22 (54%) | - Didactic training appears to hold less importance than other topics, perhaps because current training opportunities are nascent. | Explore in FGs: differing perspectives of ECR vs non-ECR. | - Training is important for ECRs who have had access to it, but applied work is equally or more important. Expecting ECRs to have formal training is unjustified and ageist. | Combine with knowledge and refine based on feedback |
| *Describing specific D&I knowledge* | 2 (5%) | 8 (20%) | 31 (76%) | - Add knowledge of implementation to the definition. - Key missing topic: Communication, “knowing the lingo” | Explore in FGs: Communication across research cycle | - Key recommendation: know contextually relevant jargon but avoid D&I jargon since D&I collaborators often communicate with both research team and operation partners | Combine with training and refine based on feedback |
| *Articulating your D&I research experience*^c^ | 2 (5%) | 6 (15%) | 32 (78%) | - Distinguish methods, content, and context experience. - Show “evidence of your expertise” - Should “not conflate” course-work with experience, or agree to projects outside scope of experience - Key missing topics: having access to D&I resources and mentors; existing partners; ability to communicate with partners | Explore in FGs: specificity needed in describing experience (e.g., generalized vs focused); defining productive partnerships and mentors; acceptable “evidence of expertise” for ECRs | - No consensus about marketing as a generalist vs “niche” specialist, but agreement that collaborations should align with your identity. - Key recommendations: Access mentors with “social capital” and experience across lifespan of a research project; demonstrate that you have worked with and can communicate with operational partners. | Refine based on feedback; combine with Describing Transferable Skills |
| *Describing transferable skills* | 2 (5%) | 11 (27%) | 28 (68%) | - Considerable overlap with competencies from adjacent fields - Key missing topics: describing value-add of D&I to operation partners and study team; articulating resource needs; access to mentors and experts from D&I and adjacent fields | Explore in FGs: Communication across research cycle; access to mentors | - Make sure recommendations are related to D&I and not confused with adjacent fields | Combined with Articulating experience |
| *Other Domains* |  |  |  |  |  |  | Added stakeholder collaboration domain |

^a^NI=Not that important; MI=Moderately Important; VI=Very important

^b^Responses to prompt: “What, in your opinion, is most important for early career researchers to keep in mind when marketing their knowledge, skills and abilities and seeking D&I collaborations? What knowledge, experiences and applied methods are not reflected in the dimensions?”

^c^n=40-41

| **Phase 3** | | | | |
| --- | --- | --- | --- | --- |
| Activities to Inform Recommendations | Survey Results^a^ | | | Actions Taken for Recommendations |
|  | Needs Edits (#;%) | Total # Comments^b^ | Summary of Comments |  |
| ***Evidence of D&I knowledge, skills, and attitudes (n=24 responses)*** | | | | |
| Describe formal didactic and experiential training in D&I methods, including content of courses and workshops^c^ | 5 (21%) | Clarity: 3  Content: 1  No Comment: 1 | Clarity: Alter/simplify wording; use learning outcome language (e.g., Bloom’s taxonomy)  Content: Include context and sponsor of training | Used learning outcome language  Added to guidance in table |
| Demonstrate familiarity with the resources of the field (e.g., organizational memberships, relevant journals and seminal works) | 3 (13%) | Clarity: 1  Content: 1  No Comment: 1 | Clarity: Add specificity  Content: Include workgroups and initiatives | Used learning outcome language  Added to guidance in table |
| Demonstrate access to mentorship from D&I experts | 6 (25%) | Clarity: 3  Content: 2  No Comment: 1 | Clarity: Use learning outcome language; provide definitions  Content: Field lacks guidance on qualifications of a mentor/expert | Used learning outcome language |
| Missing recommendations identified |  |  | None | -- |
| ***Applied experience: relevant experiences from both within and outside of the D&I field (n=23 responses)*** | | | | |
| Distinguish D&I skills from other research skills gained while working on research projects | 3 (13%) | Clarity: 2  Content: 1 | Clarity: Provide definitions  Content: Unsure if we want to promote this “silo-ing” | Condensed two recommendations [see below]  Used learning outcomes language  Added to guidance in table |
| Highlight application of discrete D&I concepts through relevant research dissemination products | 5 (22%) | Clarity: 5 | Clarity: Provide definitions; use learning outcome language; alter/simplify wording | Condensed two recommendations [see above]  Used learning outcomes language  Added to guidance in table |
| Describe specialization in a specific content, method, context, or setting in D&I | 4 (17%) | Clarity: 2  Content: 2 | Clarity: Add specificity, provide definitions  Content: Unsure if “specialism” is helpful or creates more silos | Minor wording changes  Added to guidance in table |
| Describe relevance of non-D&I research and practice experience to D&I projects^c^ | 1 (5%) | Content: 1 | Content: Consider the overlap and gray areas (e.g., improvement science) | Minor wording changes  Added to guidance in table |
| Missing recomendations identified |  |  | - Equity learning outcome - Knowledge of D&I international landscape | - Engaged equity consultant in order to incorporate equity focus throughout - Addressed in Discussion section |
| ***Stakeholder collaboration: skills and experience partnering and communicating with different types of partners (n=21 responses)*** | | | | |
| Describe experiences with non-academic stakeholders | 1 (5%) | Clarity: 1 | Clarity: Alter/simplify wording | Minor wording changes  Added to guidance in table |
| Demonstrate ability to communicate with both academic and non-academic stakeholders | 3 (14%) | Clarity: 2  No Comment: 1 | Clarity: Alter/simplify wording | Minor wording changes  Added to guidance in table |
| Describe experiences with inter-disciplinary research teams | 1 (5%) | Clarity: 1 | Clarity: Add specificity | Added to guidance in table |
| Missing recommendations identified |  |  | - Describe experience in roles of researcher, consultant, and trainer | Added to guidance in table |

^a^Respondents were randomized to receive different domains first to ensure feedback on all. Results reflect respondents who answered questions in this section

^b^Clarity=comment related to modifications to the wording or clarification of meaning; Content=comment included content related to application of the recommendation

^c^n=22

**Area 2: Key considerations when approached about D&I collaboration**

| **Phase 2** | | | | | | | |
| --- | --- | --- | --- | --- | --- | --- | --- |
| Initial Domains | Survey Results^a^ (n=41) | | | Open-Ended Response Key Findings^b^ | Actions Taken | Focus Group Findings (n=12) | Actions Taken |
|  | NI | MI | VI |  |  |  |  |
| *Getting to know the team dynamic* | 1 (2%) | 15 (37%) | 25 (61%) | - Overlap with principles of collaboration/team science so perhaps not necessary. | Explore in FG: identify any D&I specific recommendations separate that don’t overlap with team science/ collaboration | - Unique D&I team dynamic recommendations may relate to having larger projects with more diverse partners and unique metrics and outcomes - Having a more senior mentor may help to “elevate” the role of D&I on project - Reiterated importance of communication across research life cycles | Eliminated due to overlap with other disciplines |
| *Determining the PI's commitment to D&I* | 1 (2%) | 20 (49%) | 20 (49%) | - Considerable overlap between this topic and “Defining your role” - Educating PI and study team about the need for/role of D&I can be a heavy lift - Key new topic: Consider if project is actually D&I or just “purported” to be | Change topic: determine potential integration of D&I in project rather than specific focus on the PI | -- | Refined based on feedback to focus on the project |
| *Defining your role* | 0 (0%) | 1 (2%) | 40 (98%) | - Overlap between this topic and “Determining PI’s commitment to D&I” - Key new topics: Ensure time and effort are compensated and that role of ECR is clarified, importance of flexibility in working with the team | Change topic: determine potential D&I landscape of the project rather than specific focus on the PI | - Key recommendation: identify where in D&I continuum project falls (e.g., implementation vs dissemination) and how that aligns with your career goals - Need to write a scope of work to avoid “traps” (e.g., engaging in data collection or intervention, being seen as a team member rather than a leader) - Expectation management is key because D&I research is a lot of work. Do D&I goals align with broader project goals? | Refined based on feedback |
| *Ensuring the value to your career* | 0 (0%) | 10 (24%) | 31 (76%) | - Key new topics: how the project could enable learning new skills, content areas, or contexts; plan for publications throughout a project | Explore in FG: unique academic products in D&I | - Key recommendation: describe unique products that D&I scientists produce, product needs of operational partners | Refined based on feedback |
| *Other Domains* |  |  |  |  |  |  | None |

^a^NI=Not that important; MI=Moderately Important; VI=Very important

^b^Responses to prompt: “What, in your opinion, is most important for early career researchers to keep in mind when marketing their knowledge, skills and abilities and seeking D&I collaborations? What knowledge, experiences and applied methods are not reflected in the dimensions?”

| **Phase 3** | | | | |
| --- | --- | --- | --- | --- |
| Activities to Inform Recommendations | Survey Results^a^ | | | Actions Taken |
|  | Needs Edits (#;%) | Comments (#)^b^ | Summary of Comments |  |
| ***The project’s D&I potential: feasibility and acceptability of the proposed D&I component (n=24 responses)*** | | | | |
| Evaluate feasibility of conducting D&I component using planned or existing methods, resources, and partnerships | 4 (17%) | Clarity: 2  No Comment: 1 | Clarity: Add specificity | Minor wording changes  Added to guidance in table |
| Determine acceptability and understanding of D&I among PI and other research team members | 1 (4%) | Content:1 | Content: Knowing PI’s skills/training more important than acceptability | Changed content  Added to guidance in table |
| Negotiate expectations and resources needed for the on D&I component^c^ | 5 (22%) | Clarity: 2  No Comment: 3 | Clarity: Alter/simplify wording; add specificity | Added to guidance in table |
| Missing recommendation identified |  |  | - Identify key problem and potential alternative solutions | Added to guidance in table |
| ***Time, effort and your toolbox: role and responsibilities of the D&I collaborator on the project (n-22)*** | | | | |
| Ensure alignment between your working style and collaboration expectations^d^ | 4 (19%) | Clarity: 2  No Comment: 2 | Clarity: Alter/simplify wording, provide definitions | Minor content and wording changes  Added to guidance in table |
| Assess whether the resources and mentorship/ expertise at your disposal are adequate to carry out project | 0 (0%) |  |  | Used learning outcomes language |
| Ensure alignment between your content, methods and/or setting/context expertise and what is needed for the project | 1 (5%) | Clarity: 1 | Clarity: Alter/simplify wording | Minor wording changes  Added to guidance in table |
| Negotiate team leadership roles appropriate for career stage and planned effort | 1 (5%) | Content: 1 | Content: Less about career stage and more about what one brings. Advocate for oneself if expectations are beyond one’s abilities | Minor content and wording changes  Added to guidance in table |
| Missing recommendation identified |  |  | - Access to data - Clarification of role and alignment with title (e.g., Co-I vs consultant) - Ensure formal commitment from “peer group” | Added to guidance in table |
| ***Career goals and metrics: alignment with your short- and long-term career goals (n=22)*** | | | | |
| Evaluate how the project aligns with the ECR’s goals | 1 (5%) | Content: 1 | Content: Builds foundation for future work | Minor wording changes  Added to guidance in table |
| Evaluate how the PI and team support ECR’s goals | 1 (5%) | Clarity: 1 | Clarity: Alter/simplify wording | Minor wording changes  Added to guidance in table |
| Document alignment of the project plan with career advancement/promotion and tenure metrics | 2 (9%) | Clarity: 1  Content: 1 | Clarity: Use use learning outcome language (e.g., Bloom’s taxonomy)  Content: Use Translational Science Benefit Model to articulate how ECRs can convey unique contribution | Eliminated and included in Table Added to guidance in table |
| Missing recommendation identified |  |  | None |  |

^a^Respondents were randomized to receive different domains first to ensure feedback on all. Results reflect respondents who answered questions in this section

^b^Clarity=comment related to modifications to the wording or clarification of meaning; Content=comment included content related to application of the recommendation

^c^n=23

^d^n=22

**Area 3: Responsibilities of a D&I collaborator once project is initiated**

| **Phase 1** | **Phase 2** | | | | | | |
| --- | --- | --- | --- | --- | --- | --- | --- |
| Initial Domains | Survey Results^a^ (n=41) | | | Open-Ended Response Key Findings^b^ | Actions Taken | Focus Group Findings (n=12) | Actions Taken |
|  | NI | MI | VI |  |  |  |  |
| *Setting expectations about D&I research* | 1 (2%) | 10 (24%) | 30 (73%) | - Considerable overlap between this topic and others - Need tangible examples | Explore in FG: tangible ways to establish expectations | - Frequently check in on roles and responsibilities, continue expectation management, and ensure effort evolves to reflect extra time and effort needed - Should allow flexibility to do what fits in with the expertise you carve out for yourself as your career (and the D&I field) evolves, as long as that still aligns with what you proposed - Should happen prior to project start | Absorbed within “key considerations” category |
| *Providing technical expertise^c^* | 0 (0%) | 13 (32%) | 27 (66%) | - Important for any project, not just D&I - Articulating D&I expertise may be more relevant prior to securing funding - Need continued access to/consultation with more senior D&I experts | Explore in FG: access to mentors; D&I stewardship | - Debate about how much exposure to practice (e.g., data collection, programming) were necessary but agreement that it should be established a priori and re-visited throughout the project. | Refined and expanded based on feedback |
| *Managing the budget^c^* | 4 (10%) | 29 (68%) | 8 (20%) | - Primarily the role of study PI | Change topic: managing project resources wisely | -- | Refined based on feedback to focus on overall resource management |
| *Working well with others* | 2 (5%) | 5 (12%) | 33 (81%) | - Similar to “team dynamic” in Collaboration Considerations, overlap with principles of collaboration/team science so perhaps not necessary. - Key new topic: communication across research cycle | Explore in FG: communication across research cycles; specific team dynamic recommendations for D&I | -- | Eliminated due to overlap with other disciplines |
| *Other Domains* |  |  |  | - Key new topic: seeking out opportunities to advance on learning and educate others about D&I | Explore new topic in FG; | ECRs should plan to mentor given the “shallow” D&I bench. Caution against too much responsibility on ECRs—should be good ambassadors outside the field and seek influence in smaller spheres, but also balance self-develop with field-development | Added D&I field stewardship domain |

^a^NI=Not that important; MI=Moderately Important; VI=Very important

^b^Responses to prompt: “What, in your opinion, is most important for early career researchers to keep in mind when marketing their knowledge, skills and abilities and seeking D&I collaborations? What knowledge, experiences and applied methods are not reflected in the dimensions?”

^c^n=40

| **Phase 3** | | | | |
| --- | --- | --- | --- | --- |
| Activities to Inform Recommendations | Survey Results | | | Actions Taken |
|  | Needs Edits (#;%) | Comments (#)^b^ | Summary of Comments |  |
| ***Resource stewardship: managing project resources wisely (n=23 responses)*** | | | | |
| Recognize key points for project decision-making related to D&I goals | 4 (17%) | Clarity: 4 | Clarity: Add specificity | Condensed two recommendations [see below] and minor wording changes Added to guidance in table |
| Adapt resource commitments to changing project demands | 5 (22%) | Clarity: 3  Content: 2 | Clarity: Redundant with another recommendation; Alter/simplify wording  Content: Need broad base of methods for if “real-world” projects do not go as planned | Condensed two recommendations [see above] and minor wording changes  Added to guidance in table |
| Maintain open communication with PI in regards to resource and project management | 2 (9%) | Clarity: 2  Content: 1 | Clarity: Alter/simplify wording  Content: Include communication with external stakeholders | Minor wording changes  Added to guidance in table |
| Missing recommendations identified |  |  | - Negotiate/manage resources and time needed for stakeholder involvement across research cycle - Time management/ commitment - Communication with external stakeholders | Added to guidance in table  Added communication recommendation |
| ***Technical expertise: strategically advancing D&I project aims (n=23 responses)*** | | | | |
| Strategically engage in project activities to advocate for D&I goals | 5 (22%) | Clarity: 5 | Clarity: Add specificity; Provide definition; Alter/ simplify wording | Minor wording changes  Added to guidance in table |
| Apply D&I principles and methods to project activities and demands | 1 (4%) | Clarity: 1 | Clarity: Add specificity | Eliminated and included as guidance |
| Adapt D&I vocabulary to meet needs of project stakeholders^c^ | 2 (9%) | Clarity: 2 | Clarity: Provide definition | Eliminated and included as guidance |
| Missing recommendations identified |  |  | - Science communication - Advocacy/”people skills” - Networking, especially across fields | Added communication recommendation  Added to guidance in table |
| ***Scholarly development: being a good steward to yourself and the D&I field (n=23 responses)*** | | | | |
| Continually advance your own D&I learning | 1 (4%) | Content: 1 | Content: Knowing where and how to advance learning gaps in D&I | Minor content and wording changes  Added to guidance in table |
| Identify and engage in opportunities for professional growth. | 1 (4%) | Clarity: 1 | Clarity: Use learning outcomes language | Eliminated and included in Table Column 3 |
| Leverage project experiences to advance the D&I discipline within your sphere of influence | 3 (13%) | Clarity: 3 | Clarity: Add specificity; provide definition | Used learning outcomes language  Added to guidance in table |
| Missing recommendation identified |  |  | - Join expertise networks - Support growth of others | Added to guidance in table |

^a^Respondents were randomized to receive different domains first to ensure feedback on all. Results reflect respondents who answered questions in this section

^b^Clarity=comment related to modifications to the wording or clarification of meaning; Content=comment included content related to application of the recommendation

^c^n=22

**IV. Summary of Expert Input in Phase 4**

**Summary of Additional Expert Input**

In general, expert input led to further refinement and rearrangement of recommendations and guidance across areas, including the provision of references to better align with the existing literature. Based on prior findings, expert input focused on two primary topics: mentors/partnerships, and equity.

**Mentors, Partnerships, Resources.** Phase 2 findings suggested the need to more specifically define “mentorship” in D&I across the research areas. Experts noted the importance of having both D&I methods-oriented mentors as well as mentors in your substantive discipline. Experts also noted that D&I mentors may or may not help facilitate partner meetings or networking opportunities, and ECRs may need to rely on other sponsors for that. and to identify experts who will set up partner meetings and help ECRs build a network. Experts felt that a key recommendation for an ECR would be identifying areas where they would need mentorship on a project, and to then seek out those mentors. They emphasized the importance of also serving as a mentor to research staff, students, or other investigators. In terms of partnerships, experts emphasized the value of the perspectives of the ultimate beneficiaries (e.g., patients, community members), and ensuring that they are interested in what you are studying. Engaging partners takes time because of the trust required, and that should be accounted for in funding applications. Experts noted the importance of compensating partners for all tasks—attending meetings, reviewing materials, any consultation, etc. Experts made several recommendations related to communication. They noted the importance of face-to-face meetings for building relationships and sharing results. They also emphasized the need for a multi-pronged dissemination approach, including tailoring presentations and visualizations for various audiences across study phases.. We also inquired about resources that are needed to carry out a D&I study. Experts emphasized the importance of well-trained staff, and suggested engaging those who “do the work” in budget and resource planning to understand the amount of time required for a given task or set of tasks. Staff effort and workflows should be deliberately re-visited throughout the project through regularly scheduled meetings between staff and investigators

**Equity.** When reviewing recommendations with attention to health equity, the authorship team (led by AB) identified key areas where recommendation language and guidance should be modified. Additionally, in general, we identified gaps in our analysis that should be explore further in future work.

- **Marketing/Promotion:** In terms of didactic training, concerns were raised about the limited availability, expense, and needed connection with a university associated with these trainings. While online trainings exist to increase knowledge, they are insufficient for connecting with mentors and building collaboration skills. As a related point, an expectation that ECRs be active members in organizations may introduce inequities due to the associated time and financial costs. Involvement in organizations may also be difficult without mentors or sponsors providing access and support. Another key point is that while knowledge of frameworks that intersect equity and implementation science as well as equity-informed methods related to instrument development, recruitment, planning, data collection/analysis, etc., they are also relatively new and have limited empirical backing. Thus, ECRs should be aware of emerging literature in this area but not necessarily skilled or experienced. Finally, ECR D&I experts should avoid “equity tourism;” rather, they should read learn from and co-create with peers who are equity experts, and collaborate equitably with practitioners and other partners. In terms of marketing/promotion one’s experience with stakeholder collaboration, an ECR should be as specific about how partner relationships were forged, how partners were engaged and the extent to which engagement was maintained through the project.
- **Collaboration Considerations**: In terms of the project’s D&I potential, it is important to understand the limitations of many measures and methods in D&I that focus at the organizational level, and the extent to which those measures address the positive and negative impact those organizations have had within their community—to the extent possible, take time to familiarize oneself with those roots. Also, acknowledge that traditional D&I methods often do not incorporate the “people’s” perspective, that is, those who are impacted by changes in this organization. In terms of the project team, self-reflection of behavior and composition is an important consideration, as well as the team’s stated commitment to address equity gaps. For example, have they budgeted for consultation with equity experts, and time for relationship-building? Do they have agreements and compensation plans in place for partners?
