# Supplementary material for: Recommendations for interdisciplinary research collaboration for early career dissemination and implementation researchers: A multi-phase study
Source: J Clin Transl Sci. 2025 Jan 17;9(1):e39. doi: 10.1017/cts.2024.684 (PMC11883575; doi:10.1017/cts.2024.684)
Supplement: Lane et al. supplementary material 2 — Lane et al. supplementary material [file S2059866124006848sup002.docx]

Phase 1 Survey Items

How would you describe your race (select all that apply)?

- American Indian or Alaska Native
- Asian
- Black or African American
- Native Hawaiian or other Pacific Islander
- White
- Other [please specify:] ____________________________________
- Don’t Know
- Prefer not to say

How would you describe your ethnicity (select all that apply)?

- Hispanic or Latino
- Not Hispanic or Latino
- Prefer not to say

How would you describe your gender identity (select all that apply)?

- Cis-Woman
- Cis-Man
- Trans woman
- Trans man
- Gender queer/ Gender non-conforming/ Questioning
- Another identity [please specify]: ______________________________________
- Prefer not to answer

Please indicate the extent of your agreement with this statement: "I consider myself a D&I researcher."

- Strongly agree
- Somewhat agree
- Neither agree nor disagree
- Somewhat disagree
- Strongly disagree

Please briefly (< 150 words) describe why you consider yourself a D&I researcher: ________________________________________________________________

________________________________________________________________

Which of the following describes your training/experience (select all that apply)?

- I have served as PI on a grant or project
- I have served as PI on a D&I grant or project
- I am an early career researcher (within 10 years of terminal degree or postdoc completion)
- I have training or experience with D&I
- I have an interest in D&I, but no formal D&I experience or training.

Which of the following describes your collaboration and mentoring experience (select all that apply)?

- I have formally or informally mentored early career researchers.
- I have formally or informally mentored early career researchers related to D&I
- I have served as Co-I or collaborator on a grant or project
- I have served as Co-I or collaborator on a D&I study or project

Please briefly (< 150 words) describe your discipline of training, broad topic/content area of interest, and current work setting: ________________________________________________________________

___________________________________________________________________________________

-------------------------------------------------------------------------------------------------------------------------------

Our goal with this modified Delphi survey is to refine topics related to collaboration which have been identified by a group of early career D&I researchers. In the second phase, we will elicit feedback on practical "competencies" within topics, that can serve as guidance for early career D&I researchers engaging in collaboration.

This survey consists of three sections that are organized by topic. For each section, you will first be asked to rate the importance of various dimensions associated with that topic. Then, you will be asked for feedback on how to refine the description of those dimensions or to identify additional dimensions that should be covered. When responding, draw on your experience as a mentor, collaborator, and/or early career researcher, particularly as related to D&I.

-------------------------------------------------------------------------------------------------------------------------------

**Topic 1: Key marketable knowledge, skills, and abilities of D&I researchers**

This topic refers to how early career D&I scientists can most effectively convey their knowledge, skills, and abilities in their CVs, biosketches, faculty pages, at conferences and networking events, etc., as these documents and events may lead to their inclusion as D&I experts on grants and projects.

Consider these four dimensions of **key marketable knowledge, skills, and abilities of D&I researchers.** Potential competencies, which will be refined in the second phase, are listed in parentheses. How important are these dimensions for early career researchers to convey themselves as D&I scientists?

|  | Not that important | Moderately important | Very important |
| --- | --- | --- | --- |
| **Describing specific D&I training**  (e.g., listing formal didactic and experiential training in D&I methods; listing organizational memberships; describing content of D&I courses and workshops) |  |  |  |
| **Describing specific D&I knowledge**  (e.g., indicating knowledge of determinant and outcome frameworks, study designs, analytic techniques, implementation processes and strategies, fidelity vs adaptation, spread, scale, sustainability, etc; demonstrating knowledge by listing relevant publications and/or studies) |  |  |  |
| **Articulating your D&I research experience.**  (e.g., highlighting experience as a methodologist and/or within a content area; demonstrating familiarity with relevant journals and seminal works; describing experience collecting and managing D&I data, applying D&I knowledge to areas beyond content expertise. |  |  |  |
| **Describing transferable skills** (e.g., articulating how one's skills and expereinces apply to D&I such as: evaluation, qualitative methods, mixed methods studies, community-engaged research, health economics, non-randomized trials) |  |  |  |

What, in your opinion, is most important for early career researchers to keep in mind when marketing their knowledge, skills and abilities and seeking D&I collaborations? What is **not** reflected in the dimensions above?

________________________________________________________________

________________________________________________________________

**Topic 2: Considerations when approached about a new D&I collaboration**

This topic refers to a number of considerations for early career scientists to contemplate when they are approached about serving as a D&I collaborator or co-investigator on a project or study.
  
Consider these four dimensions of **considerations when approached about a new D&I collaboration.** Potential competencies, which will be refined in the second phase, are listed in parentheses. How important are these dimensions for early career D&I researchers as they consider collaborating on new projects?

|  | Not that important | Moderately important | Very important |
| --- | --- | --- | --- |
| **Getting to know the team dynamic**  (e.g., ask about collaborator expectations, leadership style, team track record; meet with other Co-Is; determine alignment of the team's working style with your preferences) |  |  |  |
| **Determining the PI's commitment to D&I**  (e.g., review preliminary data and the rationale for D&I; know what stage the project is in and how much D&I is valued; assess baseline D&I expertise of PI and your role in educating them) |  |  |  |
| **Defining your role**  (e.g., negotiate appropriate effort for your career stage and amount of work required; discuss line items needed for D&I beyond your effort are included in the budget; make sure you are comfortable with deadlines and expectations) |  |  |  |
| **Ensuring the value to your career**  (e.g., discuss and document the opportunities for lead and co-authorship; consider how project could support your future independent research or promotion; ask how PI and team will support your growth, provide mentorship, and continue collaboration) |  |  |  |

What, in your opinion, is most important for early career researchers to keep in mind when approached about a new D&I collaboration? What is not reflected in the dimensions above?

________________________________________________________________

________________________________________________________________

**Topic 3: Responsibilities of a D&I collaborator**

 This topic refers to a number of responsibilities that early career D&I scientists should be prepared for in terms of writing grants and executing projects once collaboration has been agreed about, regardless of where in the overall study/project timeline that collaboration ensues.

Consider these four dimensions of **responsibilities of a D&I collaborator** once the project is initiated. Potential competencies, which will be refined in the second phase, are listed in parentheses.

|  | Not that important | Moderately important | Very important |
| --- | --- | --- | --- |
| **Setting expectations about D&I research**  (e.g., discuss time and resources required, as well as value-added; be realistic about scope within timeline and budgets; determine consistency of existing data/measures with D&I aims; align scope of D&I with funding mechanism) |  |  |  |
| **Providing technical expertise**  (e.g., provide appropriate references; employ planning tools such as PRECIS or behavior change wheel to revise aims; when possible, integrate D&I content and methods across all grant sections and study phases) |  |  |  |
| **Managing the budget**  (e.g., cost out line items required for D&I portion of the study; overlap line items with other aims where possible; stick to your budget |  |  |  |
| **Working well with others**  (e.g., oversee collection and management of implementation data; attend group meetings to offer a D&I perspective; stick to your timeline and budget; share resources such as staff |  |  |  |

What, in your opinion, is most important for early career researchers to keep in mind when serving as a D&I collaborator on a new project? What is not reflected in the dimensions above?_

______________________________________________________________

________________________________________________________________

-------------------------------------------------------------------------------------------------------------------------------

Now that you have reviewed all three topic areas, what other important guidance for ECR researchers engaging in D&I collaboration has not been captured?

________________________________________________________________

________________________________________________________________

**Phase 3 Survey Items**

We are interested in obtaining perspectives from D&I researchers. Do you fall into one of these categories?

- I am an early career D&I researcher (within 10 years of terminal degree or postdoc completion)
- I have served as a consultant on a funded D&I grant or project
- I have served as co-I on a funded D&I grant or project
- I have served as PI on a funded D&I grant or project
- Yes
- No

How would you describe your race (select all that apply)?

- American Indian or Alaska Native
- Asian
- Black or African American
- Native Hawaiian or other Pacific Islander
- White
- Other [please specify:] ____________________________________
- Don’t Know
- Prefer not to say

How would you describe your ethnicity (select all that apply)?

- Hispanic or Latino
- Not Hispanic or Latino
- Prefer not to say

How would you describe your gender identity (select all that apply)?

- Cis-Woman
- Cis-Man
- Transwoman
- Transman
- Gender queer/ Gender non-conforming/ Questioning
- Another identity [please specify]: ______________________________________
- Prefer not to answer

Please indicate the extent of your agreement with this statement: "I consider myself a D&I researcher."

- Strongly agree
- Somewhat agree
- Neither agree nor disagree
- Somewhat disagree
- Strongly disagree

Which of the following describes your training/experience (select all that apply)?

- I am an early career D&I researcher (within 10 years of terminal degree or postdoc completion)
- I have served as a consultant on a funded D&I grant or project
- I have served as Co-I on a funded D&I grant or project
- I have served as PI on a funded D&I grant or project

Which best describes your training discipline? (select all that apply)

- Medicine
- Nursing
- Psychology/Sociology
- Public Health/Population Health
- Business/Economics
- Education
- Other (please specify): _________________________________________________

Which of the following describes your mentoring experience (select all that apply)?

- I have formally or informally mentored early career researchers.
- I have formally or informally mentored early career researchers related to D&I
- Not applicable as I am an early career researcher

Please briefly (< 150 words) describe your sub-specialization in D&I: ________________________________________________________________

________________________________________________________________

Please select where your work falls on the continuum of applied to theoretical D&I

| **Applied** | **Theoretical** |
| --- | --- |


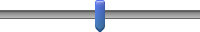


Our prior research (which included survey, focus groups, and brain storming sessions with Early Career Researchers) identified potential D&I collaboration competencies in 3 topical areas. 

**Topic 1.** Marketing/promoting oneself as a D&I researcher
**Topic 2.** Key considerations when approached about D&I collaboration
**Topic 3.**Responsibilities of a D&I collaborator once project is initiated

The rest of the survey will ask for your feedback on each of these topical areas. The order in which these topics are presented will vary to minimize bias based on the content of other topical areas.

-------------------------------------------------------------------------------------------------------------------------------

The information below is an overview of Topic 1. This information will be presented at the top of each page for your reference.

**Topic 1: Marketing/promoting oneself as a D&I researcher**  
This topic refers to competencies for effectively conveying D&I related knowledge, skills, and abilities in the early career researcher’s CV, Biosketch, faculty pages, and conferences and networking events. The purpose of marketing/promotion activities is to demonstrate the early career investigator’s suitability for inclusion as a D&I expert on grants and projects.

Competencies for this topic were organized into 3 domains:

- Domain 1. D&I training and knowledge: documented evidence of formal or informal training and knowledge related to D&I
- Domain 2. Applied experience: relevant experiences from both within and outside of the D&I field
- Domain 3. Stakeholder collaboration: skills and experience partnering and communicating with different types of partners.

**Domain 1. D&I training and knowledge: documenting evidence of formal or informal training and knowledge related to D&I.**
Please review the competencies below. For each, indicate if you recommend changes and if so, describe what is needed to provide additional detail or clarity to the competency

*Competency 1. Describe formal didactic and experiential training in D&I methods, including content of courses and workshops.*

Do you suggest changes for this competency?

- Yes
- No

[If yes] Please describe areas of confusion or suggest alternative wording for this competency: ________

*Competency 2. Demonstrate familiarity with the resources of the field (e.g., organizational memberships, relevant journals and seminal works)*
Do you suggest changes for this competency?

- Yes
- No

[If yes] Please describe areas of confusion or suggest alternative wording for this competency: ________

*Competency 3. Demonstrate access to mentorship from D&I experts*
Do you suggest changes for this competency?

- Yes
- No

[If yes] Please describe areas of confusion or suggest alternative wording for this competency: ________

What, if any, essential competencies are missing and should be included in this domain? Please provide a rationale for including these: ____________________________________________________________

_____________________________________________________________________________________

**Domain 2. Applied experience: relevant experiences from both within and outside of the D&I field**
Please review the competencies below. For each, indicate if you recommend changes and if so, describe what is needed to provide additional detail or clarity to the competency.

*Competency 1. Distinguish D&I skills from other research skills gained while working on research projects*

Do you suggest changes for this competency?

- Yes
- No

[If yes] Please describe areas of confusion or suggest alternative wording for this competency: ________

*Competency 2. Highlight application of discrete D&I concepts through relevant research dissemination products*
Do you suggest changes for this competency?

- Yes
- No

[If yes] Please describe areas of confusion or suggest alternative wording for this competency: ________

*Competency 3. Describe specialization in a specific content, method, context, or setting in D&I* 
 
Do you suggest changes for this competency?

- Yes
- No

[If yes] Please describe areas of confusion or suggest alternative wording for this competency: ________

*Competency 4. Describe relevance of non-D&I research and practice experience to D&I projects*
 
Do you suggest changes for this competency?

- Yes
- No

[If yes] Please describe areas of confusion or suggest alternative wording for this competency: ________

What, if any, essential competencies are missing and should be included in this domain? Please provide a rationale for including these: ____________________________________________________________

_____________________________________________________________________________________

**Domain 3. Stakeholder collaboration: skills and experience partnering and communicating with different types of partners.**
Please review the competencies for this domain below. For each, indicate if you recommend changes and if so, describe what is needed to provide additional detail or clarity to the competency.

*Competency 1. Describe experiences with non-academic stakeholders* 
Do you suggest changes for this competency?

- Yes
- No

[If yes] Please describe areas of confusion or suggest alternative wording for this competency: _______

*Competency 2. Demonstrate ability to communicate with both academic and non-academic stakeholders*
 
Do you suggest changes for this competency?

- Yes
- No

[If yes] Please describe areas of confusion or suggest alternative wording for this competency: ________ _______________________________________________________________________\

*Competency 3. Describe experiences with inter-disciplinary research teams*Do you suggest changes for this competency?

- Yes
- No

[If yes] Please describe areas of confusion or suggest alternative wording for this competency: ________

What, if any, essential competencies are missing and should be included in this domain? Please provide a rationale for including these: ____________________________________________________________

________________________________________________________________________________________________________________________________________________________________________

-------------------------------------------------------------------------------------------------------------------------------

The information below is an overview of Topic 2. This information will be presented at the top of each page for your reference.

**Topic 2: Key considerations when approached about D&I collaboration**

This topic refers to competencies for a number of essential project features that early career researchers should to contemplate when they are approached about serving as a D&I collaborator (i.e., consultant or co-I) or co-investigator on a project or study.
 
Competencies for this topic were organized into 3 domains:

- Domain 1. The project’s D&I potential: feasibility and acceptability of the proposed D&I component
- Domain 2. Time, effort and your toolbox: role and responsibilities of the D&I collaborator on the project
- Domain 3. Career goals and metrics: alignment with your short- and long-term career goals

**Domain 1. The project’s D&I potential: feasibility and acceptability of the proposed D&I component**
Please review the competencies for this domain below. For each, indicate if you recommend changes and if so, describe what is needed to provide additional detail or clarity to the competency.

*Competency 1. Evaluate feasibility of conducting D&I component using planned or existing methods, resources, and partnerships*
 
Do you suggest changes for this competency?

- Yes
- No

[If yes] Please describe areas of confusion or suggest alternative wording for this competency: ________

*Competency 2. Determine acceptability and understanding of D&I among PI and other research team members*

Do you suggest changes for this competency?

- Yes
- No

[If yes] Please describe areas of confusion or suggest alternative wording for this competency: ________

*Competency 3. Negotiate expectations and resources needed for the on D&I component*
 
Do you suggest changes for this competency?

- Yes
- No

[If yes] Please describe areas of confusion or suggest alternative wording for this competency: ________

What, if any, essential competencies are missing and should be included in this domain? Please provide a rationale for including these: __________________________________________________________________________________________________________________________________

**Domain 2. Time, effort and your toolbox: role and responsibilities of the D&I collaborator on the project**
Please review the competencies for this domain below. For each, indicate if you recommend changes and if so, describe what is needed to provide additional detail or clarity to the competency.

*Competency 1.Ensure alignment between your working style and collaboration expectations*Do you suggest changes for this competency?

- Yes
- No

[If yes] Please describe areas of confusion or suggest alternative wording for this competency: ________

*Competency 2. Assess whether the resources and mentorship/expertise at your disposal are adequate to carry out project*

Do you suggest changes for this competency?

- Yes
- No

[If yes] Please describe areas of confusion or suggest alternative wording for this competency: ________

*Competency 3. Ensure alignment between your content, methods and/or setting/context expertise and what is needed for the project* 
Do you suggest changes for this competency?

- Yes
- No

[If yes] Please describe areas of confusion or suggest alternative wording for this competency:_______

*Competency 4. Negotiate team leadership roles appropriate for career stage and planned effort*
 
Do you suggest changes for this competency?

- Yes
- No

[If yes] Please describe areas of confusion or suggest alternative wording for this competency:_______

What, if any, essential competencies are missing and should be included in this domain? Please provide a rationale for including these: ____________________________________________________________

_____________________________________________________________________________________

**Domain 3. Career goals and metrics: alignment with your short- and long-term career goals**
Please review the competencies for this domain below. For each, indicate if you recommend changes and if so, describe what is needed to provide additional detail or clarity to the competency.

*Competency 1. Evaluate how the project aligns with the early career researcher’s goals*

Do you suggest changes for this competency?

- Yes
- No

[If yes] Please describe areas of confusion or suggest alternative wording for this competency:_______

*Competency 2. Evaluate how the PI and team support the early career researcher’s goals*

Do you suggest changes for this competency?

- Yes
- No

If yes] Please describe areas of confusion or suggest alternative wording for this competency:_______

*Competency 3. Document alignment of the project plan with career advancement/promotion and tenure metrics*

Do you suggest changes for this competency?

- Yes
- No

[If yes] Please describe areas of confusion or suggest alternative wording for this competency:_______

What, if any, essential competencies are missing and should be included in this domain? Please provide a rationale for including these: ____________________________________________________________

_____________________________________________________________________________________

-------------------------------------------------------------------------------------------------------------------------------

The information below is an overview of Topic 3. This information will be presented at the top of each page for your reference.

**Topic 3: Responsibilities of a D&I collaborator once project is initiated**
 
This topic refers to competencies for a number of responsibilities that early career researchers should be prepared for in terms of writing D&I grants and executing D&I projects once collaboration has been agreed upon, regardless of where in the overall study/project timeline that collaboration ensues.

Competencies for this topic were organized into 3 domains:

- Domain 1. Resource stewardship: managing project resources wisely
- Domain 2. Technical expertise: strategically advancing D&I project aims
  Domain 3. Scholarly development: being a good steward to yourself and the D&I field

**Domain 1. Resource stewardship: managing project resources wisely**
Please review the competencies for this domain below. For each, indicate if you recommend changes and if so, describe what is needed to provide additional detail or clarity to the competency.

*Competency 1. Recognize key points for project decision-making related to D&I goals*

Do you suggest changes for this competency?

- Yes
- No

[If yes] Please describe areas of confusion or suggest alternative wording for this competency:_______

*Competency 2. Adapt resource commitments to changing project demands*

Do you suggest changes for this competency?

- Yes
- No

[If yes] Please describe areas of confusion or suggest alternative wording for this competency:_______

*Competency 3. Maintain open communication with PI in regards to resource and project management*

Do you suggest changes for this competency?

- Yes
- No

[If yes] Please describe areas of confusion or suggest alternative wording for this competency:_______

___________________________________________________________________________________

What, if any, essential competencies are missing and should be included in this domain? Please provide a rationale for including these: ____________________________________________________________

_____________________________________________________________________________________

**Domain 2. Technical expertise: strategically advancing D&I project aims**
Please review the competencies for this domain below. For each, indicate if you recommend changes and if so, describe what is needed to provide additional detail or clarity to the competency.

*Competency 1. Strategically engage in project activities to advocate for D&I goals*

Do you suggest changes for this competency?

- Yes
- No

[If yes] Please describe areas of confusion or suggest alternative wording for this competency:_______

*Competency 2. Apply D&I principles and methods to project activities and demands*

Do you suggest changes for this competency?

- Yes
- No

[If yes] Please describe areas of confusion or suggest alternative wording for this competency:_______

*Competency 3. Adapt D&I vocabulary to meet needs of project stakeholders*

 Do you suggest changes for this competency?

- Yes
- No

[If yes] Please describe areas of confusion or suggest alternative wording for this competency:_______

What, if any, essential competencies are missing and should be included in this domain? Please provide a rationale for including these: ____________________________________________________________

_____________________________________________________________________________________

**Domain 3. Scholarly development: being a good steward to yourself and the D&I field**
Please review the competencies for this domain below. For each, indicate if you recommend changes and if so, describe what is needed to provide additional detail or clarity to the competency.

*Competency 1. Continually advance your own D&I learning*Do you suggest changes for this competency?

- Yes
- No

[If yes] Please describe areas of confusion or suggest alternative wording for this competency:_______

*Competency 2. Identify and engage in opportunities for professional growth*

Do you suggest changes for this competency?

- Yes
- No

[If yes] Please describe areas of confusion or suggest alternative wording for this competency:_______

*Competency 3. Leverage project experiences to advance the D&I discipline within your sphere of influence*

Do you suggest changes for this competency?

- Yes
- No
- [If yes] Please describe areas of confusion or suggest alternative wording for this competency:_______

What, if any, essential competencies are missing and should be included in this domain? Please provide a rationale for including these: _____________________________________________________________________________________________________________________________________

-------------------------------------------------------------------------------------------------------------------------------

**Final Thoughts**

If you would like to provide any final comments or thoughts on the 3 topics and their affiliated domains and competencies, please share here:

__________________________________________________________________________________________________________________________________________________________________________
