# Supplementary material for: Recommendations for interdisciplinary research collaboration for early career dissemination and implementation researchers: A multi-phase study
Source: J Clin Transl Sci. 2025 Jan 17;9(1):e39. doi: 10.1017/cts.2024.684 (PMC11883575; doi:10.1017/cts.2024.684)
Supplement: Lane et al. supplementary material 3 — Lane et al. supplementary material [file S2059866124006848sup003.pptx]

## Slide 1
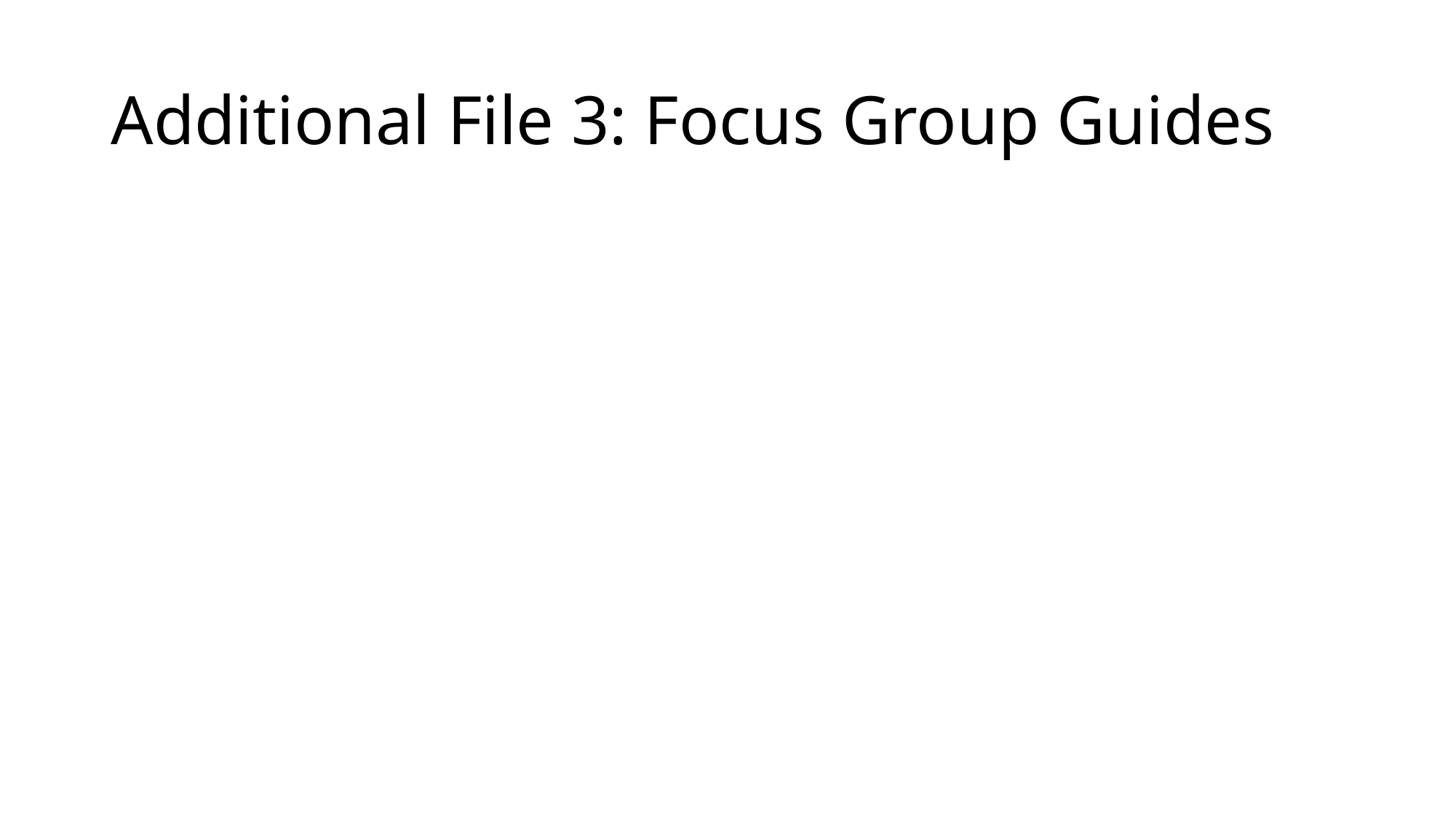

# Additional File 3: Focus Group Guides

## Slide 2
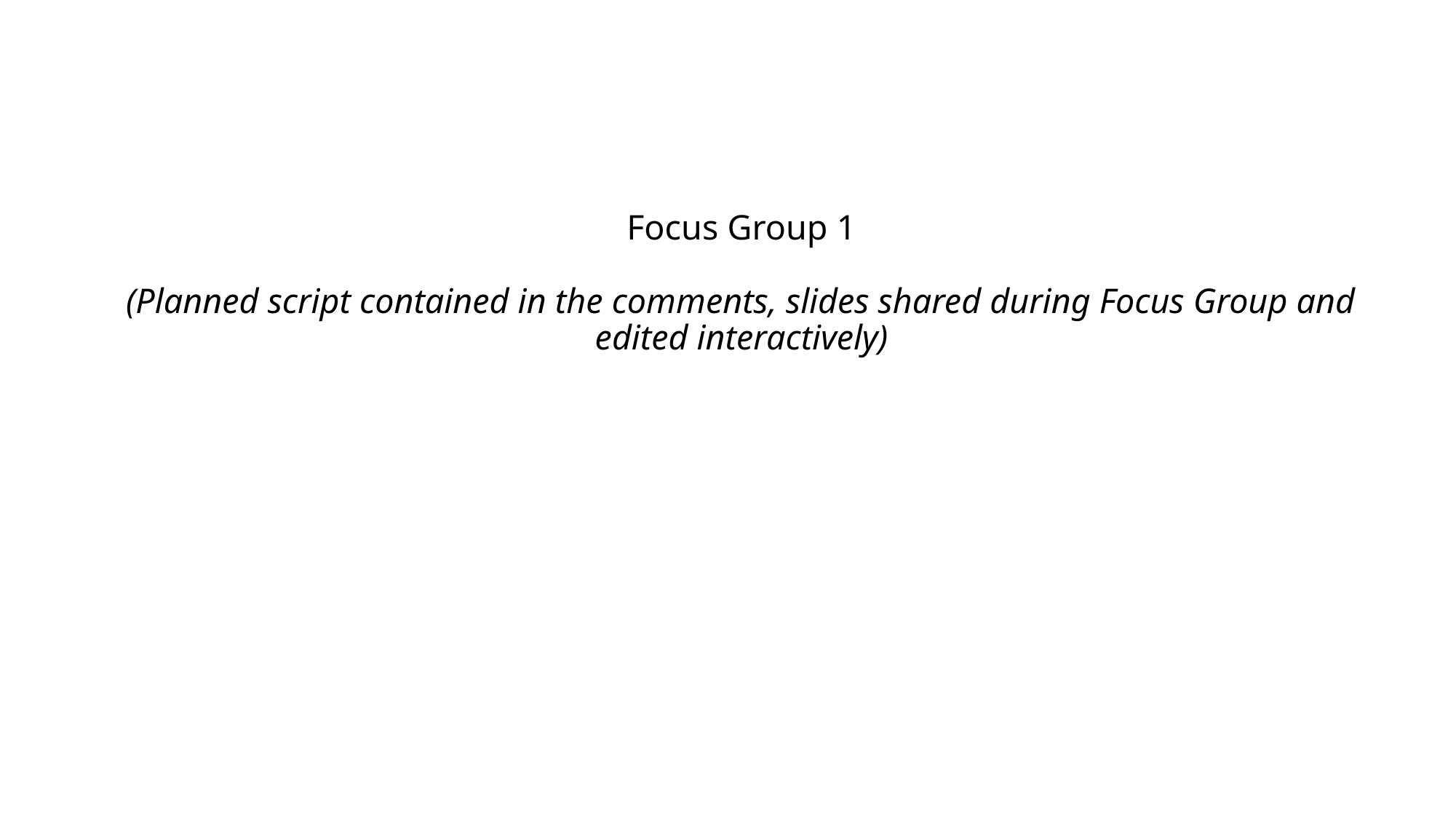

# Focus Group 1 (Planned script contained in the comments, slides shared during Focus Group and edited interactively)

## Slide 3
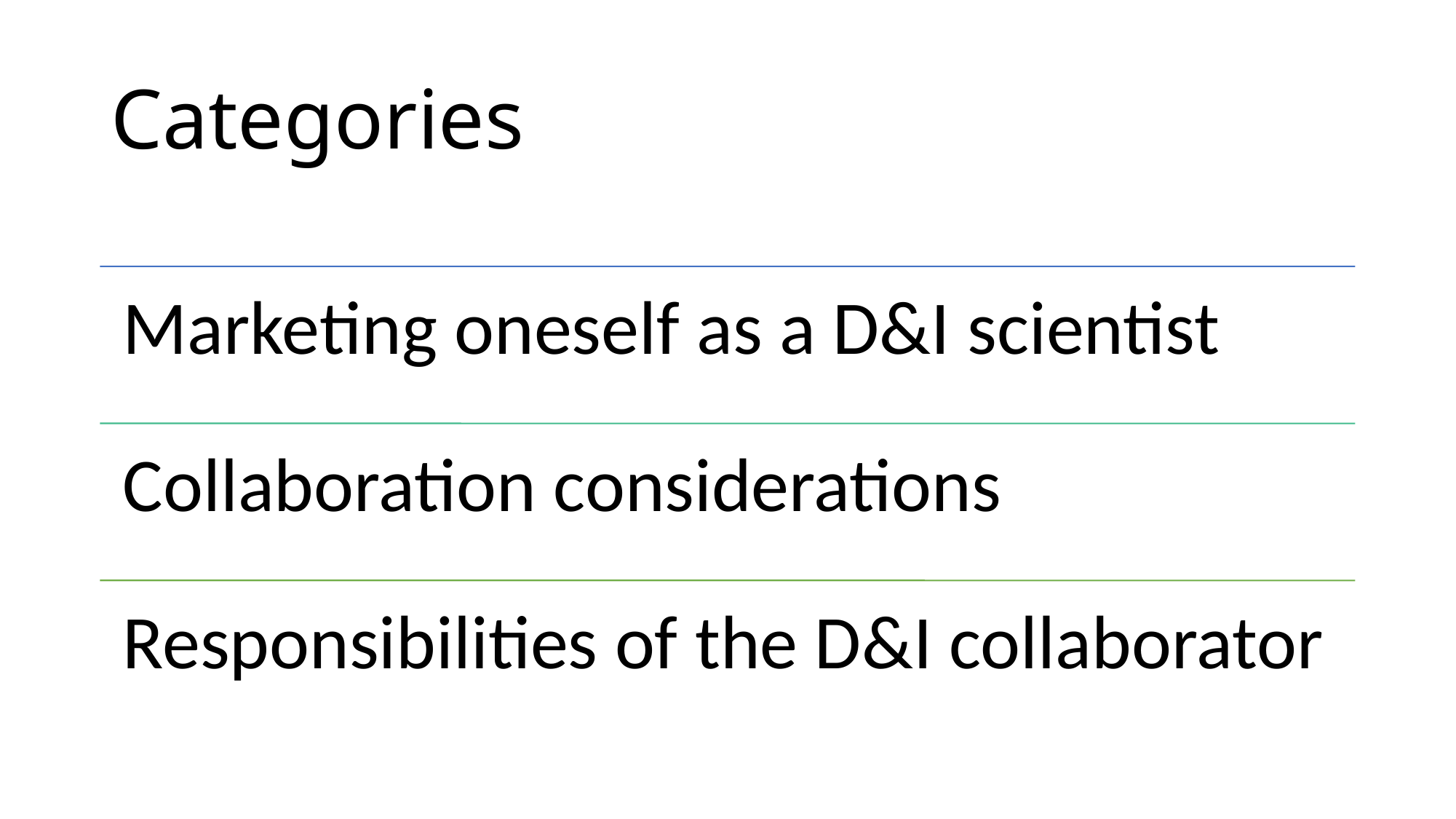

# Categories

## Slide 4
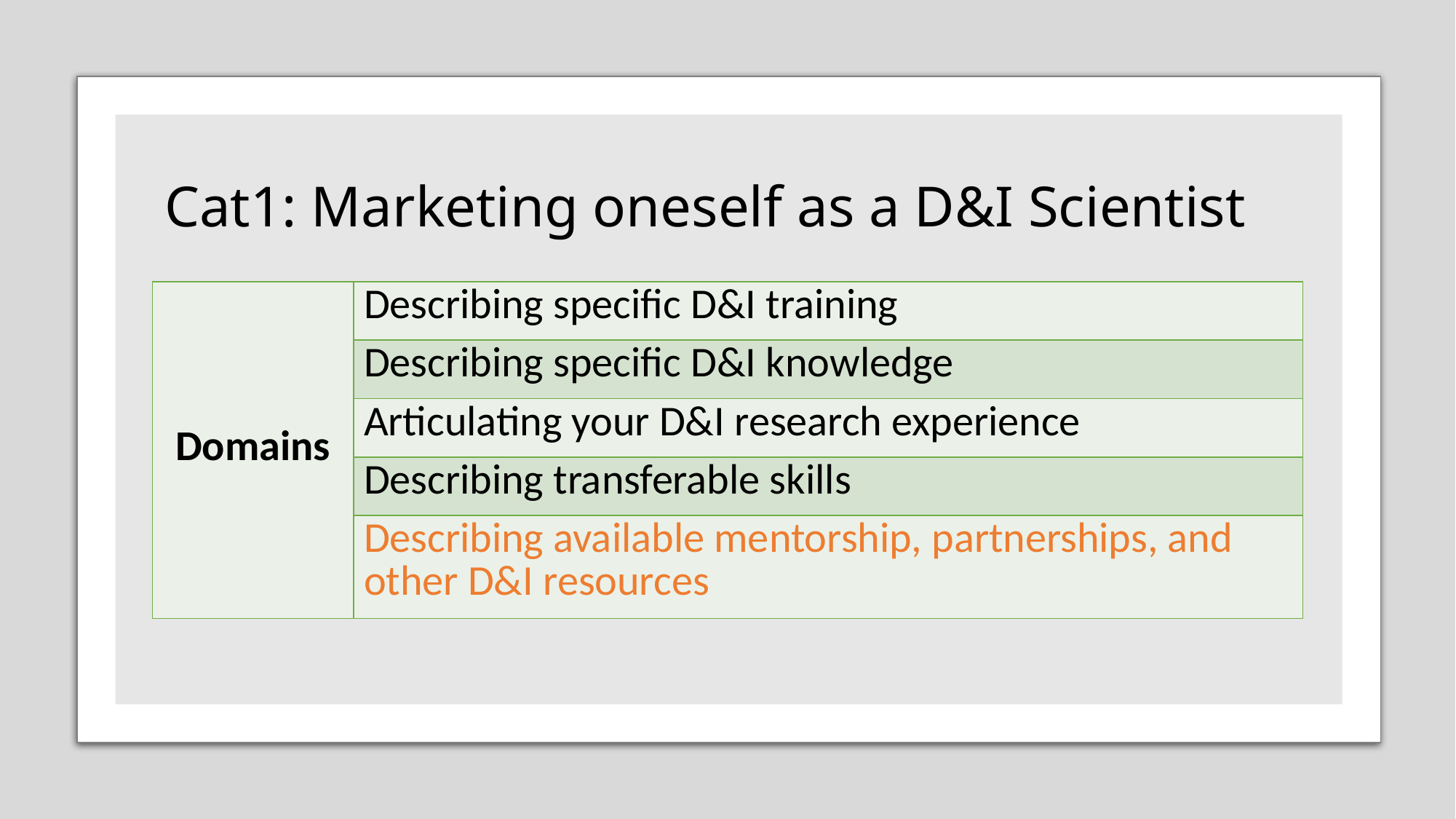

# Cat1: Marketing oneself as a D&I Scientist
| Domains | Describing specific D&I training |
| --- | --- |
| | Describing specific D&I knowledge |
| | Articulating your D&I research experience |
| | Describing transferable skills |
| | Describing available mentorship, partnerships, and other D&I resources |

## Slide 5
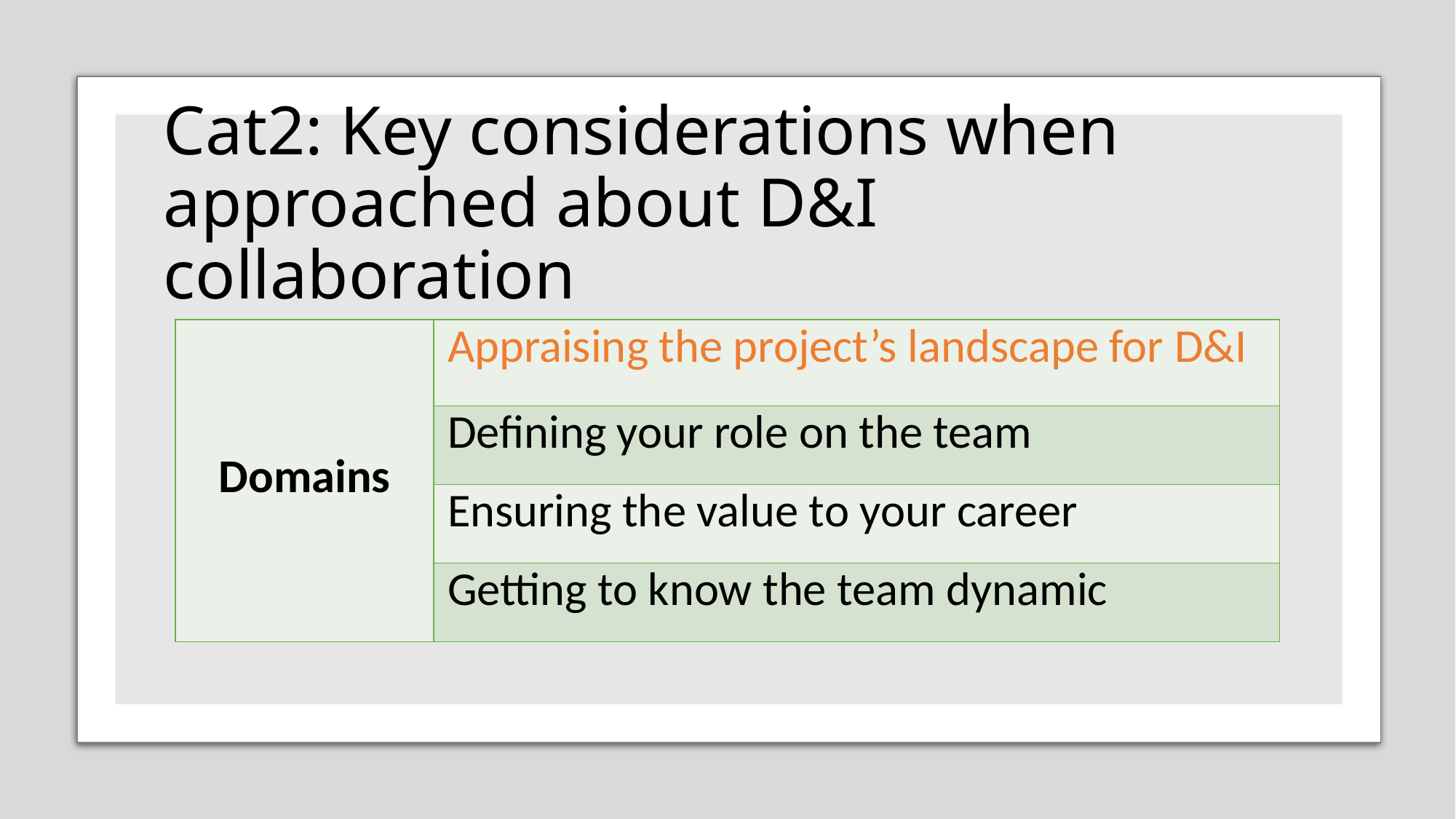

# Cat2: Key considerations when approached about D&I collaboration
| Domains | Appraising the project’s landscape for D&I |
| --- | --- |
| | Defining your role on the team |
| | Ensuring the value to your career |
| | Getting to know the team dynamic |

## Slide 6
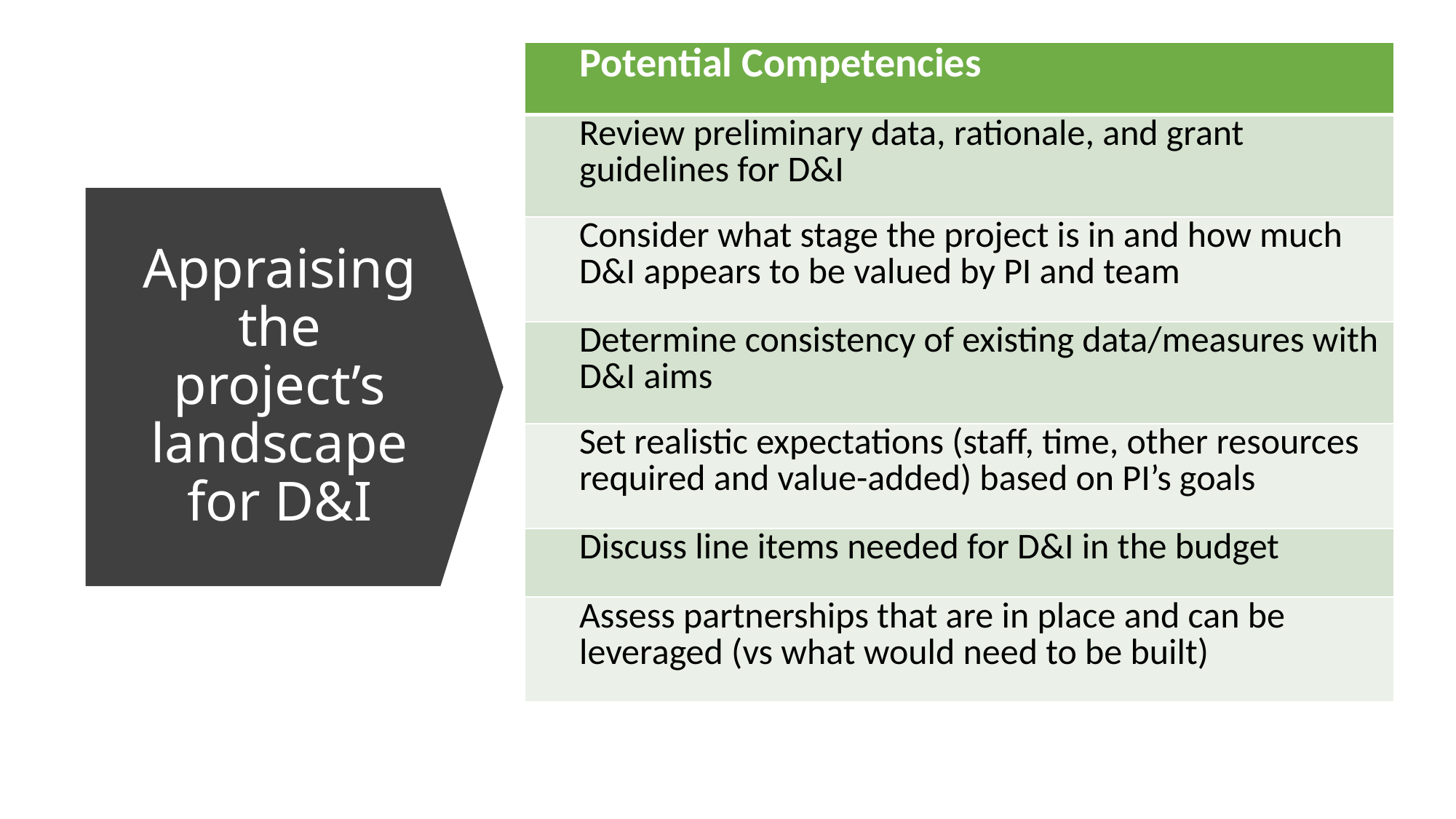

| Potential Competencies |
| --- |
| Review preliminary data, rationale, and grant guidelines for D&I |
| Consider what stage the project is in and how much D&I appears to be valued by PI and team |
| Determine consistency of existing data/measures with D&I aims |
| Set realistic expectations (staff, time, other resources required and value-added) based on PI’s goals |
| Discuss line items needed for D&I in the budget |
| Assess partnerships that are in place and can be leveraged (vs what would need to be built) |
# Appraising the project’s landscape for D&I

## Slide 7
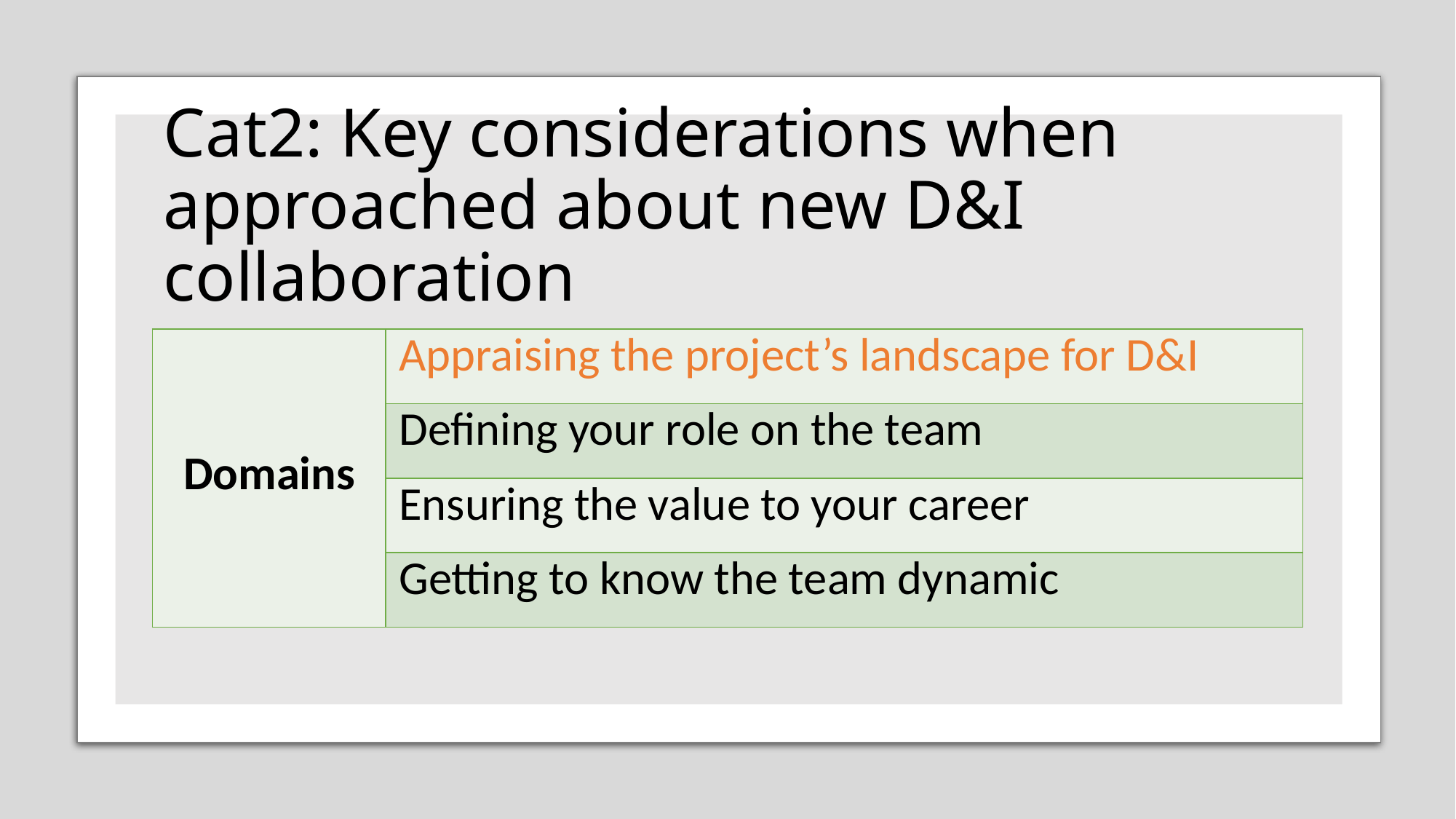

# Cat2: Key considerations when approached about new D&I collaboration
| Domains | Appraising the project’s landscape for D&I |
| --- | --- |
| | Defining your role on the team |
| | Ensuring the value to your career |
| | Getting to know the team dynamic |

## Slide 8
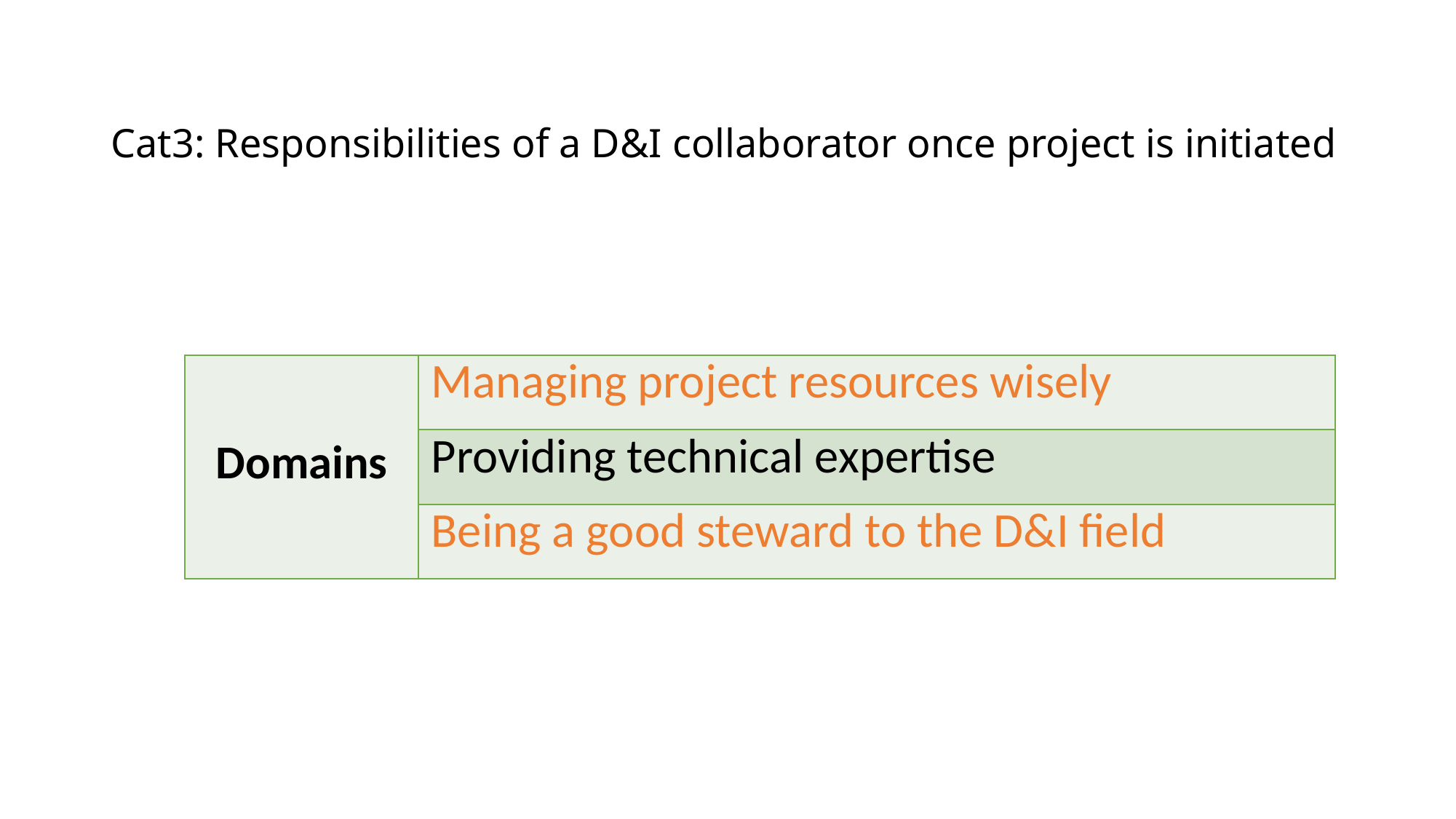

# Cat3: Responsibilities of a D&I collaborator once project is initiated
| Domains | Managing project resources wisely |
| --- | --- |
| | Providing technical expertise |
| | Being a good steward to the D&I field |

## Slide 9
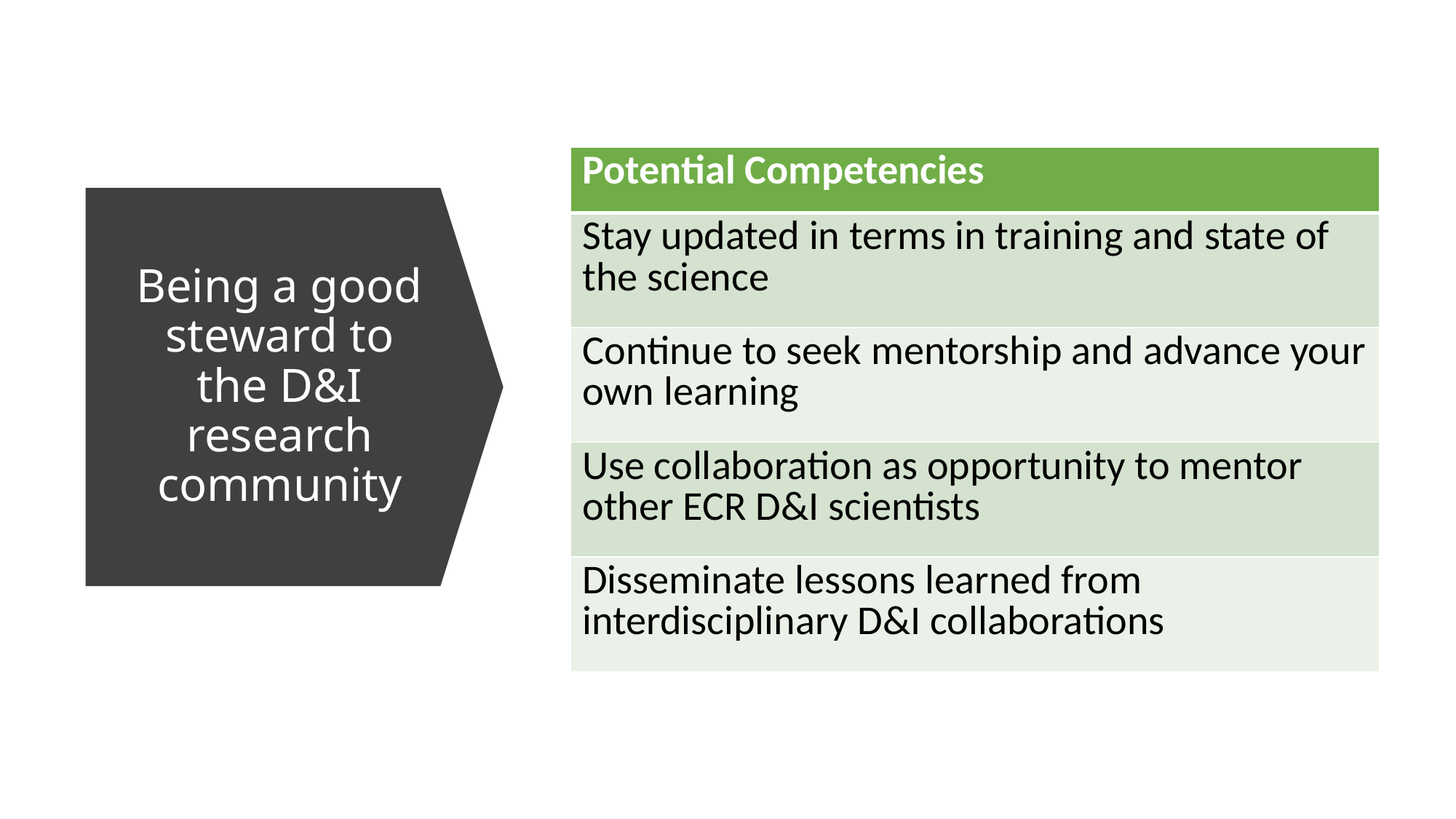

| Potential Competencies |
| --- |
| Stay updated in terms in training and state of the science |
| Continue to seek mentorship and advance your own learning |
| Use collaboration as opportunity to mentor other ECR D&I scientists |
| Disseminate lessons learned from interdisciplinary D&I collaborations |
# Being a good steward to the D&I research community

## Slide 10
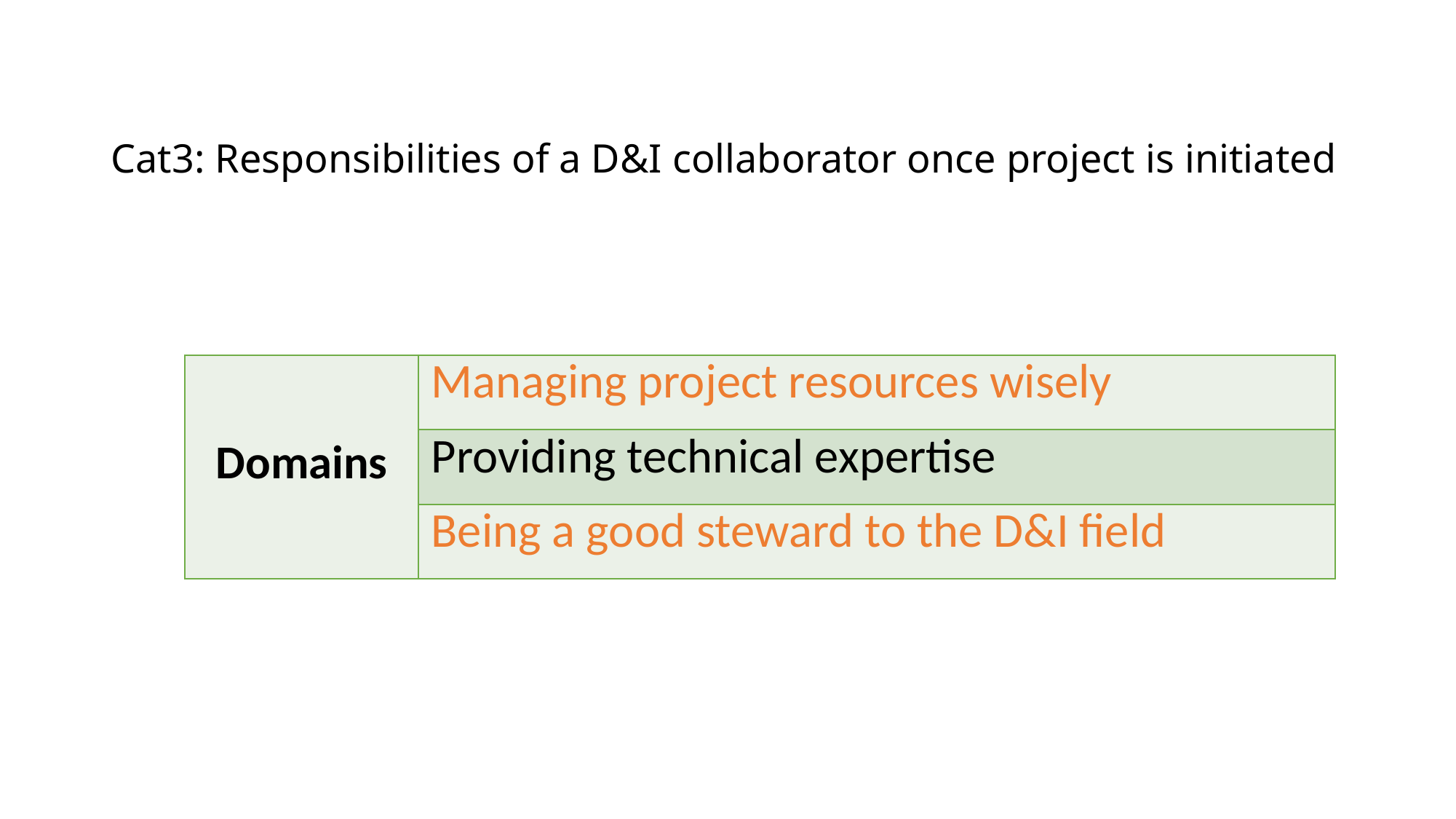

# Cat3: Responsibilities of a D&I collaborator once project is initiated
| Domains | Managing project resources wisely |
| --- | --- |
| | Providing technical expertise |
| | Being a good steward to the D&I field |

## Slide 11
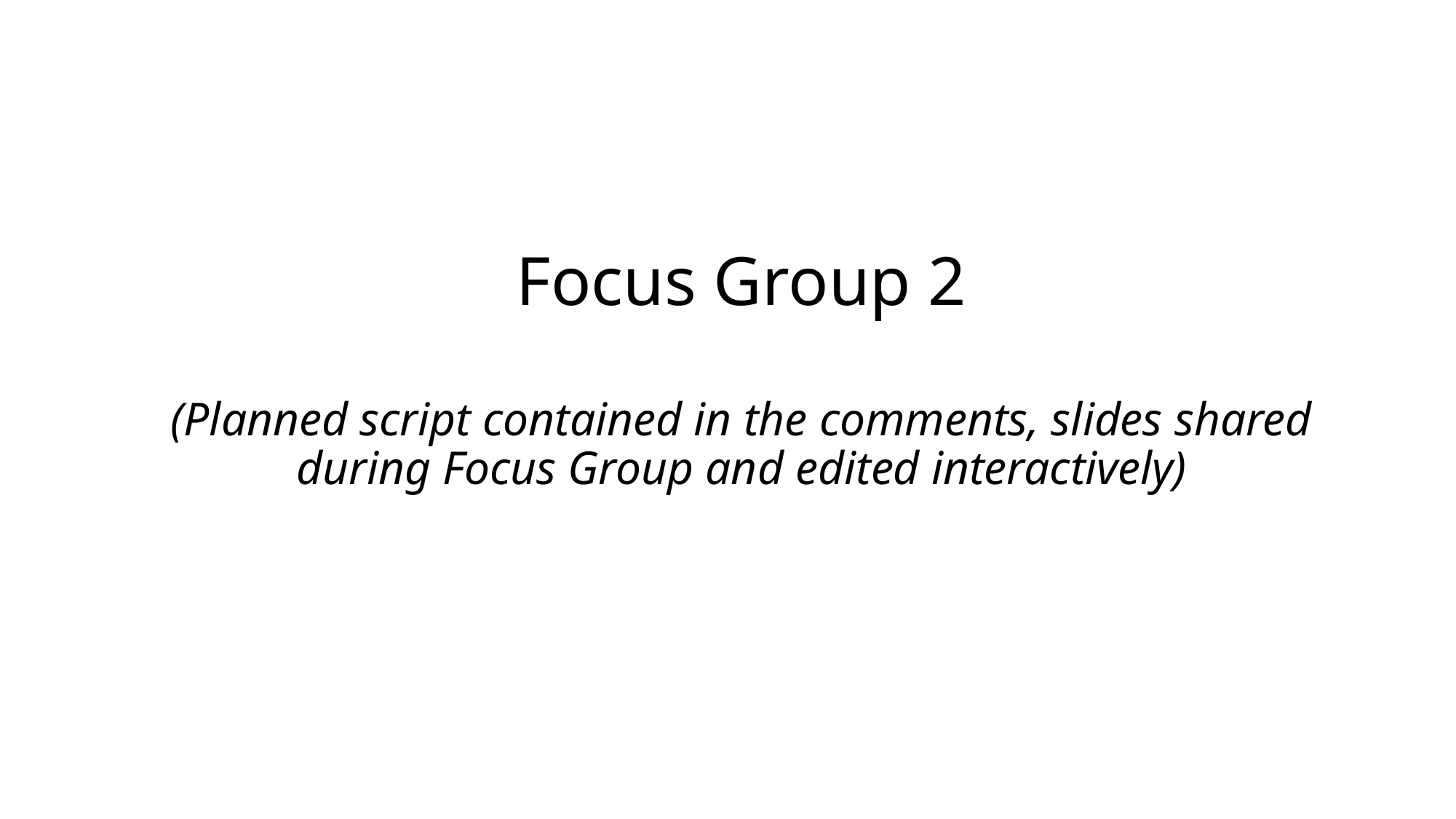

# Focus Group 2
(Planned script contained in the comments, slides shared during Focus Group and edited interactively)

## Slide 12
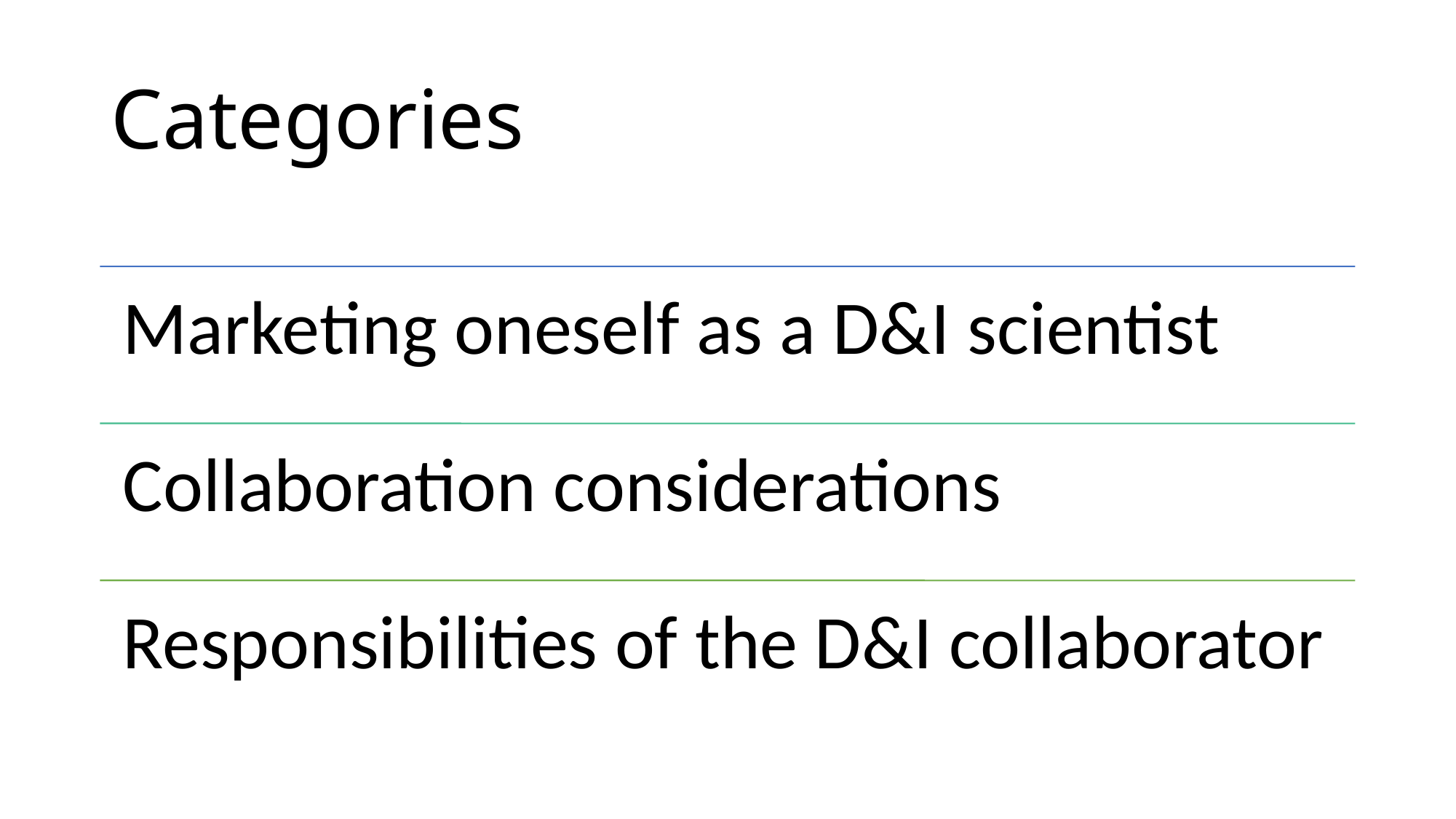

# Categories

## Slide 13
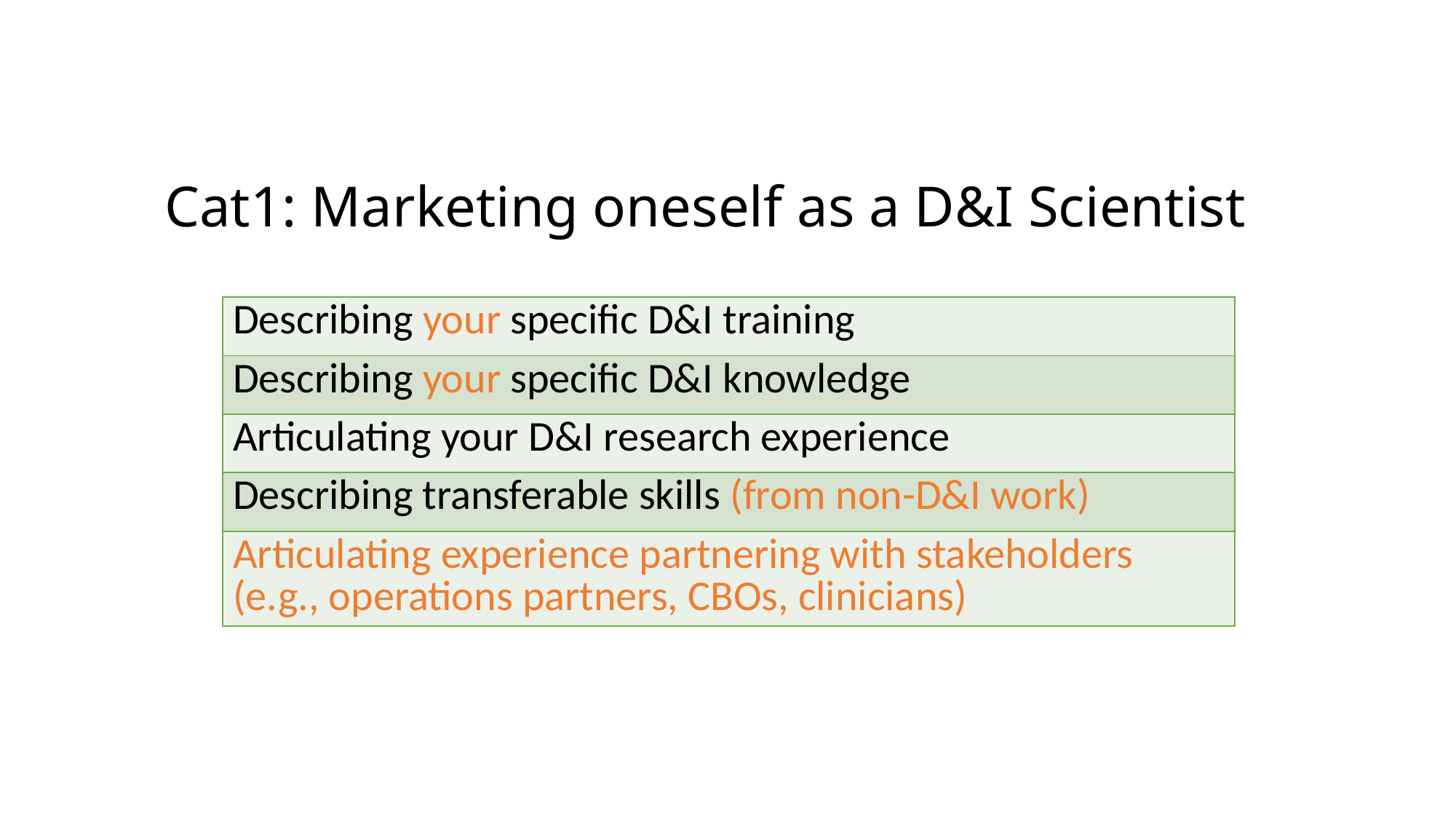

# Cat1: Marketing oneself as a D&I Scientist
| Describing your specific D&I training |
| --- |
| Describing your specific D&I knowledge |
| Articulating your D&I research experience |
| Describing transferable skills (from non-D&I work) |
| Articulating experience partnering with stakeholders (e.g., operations partners, CBOs, clinicians) |

## Slide 14
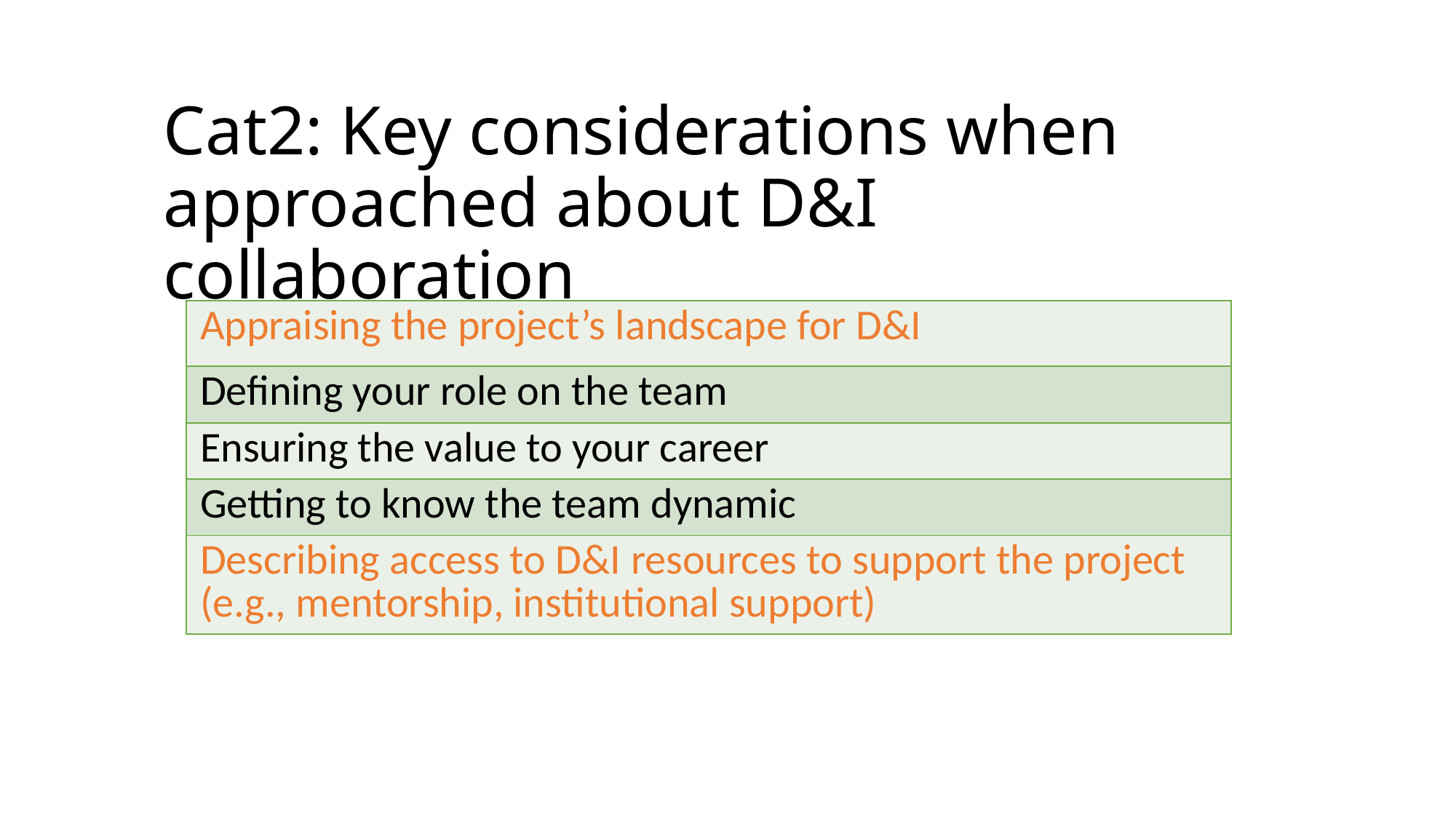

# Cat2: Key considerations when approached about D&I collaboration
| Appraising the project’s landscape for D&I |
| --- |
| Defining your role on the team |
| Ensuring the value to your career |
| Getting to know the team dynamic |
| Describing access to D&I resources to support the project (e.g., mentorship, institutional support) |

## Slide 15
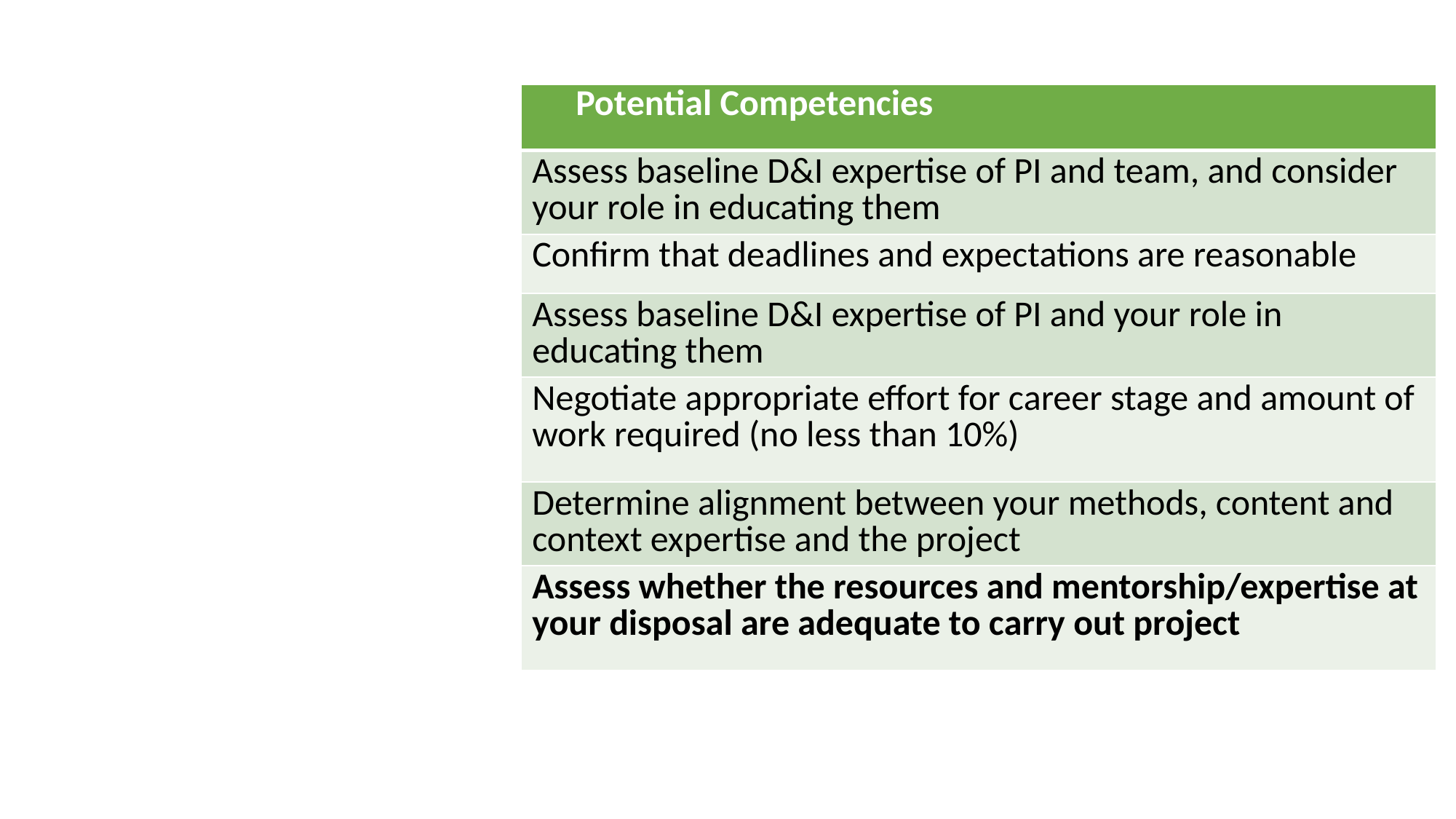

| Potential Competencies |
| --- |
| Assess baseline D&I expertise of PI and team, and consider your role in educating them |
| Confirm that deadlines and expectations are reasonable |
| Assess baseline D&I expertise of PI and your role in educating them |
| Negotiate appropriate effort for career stage and amount of work required (no less than 10%) |
| Determine alignment between your methods, content and context expertise and the project |
| Assess whether the resources and mentorship/expertise at your disposal are adequate to carry out project |
# Defining your role on the team

## Slide 16
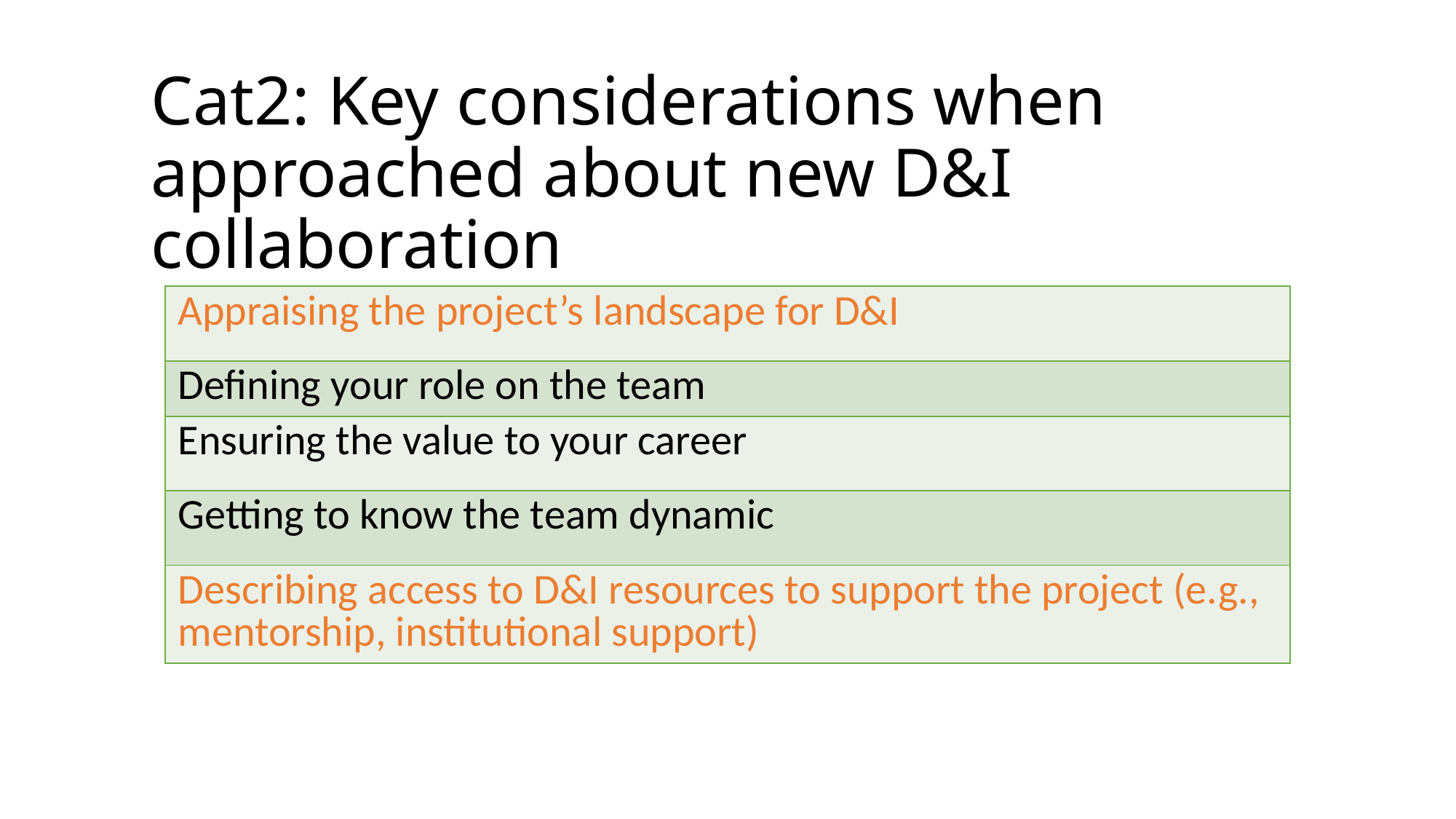

# Cat2: Key considerations when approached about new D&I collaboration
| Appraising the project’s landscape for D&I |
| --- |
| Defining your role on the team |
| Ensuring the value to your career |
| Getting to know the team dynamic |
| Describing access to D&I resources to support the project (e.g., mentorship, institutional support) |

## Slide 17
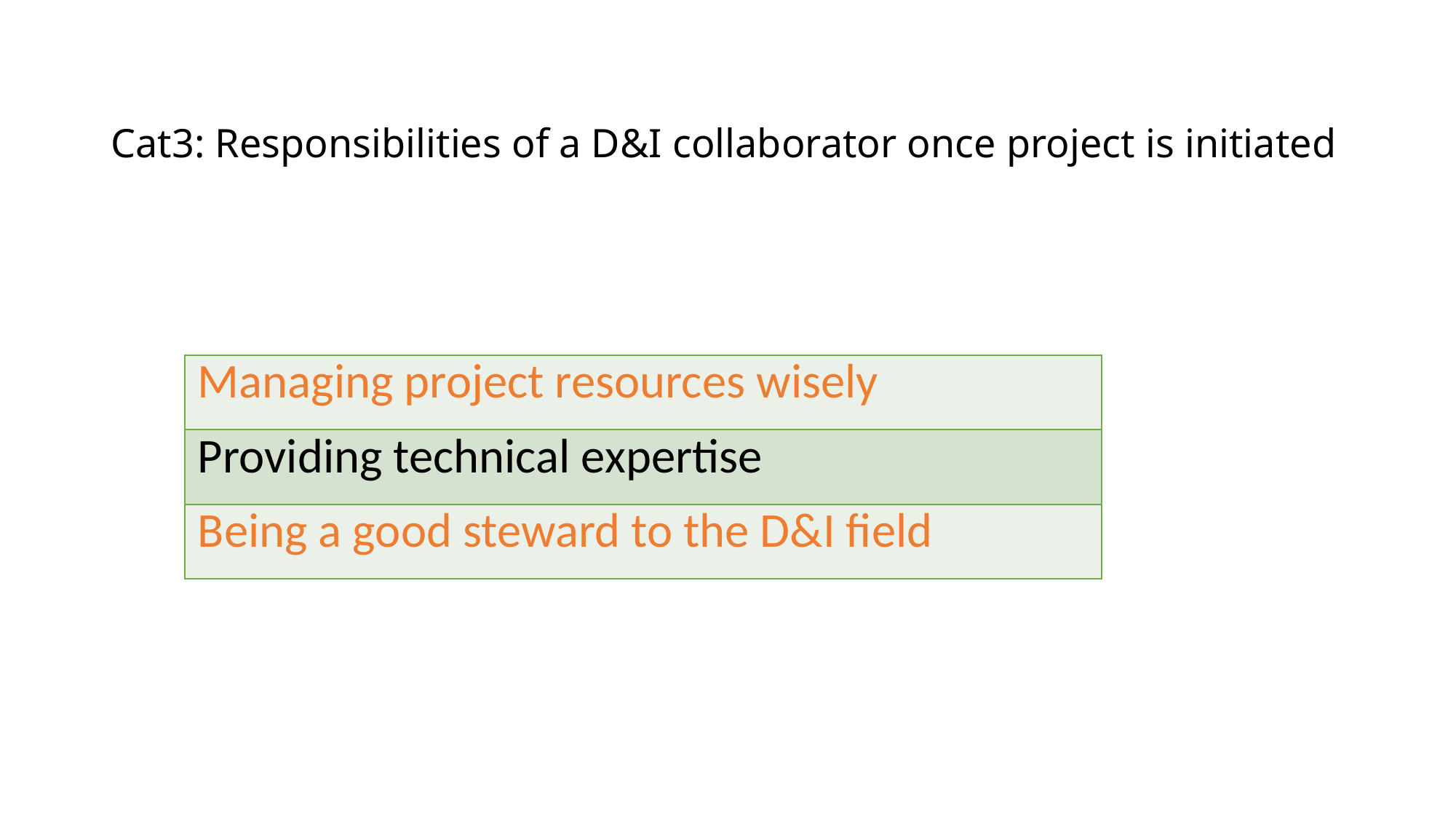

# Cat3: Responsibilities of a D&I collaborator once project is initiated
| Managing project resources wisely |
| --- |
| Providing technical expertise |
| Being a good steward to the D&I field |

## Slide 18
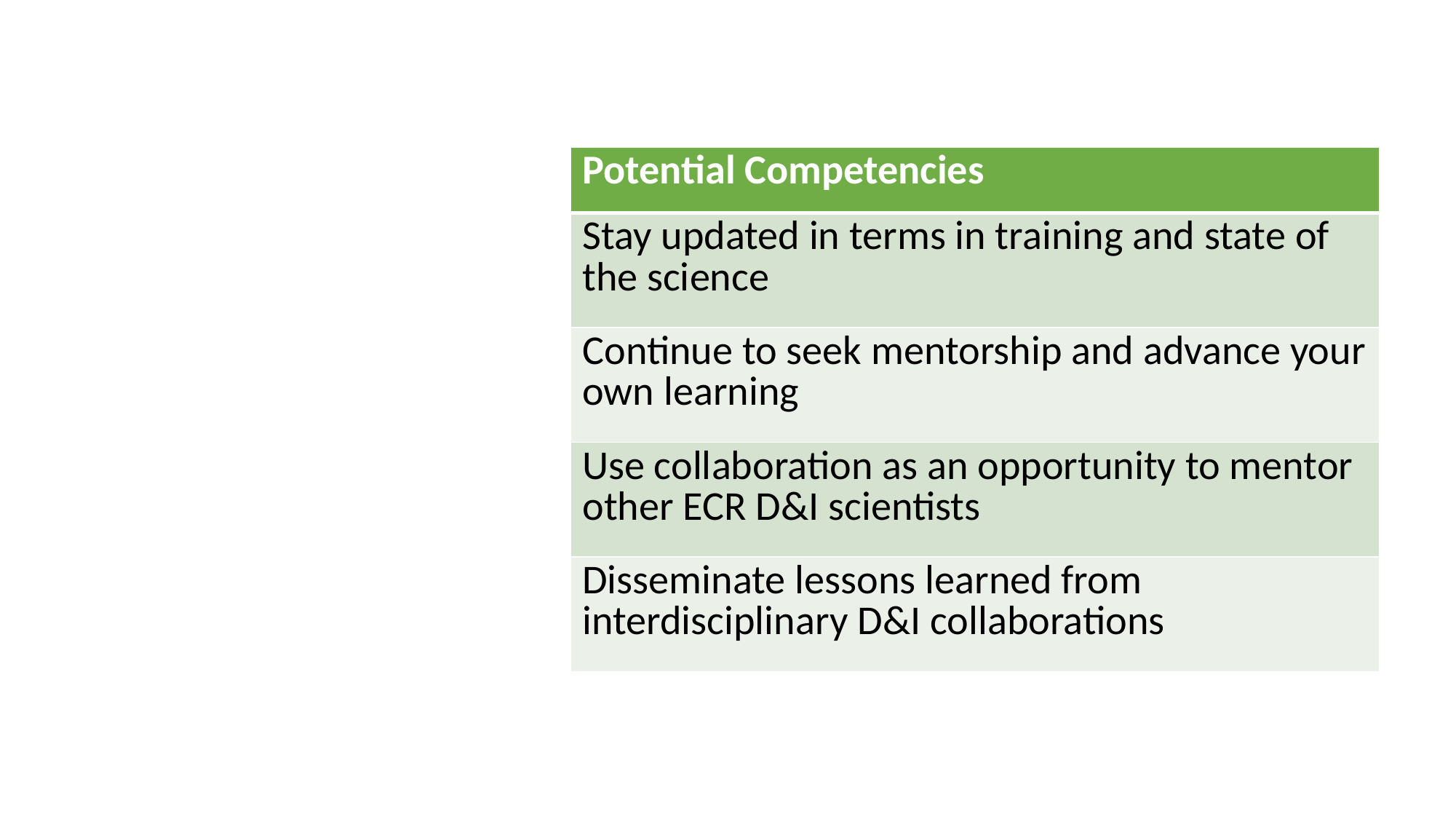

| Potential Competencies |
| --- |
| Stay updated in terms in training and state of the science |
| Continue to seek mentorship and advance your own learning |
| Use collaboration as an opportunity to mentor other ECR D&I scientists |
| Disseminate lessons learned from interdisciplinary D&I collaborations |
# Being a good steward to the D&I research community

## Slide 19
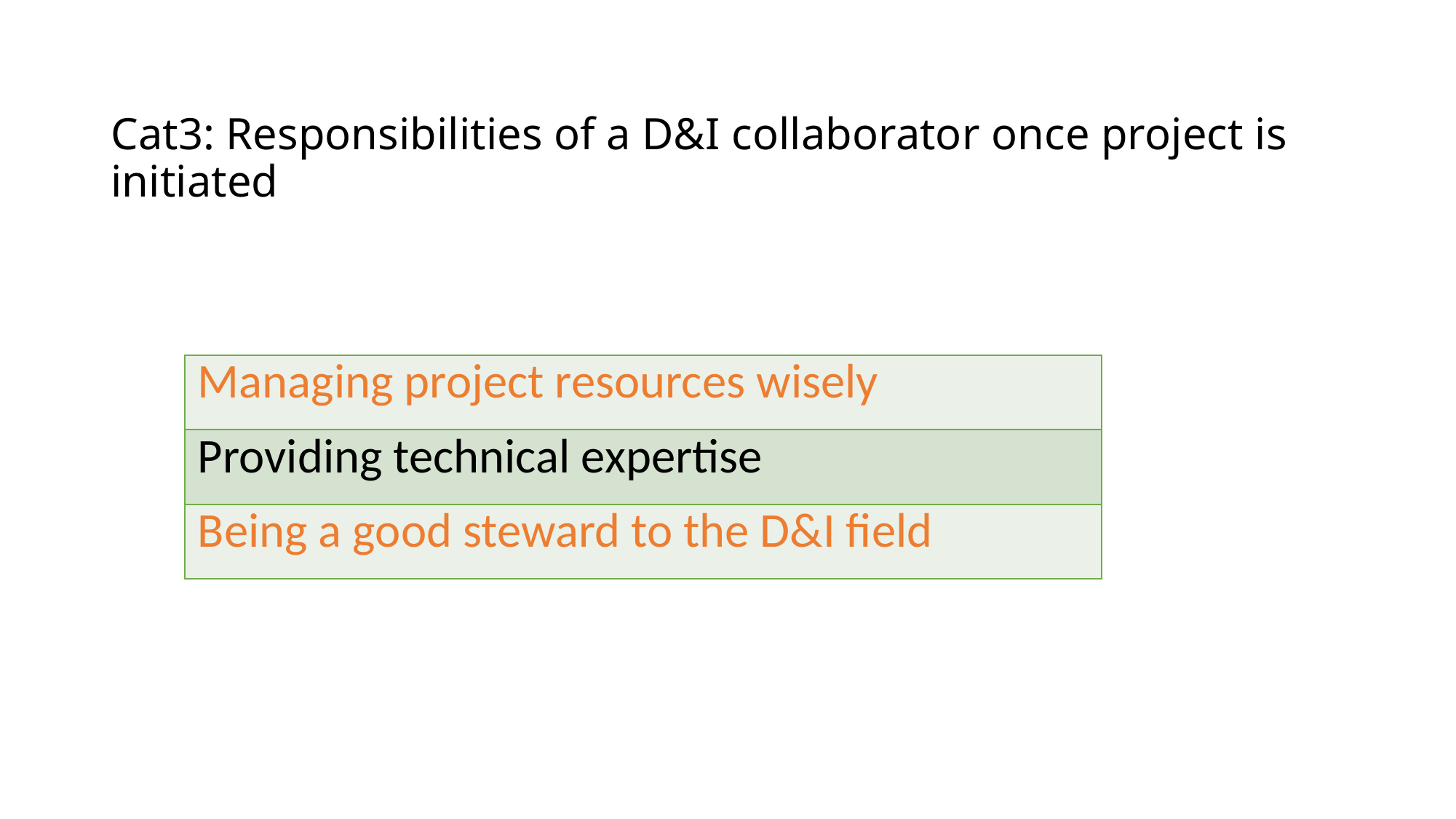

# Cat3: Responsibilities of a D&I collaborator once project is initiated
| Managing project resources wisely |
| --- |
| Providing technical expertise |
| Being a good steward to the D&I field |

## Slide 20
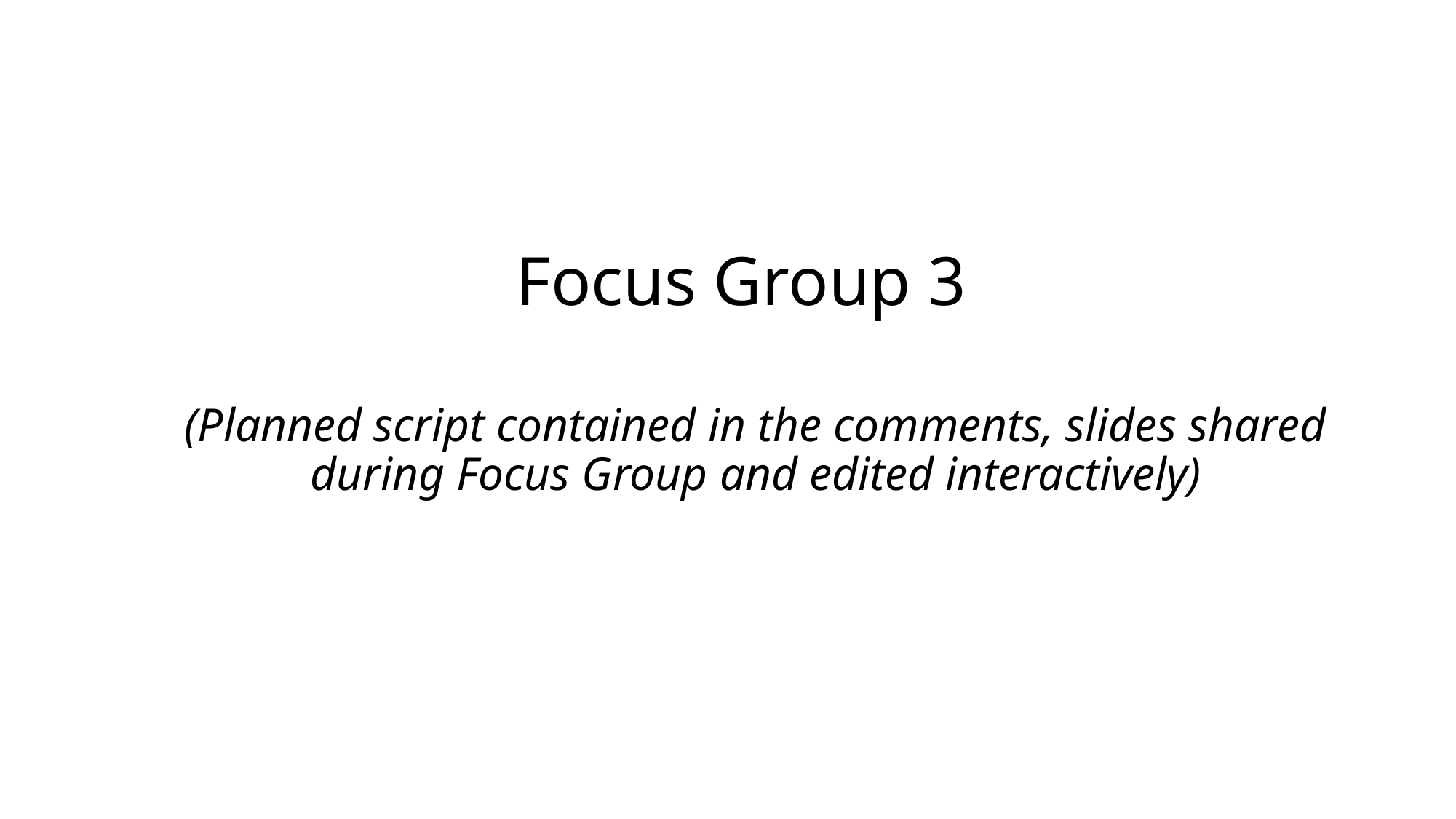

# Focus Group 3
(Planned script contained in the comments, slides shared during Focus Group and edited interactively)

## Slide 21
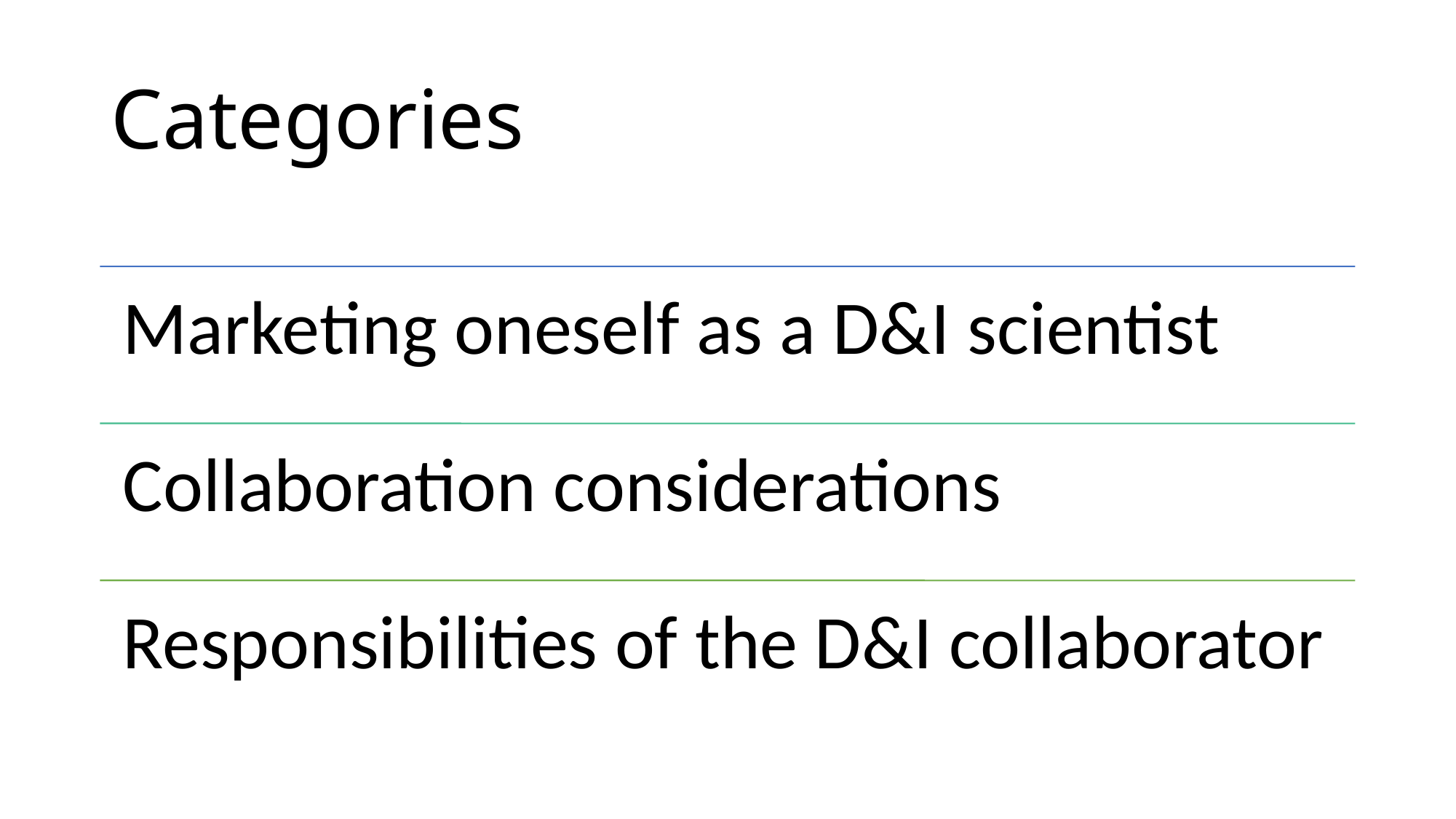

# Categories

## Slide 22
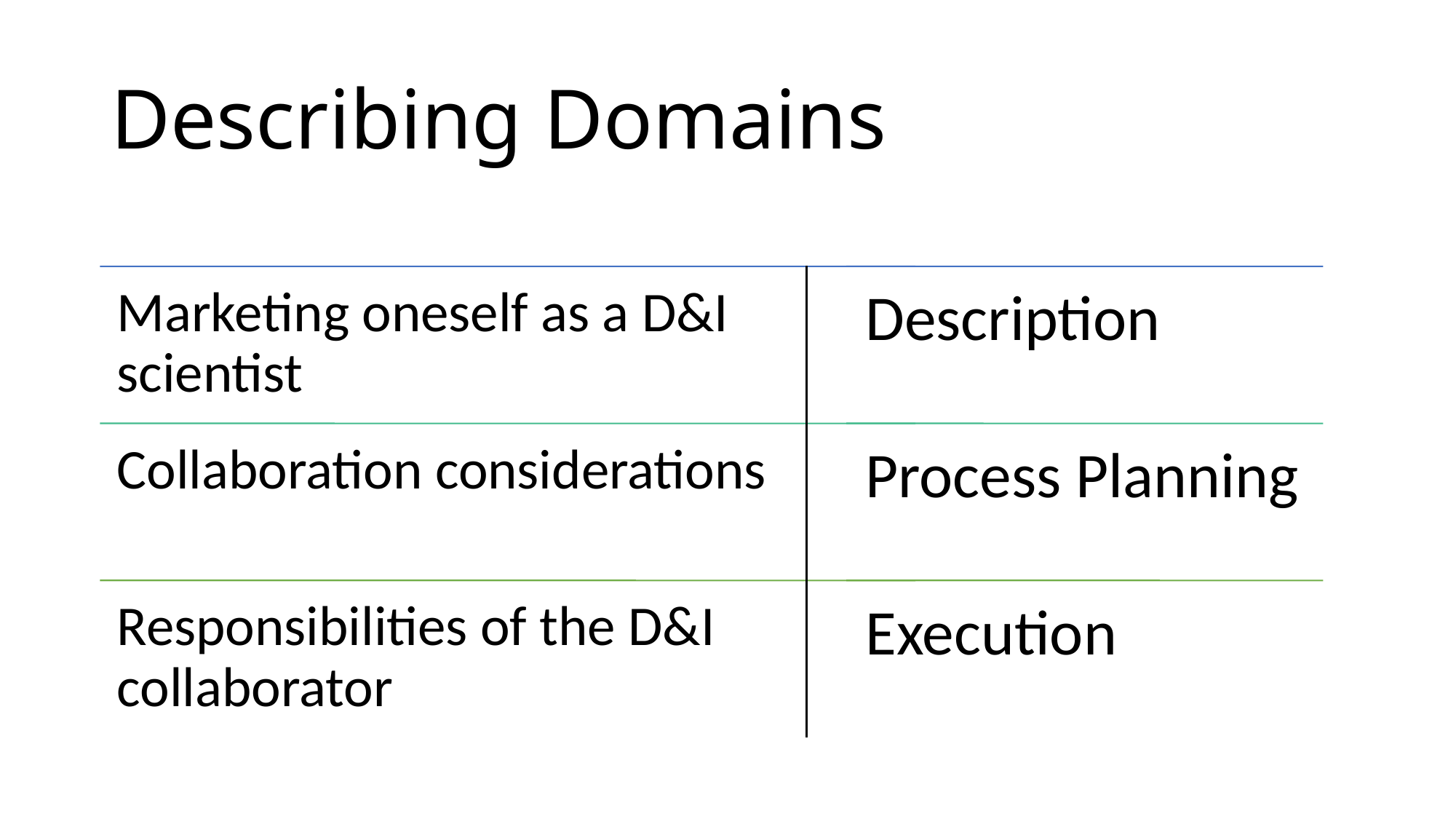

# Describing Domains

## Slide 23
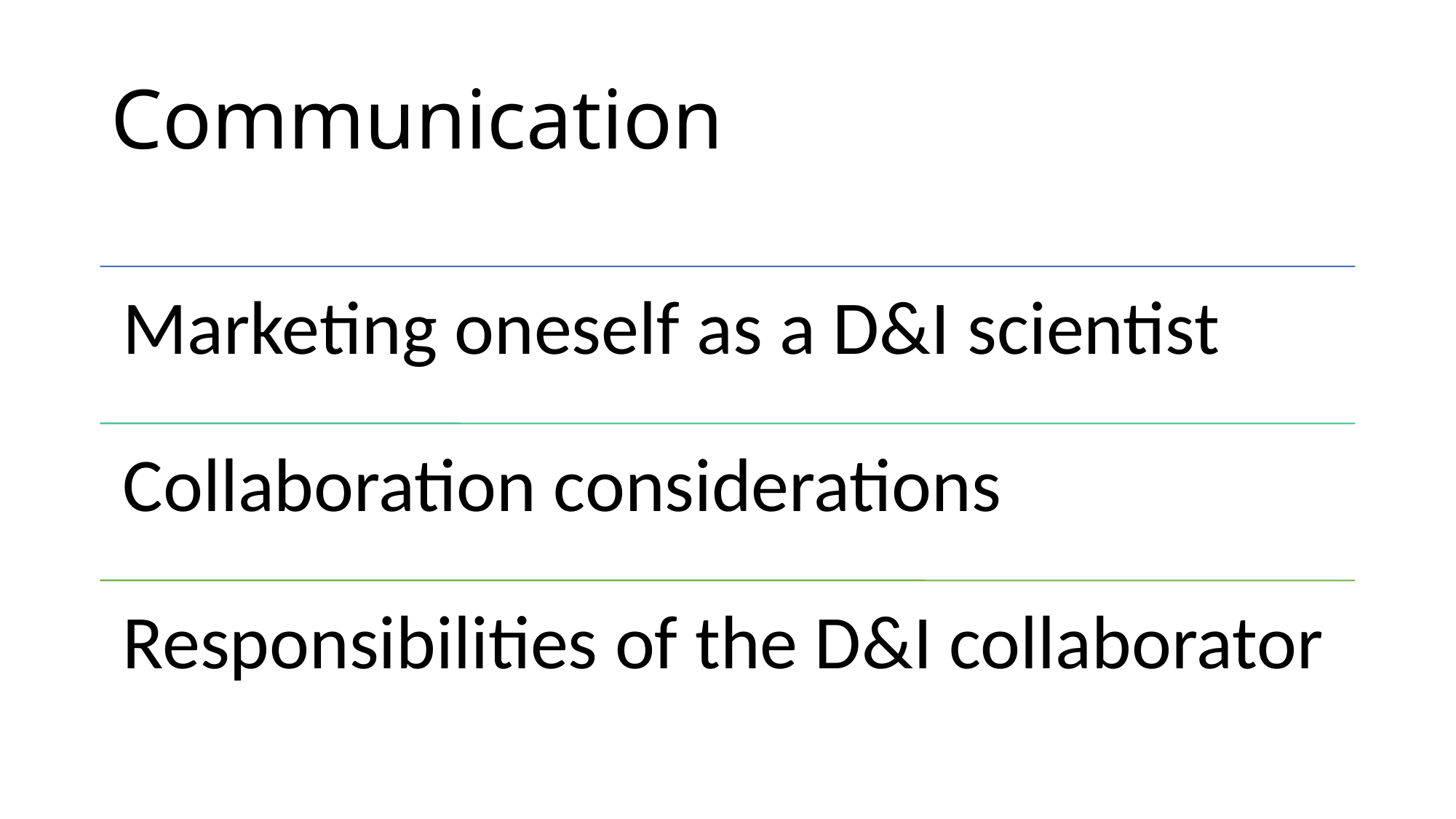

# Communication

## Slide 24
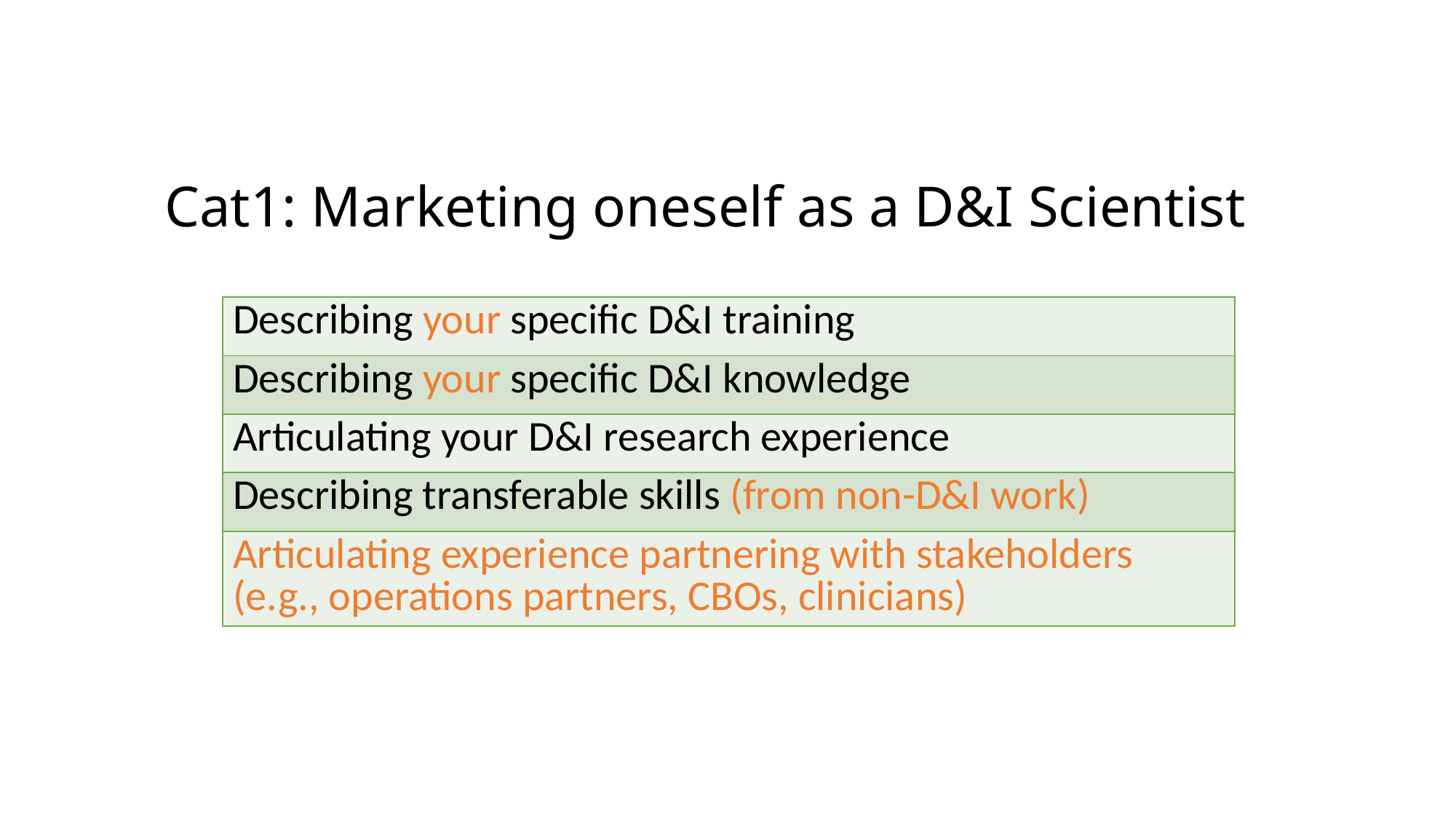

# Cat1: Marketing oneself as a D&I Scientist
| Describing your specific D&I training |
| --- |
| Describing your specific D&I knowledge |
| Articulating your D&I research experience |
| Describing transferable skills (from non-D&I work) |
| Articulating experience partnering with stakeholders (e.g., operations partners, CBOs, clinicians) |

## Slide 25
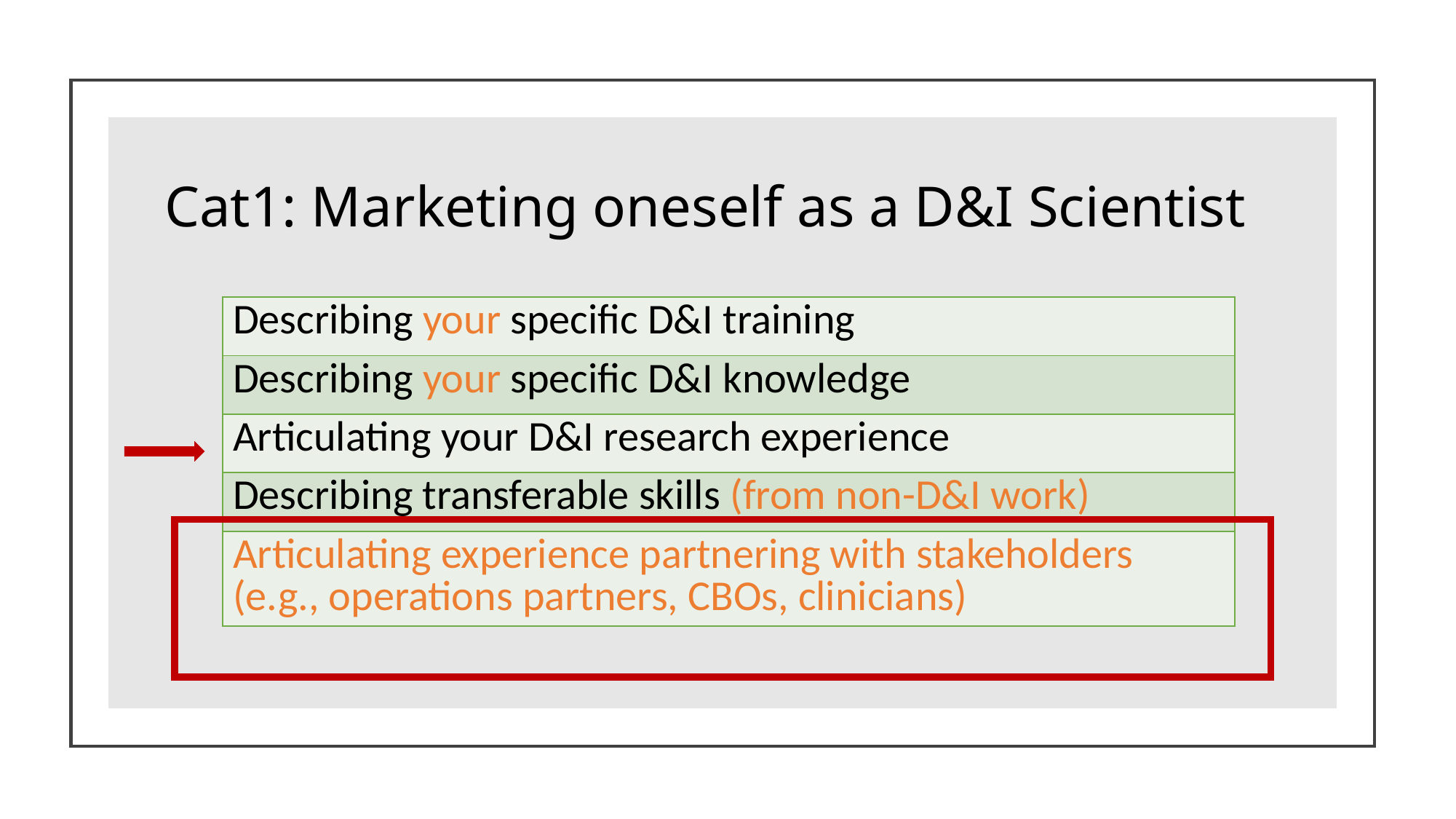

# Cat1: Marketing oneself as a D&I Scientist
| Describing your specific D&I training |
| --- |
| Describing your specific D&I knowledge |
| Articulating your D&I research experience |
| Describing transferable skills (from non-D&I work) |
| Articulating experience partnering with stakeholders (e.g., operations partners, CBOs, clinicians) |

## Slide 26
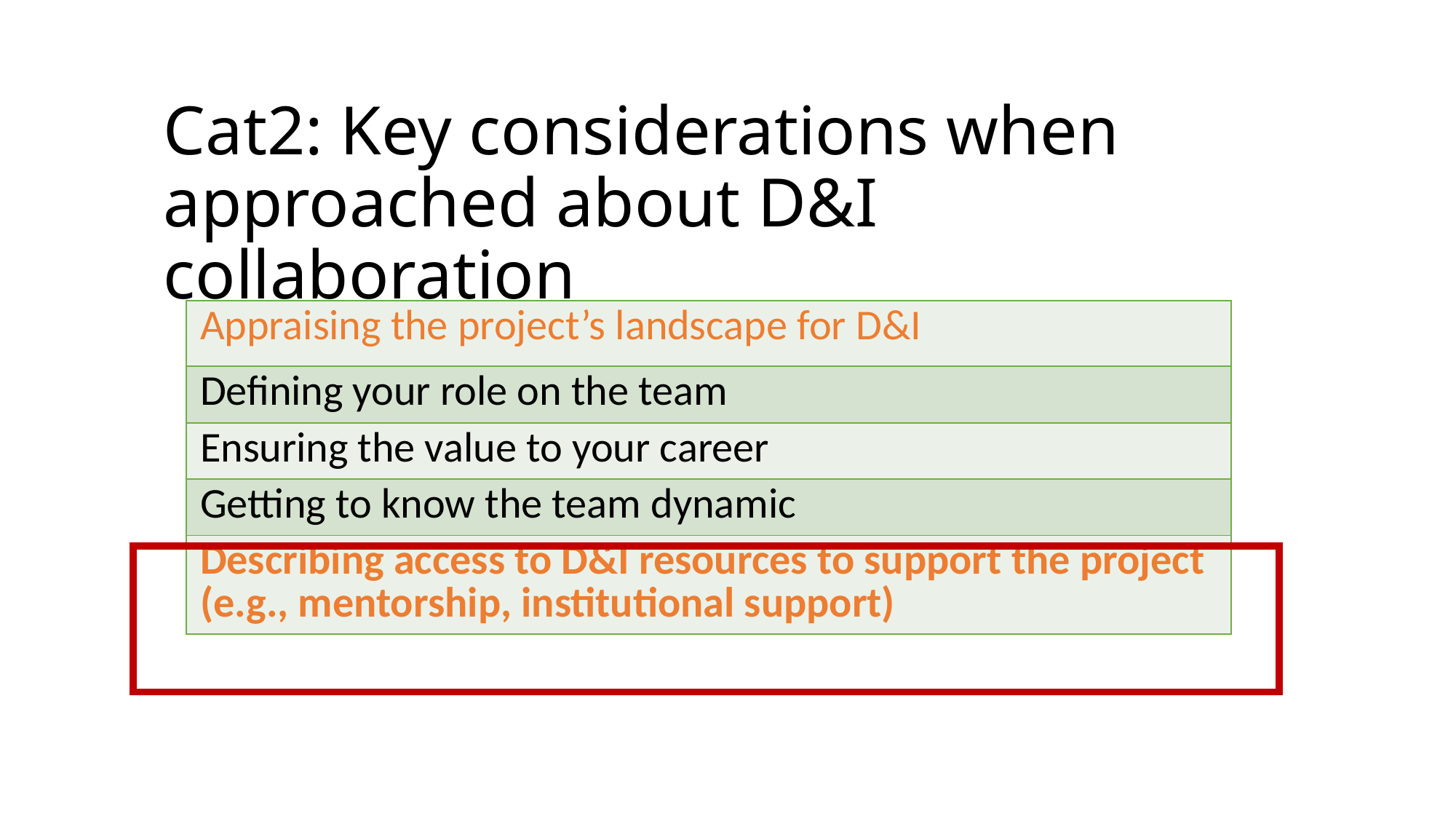

# Cat2: Key considerations when approached about D&I collaboration
| Appraising the project’s landscape for D&I |
| --- |
| Defining your role on the team |
| Ensuring the value to your career |
| Getting to know the team dynamic |
| Describing access to D&I resources to support the project (e.g., mentorship, institutional support) |

## Slide 27
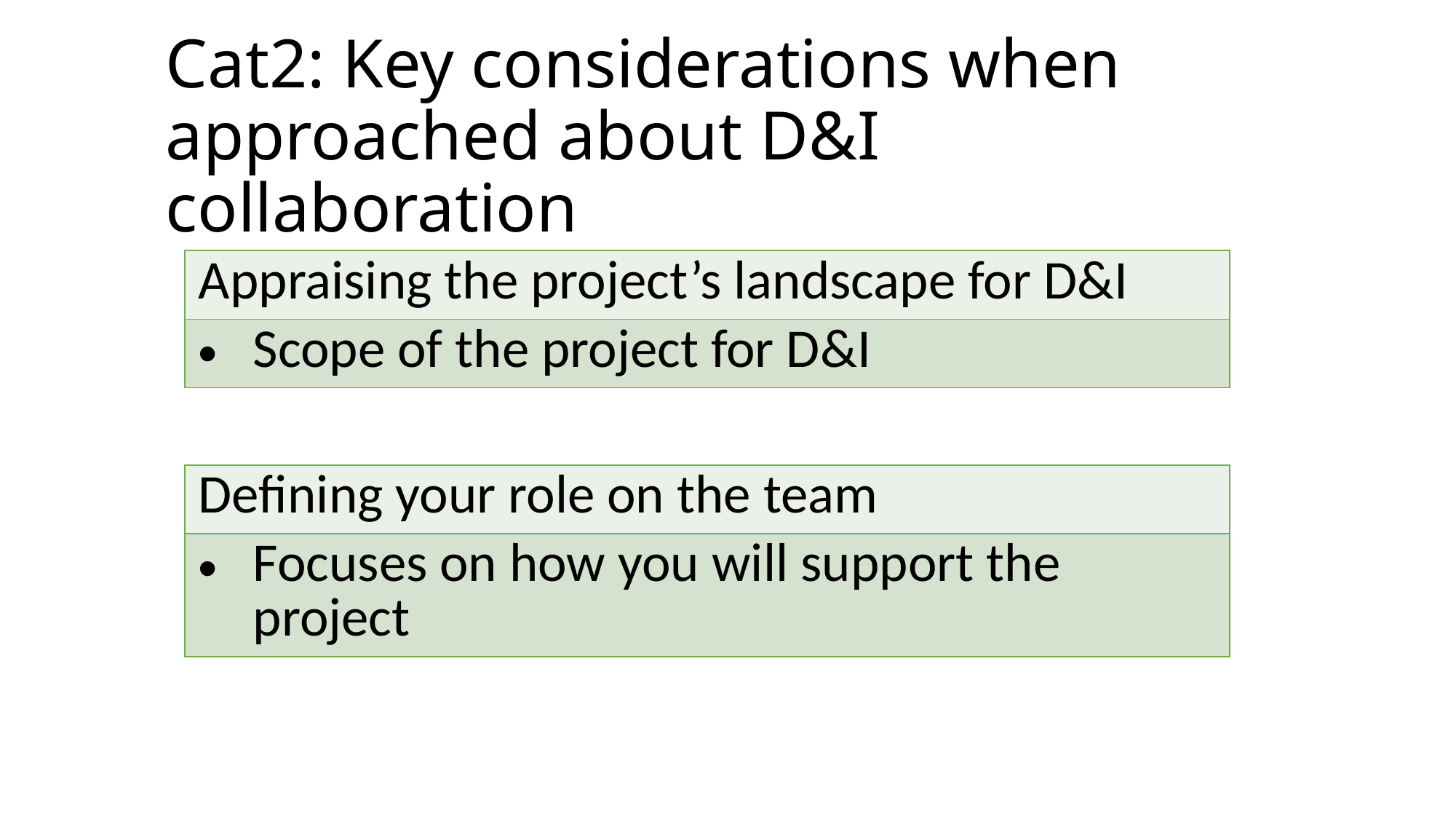

# Cat2: Key considerations when approached about D&I collaboration
| Appraising the project’s landscape for D&I |
| --- |
| Scope of the project for D&I |
| Defining your role on the team |
| --- |
| Focuses on how you will support the project |

## Slide 28
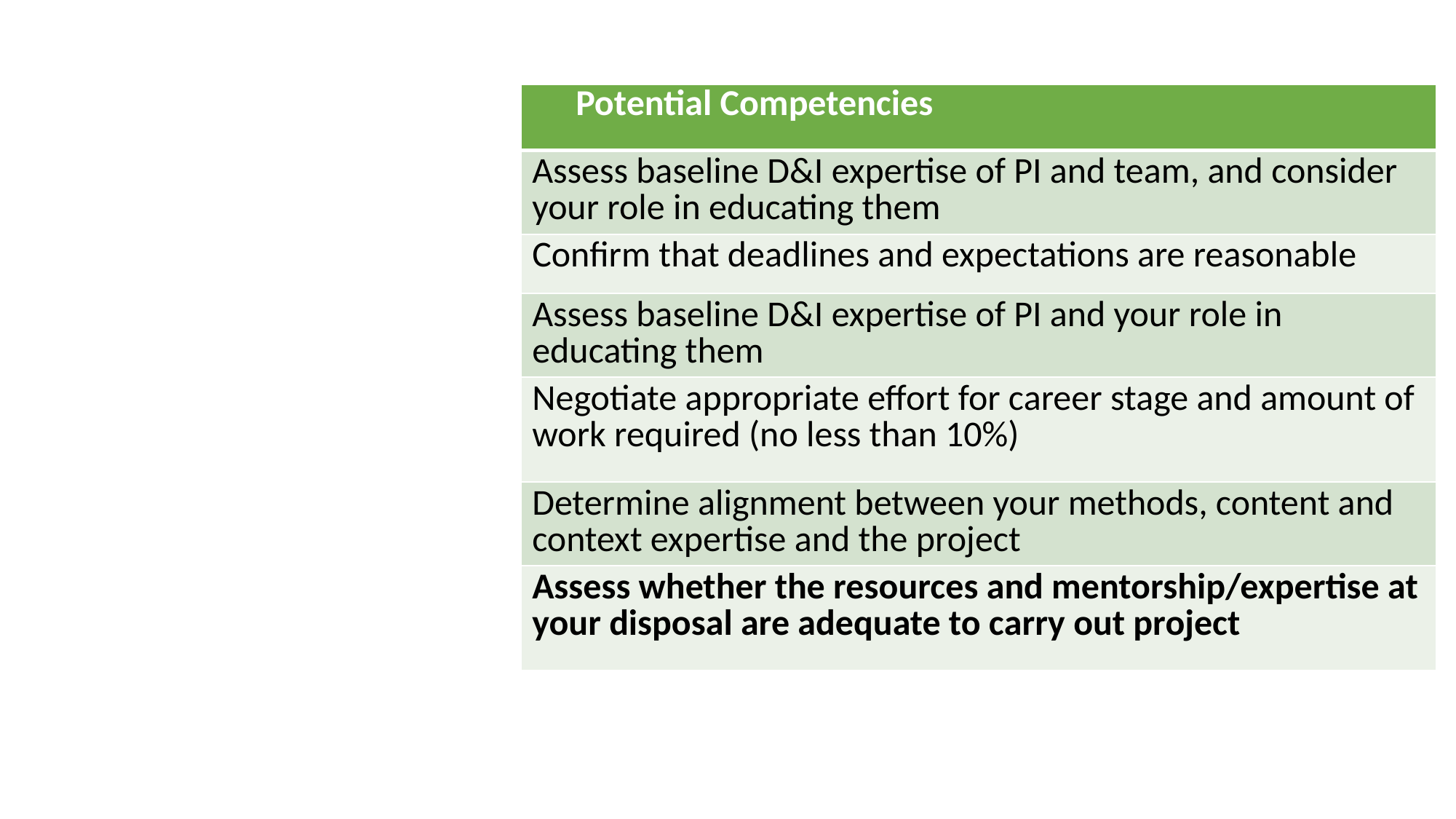

| Potential Competencies |
| --- |
| Assess baseline D&I expertise of PI and team, and consider your role in educating them |
| Confirm that deadlines and expectations are reasonable |
| Assess baseline D&I expertise of PI and your role in educating them |
| Negotiate appropriate effort for career stage and amount of work required (no less than 10%) |
| Determine alignment between your methods, content and context expertise and the project |
| Assess whether the resources and mentorship/expertise at your disposal are adequate to carry out project |
# Defining your role on the team

## Slide 29
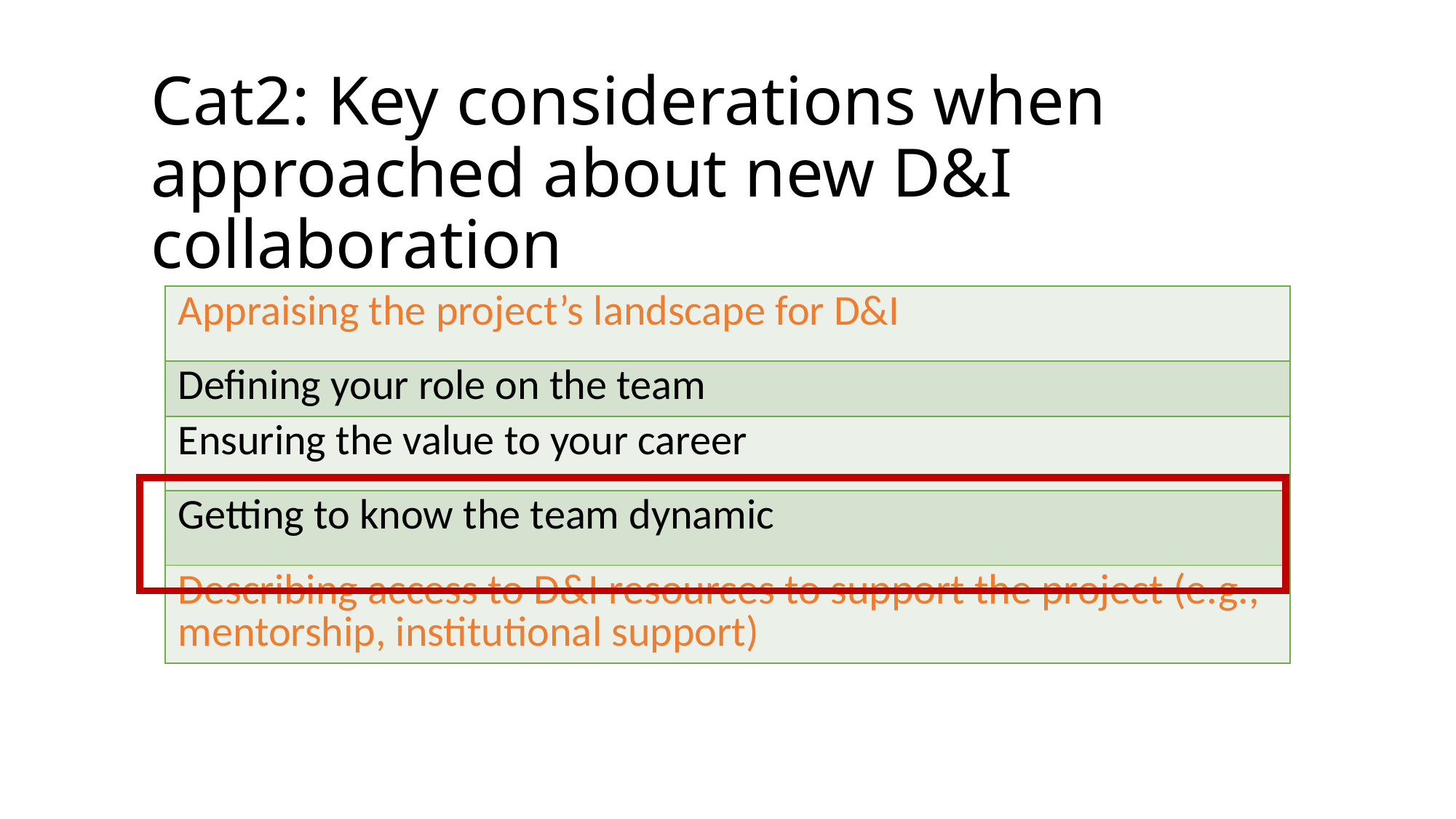

# Cat2: Key considerations when approached about new D&I collaboration
| Appraising the project’s landscape for D&I |
| --- |
| Defining your role on the team |
| Ensuring the value to your career |
| Getting to know the team dynamic |
| Describing access to D&I resources to support the project (e.g., mentorship, institutional support) |

## Slide 30
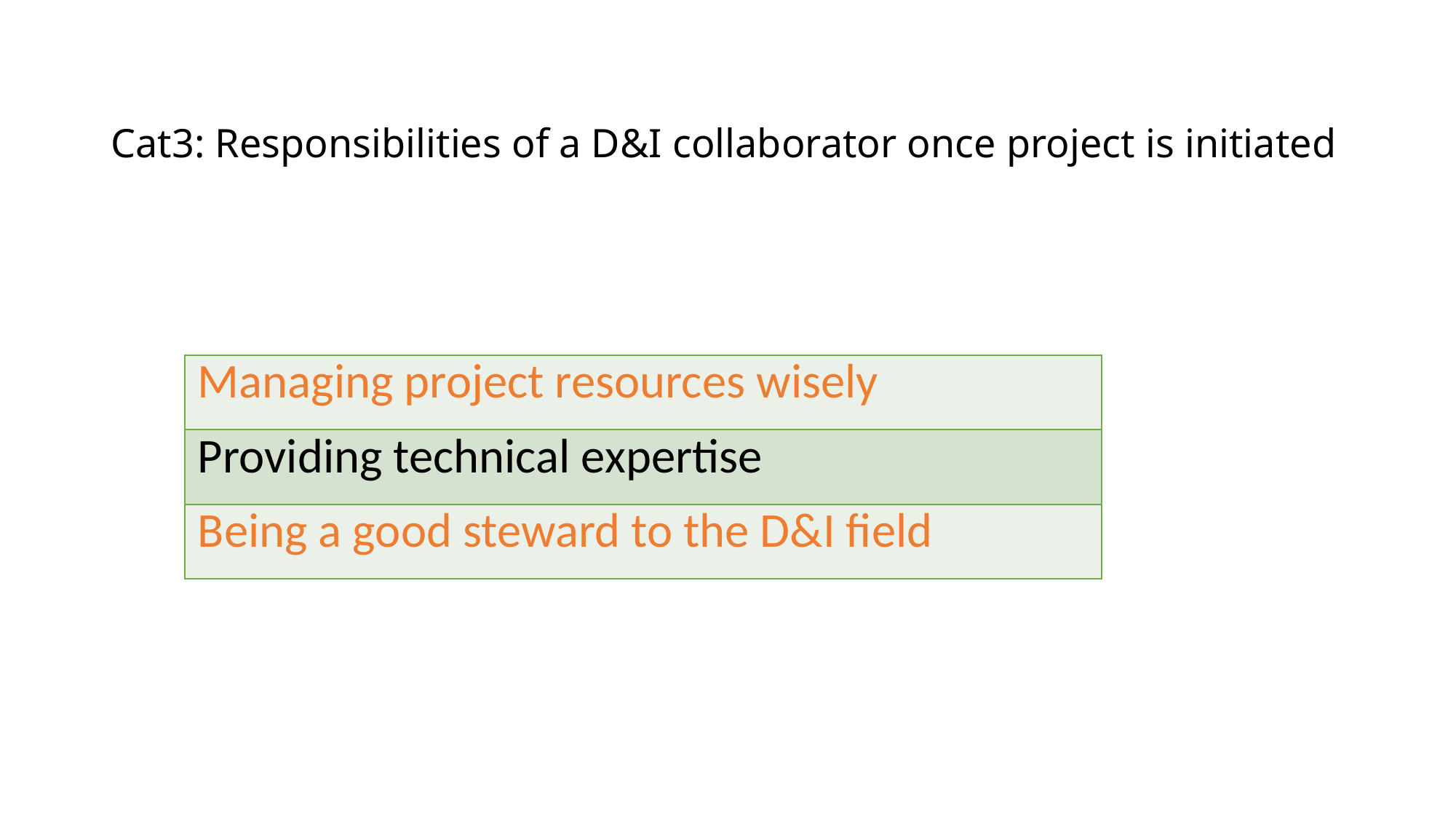

# Cat3: Responsibilities of a D&I collaborator once project is initiated
| Managing project resources wisely |
| --- |
| Providing technical expertise |
| Being a good steward to the D&I field |

## Slide 31
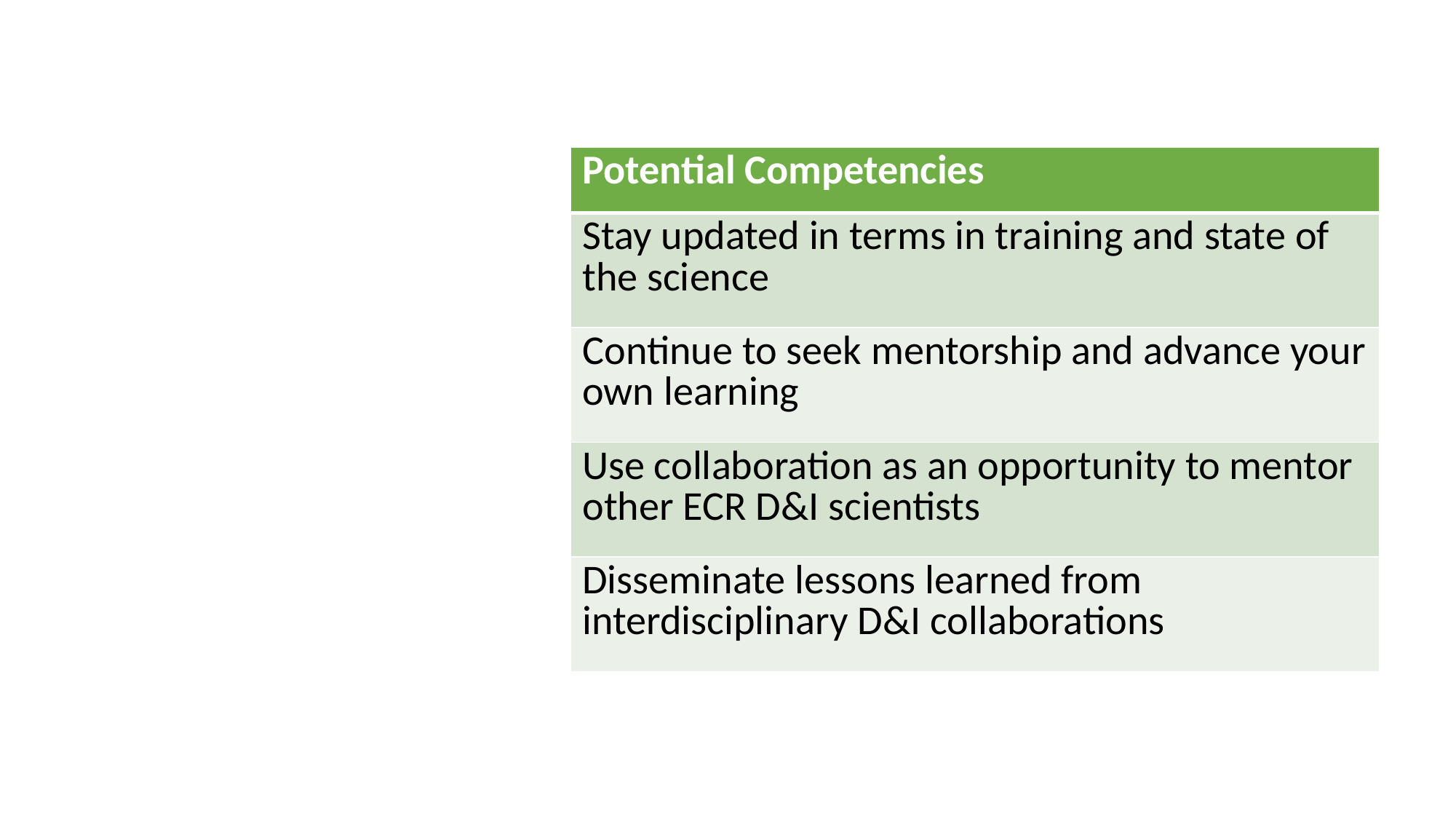

| Potential Competencies |
| --- |
| Stay updated in terms in training and state of the science |
| Continue to seek mentorship and advance your own learning |
| Use collaboration as an opportunity to mentor other ECR D&I scientists |
| Disseminate lessons learned from interdisciplinary D&I collaborations |
# Being a good steward to the D&I research community

## Slide 32
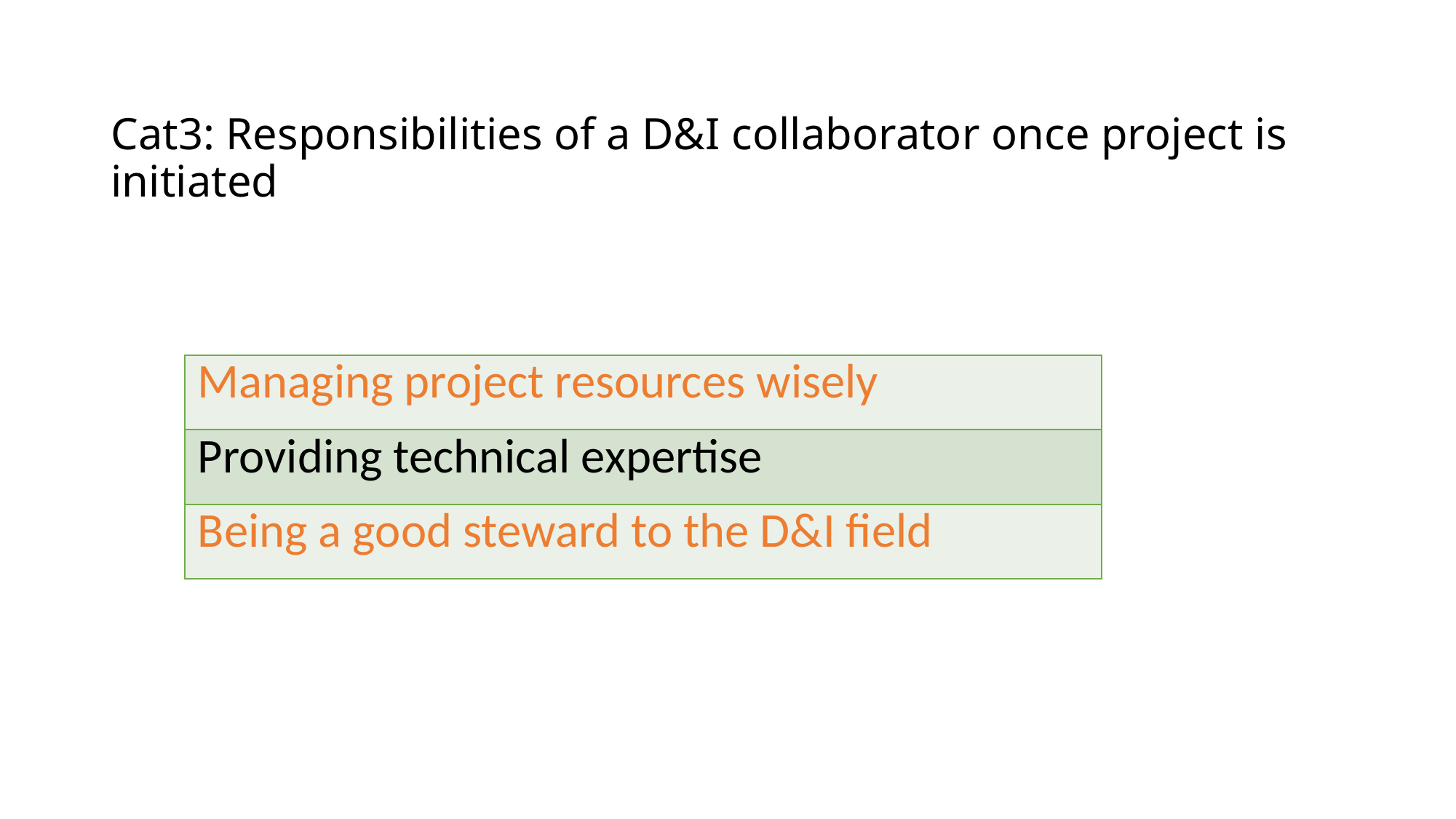

# Cat3: Responsibilities of a D&I collaborator once project is initiated
| Managing project resources wisely |
| --- |
| Providing technical expertise |
| Being a good steward to the D&I field |
